# A Meta-analysis Exploring the Efficacy of Neuropathic Pain Medication for Low Back Pain or Spine-Related Leg Pain: Is Efficacy Dependent on the Presence of Neuropathic Pain

**DOI:** 10.1007/s40265-024-02085-6

**Published:** 2024-10-26

**Authors:** Jennifer Ward, Anthony Grinstead, Amy Kemp, Paula Kersten, Annina Schmid, Colette Ridehalgh

**Affiliations:** Kent Community NHS Foundation Trust, Sevenoaks Hospital, Hospital Road, Sevenoaks, Kent, TN11 3PG, 07973534272, Consultant physiotherapist; Sussex Community NHS Foundation Trust, Trust HQ Brighton General Hospital Elm Grove Brighton BN2 3EW, physiotherapist; University Hospital Sussex, Worthing Hospital, Lyndhurst Road, BN11 2DH, physiotherapist; University of Suffolk, 19 Neptune Quay, Ipswich, IP4 1QJ, UK; Nuffield Department of Clinical Neurosciences, Oxford University, John Radcliffe Hospital, Oxford OX3 9DU, UK; School of Life Course & Population Sciences Faculty of Life Sciences & Medicine King’s College London Guy’s Campus, Addison House SE1 1UL, London, UK; School of Sport and Health Science, University of Brighton, Robert Dodd Building, 49 Darley road, Eastbourne BN20 7UR, UK Department of Clinical Neuroscience, Brighton and Sussex Medical School, Trafford Centre, University of Sussex, Falmer, Brighton BN1 9RY, UK

## Abstract

**Background and Objective:**

Highly variable pain mechanisms in people with low back pain or spine-related leg pain might contribute to inefficacy of neuropathic pain medication. This meta-analysis aimed to determine how neuropathic pain is identified in clinical trials for people taking neuropathic pain medication for low back pain or spine-related leg pain and whether subgrouping based on the presence of neuropathic pain influences efficacy.

**Methods:**

EMBASE, MEDLINE, Cochrane Central, CINAHL [EBSCO], APA PsycINFO, ClinicalTrials.gov, and the World Health Organization International Clinical Trials Registry were searched from inception to 14 May, 2024. Randomized and crossover trials comparing first-line neuropathic pain medication for people with low back pain or spine-related leg pain to placebo or usual care were included. Two independent authors extracted data. Random-effects meta-analyses of all studies combined, and pre-planned subgroup meta analyses based on the certainty of neuropathic pain (according to the neuropathic pain Special Interest Group [NeuPSIG] neuropathic pain grading criteria) were completed. Certainty of evidence was judged using the grading of recommendations assessment development and evaluation [GRADE] framework.

**Results:**

Twenty-seven included studies reported on 3619 participants. Overall, 33% of studies were judged unlikely to include people with neuropathic pain, 26% remained unclear. Only 41% identified people with possible, probable, or definite neuropathic pain. For pain, general analyses revealed only small effects at short term (mean difference [MD] − 9.30 [95% confidence interval [CI] − 13.71, − 4.88], I2 = 87%) and medium term (MD − 5.49 [95% CI − 7.24, − 3.74], I2 = 0%). Subgrouping at short term revealed studies including people with definite or probable neuropathic pain showed larger effects on pain (definite; MD − 16.65 [95% CI − 35.95, 2.65], I2 = 84%; probable; MD − 10.45 [95% CI − 14.79, − 6.12], I2 =20%) than studies including people with possible (MD − 5.50 [95% CI − 20.52, 9.52], I2 = 78%), unlikely (MD − 6.67 [95% CI − 10.58, 2.76], I2 = 0%), or unclear neuropathic pain (MD − 8.93 [95% CI − 20.57, 2.71], I2 = 96%). Similarly, general analyses revealed negligible effects on disability at short term (MD − 3.35 [95% CI − 9.00, 2.29], I2 = 93%) and medium term (MD − 4.06 [95% CI − 5.63, − 2.48], I2 = 0%). Sub-grouping at short term revealed larger effects in studies including people with definite/probable neuropathic pain (MD − 9.25 [95% CI − 12.59, − 5.90], I2 = 2%) compared with those with possible/unclear/unlikely neuropathic pain (MD −1.57 [95% CI − 8.96, 5.82] I2 = 95%). Medium-term outcomes showed a similar trend, but were limited by low numbers of studies. Certainty of evidence was low to very low for all outcomes.

**Conclusions:**

Most studies using neuropathic pain medication for low back pain or spine-related leg pain fail to adequately consider the presence of neuropathic pain. Meta-analyses suggest neuropathic pain medication may be most effective in people with low back pain or spine-related leg pain with a definite/probable neuropathic pain component. However, the low to very low certainty of evidence and poor identification of neuropathic pain in most studies prevent firm recommendations.

## 1 Introduction

Low back pain (LBP) with or without leg pain is the single largest cause of activity limitation and work absence across the world [1] and the leading cause of years lived with disability [2]. More than two thirds of patients with LBP will experience associated spine-related leg pain (“sciatica”) which results in increased pain severity and disability [3]. Acute episodes of LBP and spine related leg pain may resolve within 12 weeks [4], however up to 25% of people still experience significant symptoms at 5 years [5]. Low back and spine-related leg pain can be associated with different underlying pain mechanisms (e.g., nociceptive, nociplastic, neuropathic, mixed) [6]. Neuropathic Pain is common [7, 8] and is often associated with increased symptom severity and exacerbated economic burden compared to nociceptive pain [9].

Most international guidelines recommend medication as either first- or second-line options [10, 11] and include several classes of medication with different mechanisms of action. Recommendations for neuropathic pain medication, however, vary considerably between guidelines[10, 12–14]. The neuropathic pain Special Interest Group (NeuPSIG a subgroup of the International Association for the Study of Pain) guidelines recommend first line medications regardless of underlying etiology. These include serotonin–norepinephrine reuptake inhibitors (SNRI), tricyclic antidepressants (TCA), and anticonvulsants [15]. In contrast, more recently the UK National Institute of clinical excellence (NICE) specifically recommends against prescribing neuropathic pain medication for patients with spine-related leg pain [13, 16]. Similarly, the American college of physicians downgraded all medication for chronic LBP to second line treatments [14]. These conflicting recommendations may in part be explained by the emergence of new evidence over time, however recent systematic reviews (some of which published since the updated low back and spine related leg pain guidelines) suggest that the effectiveness of neuropathic pain medications is not generally dependent upon the underlying aetiology [15, 17, 18]. Instead, neuropathic pain is proposed as a distinct clinical entity [19] which should have targeted management regardless of underlying structural pathology. Given the high prevalence of neuropathic pain in patients with LBP [20] and spine related leg pain [7], instigating treatments for neuropathic pain in this patient group would seem reasonable.

The National Institute of Clinical Excellence [16] and American College of Physicians guidelines [14] for neuropathic pain medications in LBP and spine related leg pain are influenced by the evidence that demonstrates antidepressants and anticonvulsants are no more effective than placebo [21–26] or usual care [22, 25]. However many of the clinical trials have a high risk of bias, and significant heterogeneity [21–26]. Highly variable pain mechanisms in people with LBP or spine related leg pain [27] are likely to contribute to this heterogeneity. It is currently unclear however, if or how clinical trials identify the presence of neuropathic pain in their study population, and whether the presence or certainty of neuropathic pain influences the efficacy of neuropathic pain medication. The aim of this review therefore is twofold. Firstly, to determine how neuropathic pain is identified and secondly, whether subgrouping studies based on the presence of neuropathic pain influences efficacy of neuropathic pain medication in LBP and spine related leg pain.

## 2 Methods

We prospectively registered this review on Prospero CRD42022342027 and followed the PRISMA reporting guidelines (Electronic Supplementary Material [ESM] [28]. A single investigator (JW) searched EMBASE, MEDLINE, CENTRAL, CINAHL (EBSCO), APA PsycINFO, databases from inception to May 9th, 2022, and updated on 14th May 2024. Ongoing trials were searched for in clinical trials.gov and WHO International Clinical trials registry and data requests sent to authors of studies <3years old. The search strategy (ESM) was developed with a medical librarian. The identified references were imported to EndNote referencing software 20.2 (Clarivate, CA, US, Philadelphia) and duplicates were removed.

### 2.1 Study selection

Two authors (JW and AK) independently screened 10% of title and abstracts to ensure agreement on inclusion (QCTSTAR recommendations) [29]. Agreement exceeded the≥90% threshold [29] and the remaining title/abstracts were thus screened by one reviewer (JW). All eligible full text articles were screened by two independent authors (JW and AK). Disagreements in selection were resolved by mediation with a third reviewer (AG) [30]. The updated search was screened by two authors (JW and AS)

Randomised controlled and crossover trials using neuropathic pain medication of any duration for participants with any duration of LBP with or without spine related leg pain were included. Eligible neuropathic pain medications were commonly prescribed first line oral medications as recommended by NeuPSIG guidelines (SNRI, including Duloxetine or Venlafaxine; TCAs, including Amitriptyline, Nortriptyline or Desipramine and anticonvulsants, including Gabapentin or Pregabalin) [15, 17]. Comparators were placebo or usual care. As most medication studies allow add-on or rescue medication, usual care could include other pain medications, if both groups had the same access to this medication. We therefore also included comparative effectiveness studies, if both groups took a matching dose of a non-neuropathic pain medication (A) and one group had an additional neuropathic pain medication added (A plus B). Exclusion criteria included other comparative effectiveness studies (i.e., where doses of the non-neuropathic pain medication (A) differed between the two treatment groups, or two different medications were directly compared, with no control); studies not published in English; case series and conference abstracts. Studies including participants with the following were also excluded; pregnancy; recent surgery or spinal injection (within last 3 months); central nervous system pathology; peripheral neuropathy not related to the spine; depression-related pain, where clinical depression was a participant inclusion criterion; and multiple areas of pain (i.e., neck pain and LBP) if separate data could not be obtained. A list of excluded studies at the full-text screening stage can be found in the ESM.

### 2.2 Data Extraction

Two authors (JW and AG) extracted data independently into a standardised excel spreadsheet developed and piloted by the study team [30]. Consensus was used to resolve any discrepancies. In addition to study characteristics (i.e. age, pain duration, severity etc.), for the first aim (to determine how neuropathic pain is identified in studies) we extracted any methods and results used to determine the presence of neuropathic pain. Two authors (JW and AG) independently mapped this to the certainty grading of neuropathic pain (unlikely/possible/probable/definite neuropathic pain) according to the revised NeuPSIG criteria [31] adjusted for spine-related leg pain (at study not participant level) (Table 1). We added an additional judgement of unclear, where not enough information was provided. We determined the intertester reliability of applying the NeuPSIG grading system at study level with weighted Cohen’s kappa [32].

For the second aim, (to determine whether subgrouping studies based on certainty of neuropathic pain influences medication efficacy) two authors (JW, AG) extracted post treatment pain and disability means, within group change scores and variance measures for treatment and control groups. If any data were missing, we first contacted authors, and if no response, we extracted data from figures using plot digitizer (https://plotdigitizer.com) where possible. Details of any data extracted from figures was recorded in table 2.

Primary outcomes were average leg pain intensity (or back pain, where leg pain was not reported) [3, 43] and disability outcomes. Where possible, outcomes were grouped by timepoints into a) short term (<12 weeks); b) medium term (≥12 weeks, <52 weeks); c) long-term (≥ 52 weeks). If multiple outcomes were reported within one period, the outcome closest to 7 weeks, 6 months and 12 months was used. Note was made of the primary endpoint of each study.

For meta-analysis, variance measures were converted to estimated standard deviations using the Cochrane RevMan calculator [44, 45]. When it was not possible to estimate standard deviations, we borrowed them from a similar study included in the review, as per Cochrane recommendations [46]. In studies with a placebo arm compared to several different treatment arms (i.e. using different doses of the same drug), the placebo results were divided by the number of treatment arms [46]. For cross over trials, we could not confine data extraction to the pre-crossover data owing to the lack of data reporting. Therefore, we took measurements from the treatment and control periods and analysed these as if the study was a parallel group design. Study results were adjusted to correct for the correlation between the two phases [47].

### 2.3 Statistical Analysis

Overall meta-analyses were performed for each timepoint using Revman v5.4.1., if data were available from at least two studies using similar outcomes. Final values or change scores for pain and disability were transformed to scales out of 100 to allow for comparison using mean difference (MD) calculations, where 100 represents worst pain or disability [46]. The DerSimonian and Laird [48] method for random effects models and inverse variance weighting methods were used to account for the variability of included studies. Statistical significance between treatment and control groups was determined using t-tests with a pre-registered significance cut-off of p< 0.05. Sidik-Johnkman estimator for tau^2^ adjusted for between study variances.

Rather than selecting an arbitrary minimally clinically important difference threshold (which dichotomises outcomes into ‘worthwhile’ versus ‘not worthwhile’ effects) [49] effect estimates for pain and disability were categorised as negligible (<5), small (5-9), moderate (10-19) or large (>20) [50].

#### 2.3.1 Pre-planned subgroup analysis

We performed a pre-planned subgroup analysis of our estimates of treatment effect at each follow up timepoint (short, medium and long term) to explore the influence of the certainty of neuropathic pain on medication efficacy. If subgroups were too small (e.g., less than two studies in two or more neuropathic pain groups), we used a pre-planned clustering of pain groups. The NeuPSIG guidelines recommend the level “probable” as sufficient to initiate neuropathic pain treatment [19], therefore we grouped studies including participants with unlikely/unclear/possible neuropathic pain versus studies including participants with probable/definite neuropathic pain.

A pre-planned meta-analysis was performed on the type of medication (TCAs, SNRIs, anticonvulsants). Because of limited studies per timepoint preventing time specific meta-analyses, we post-hoc adapted our protocol to perform analyses on the primary end points (or timepoint closest to end of treatment), grouped by neuropathic pain certainty as above. Results that could not be included in the meta-analysis were narratively described. A cutoff value of p<0.1 was used to identify subgroup differences [51]

#### 2.3.2 Post-hoc meta-regression

As part of an exploratory post hoc analysis, we used meta-regression if a minimum of ten studies was available per covariate [47, 52, 53]. Restricted maximum likelihood for random effects was used to apply univariate meta-regressions [54] on our main factor of interest; neuropathic pain certainty (grouped by two clusters). P<0.05 was considered significant.

### 2.4 Quality assessment

Two authors (JW, AG) rated the risk of bias of pain and disability outcomes using the revised Cochrane collaboration risk of bias tool (RoB2) [55]. Disagreements were resolved by mediation with a third reviewer if required (AK). The overall certainty of evidence was assessed by one reviewer (JW) using the grading of recommendations assessment development and evaluation (GRADE) approach [56]. Details of the GRADE criteria are illustrated in the ESM.

## 3 Results

### 3.1 Study descriptions

**Fig. 1:**
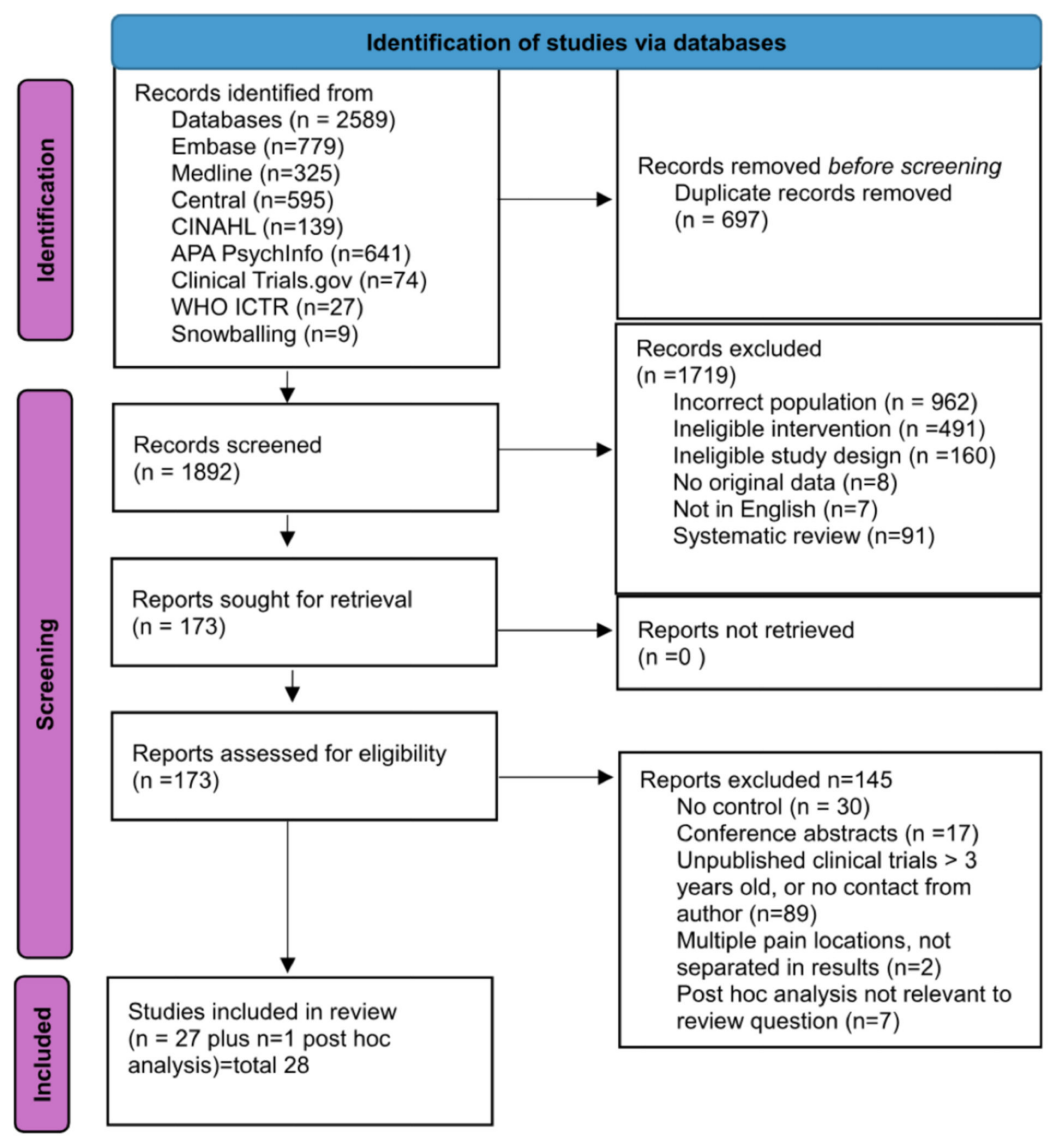
PRISMA diagram Preferred Reporting Items for Systematic reviews and Meta-Analyses (PRISMA) diagram. ICTR International Clinical Trials Registry, WHO World Health Organization

As illustrated in Fig. 1, *n=*1892 non-duplicate citations for title or abstract screening were identified. *N=*173 articles were screened for full text eligibility. See ESM for reasons for exclusion at full text screening stage. The final systematic review and narrative synthesis included *n=*27 studies [57–83] summarising findings from *n=*3619 participants commencing trial protocol and 2939 completing trial protocol. Table 2 lists the study level characteristics.

### 3.2 Risk of Bias and certainty of evidence

**Fig. 2:**
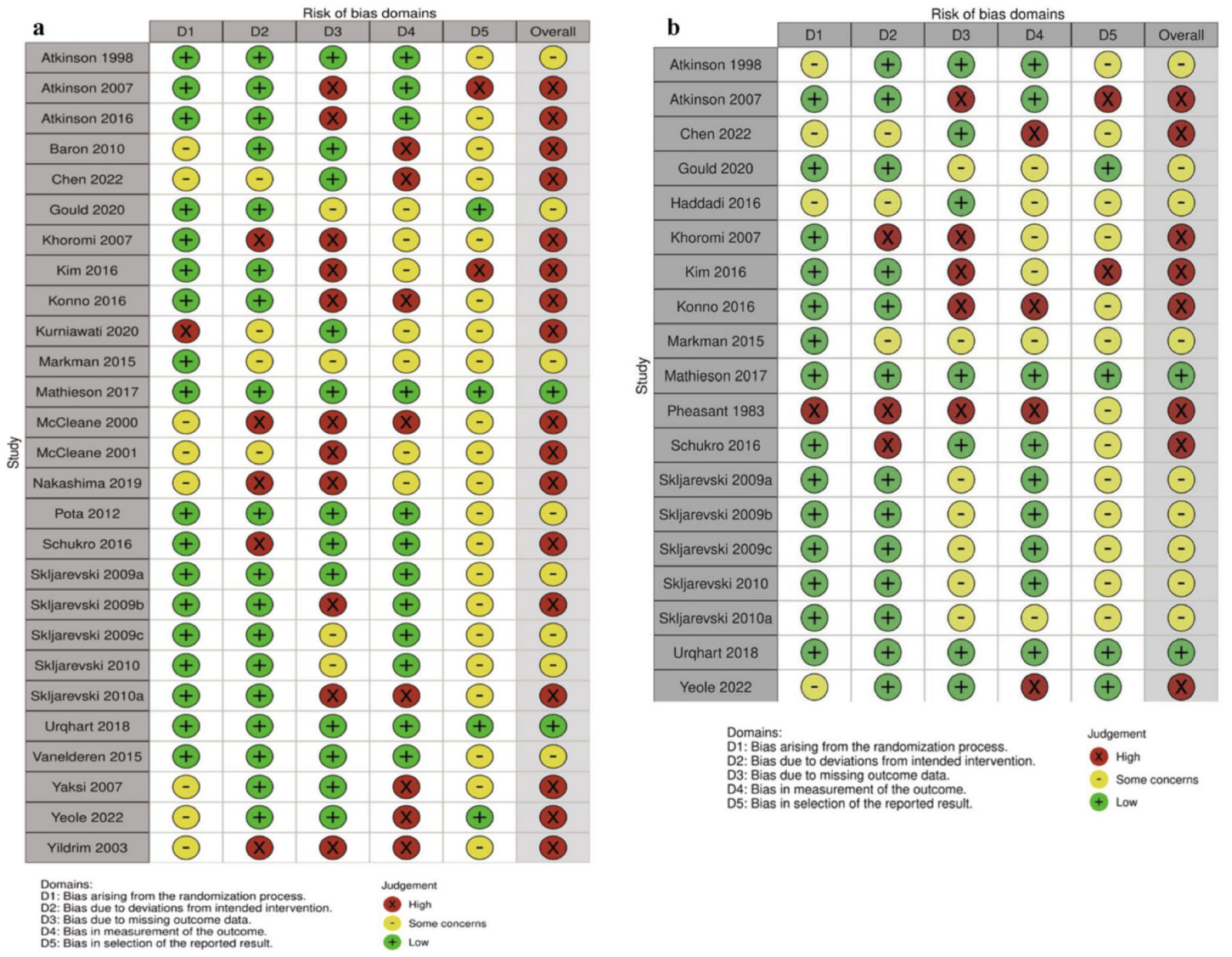
Risk of bias(2) assessment of (A) pain and (B) disability outcomes

For pain, high risk of bias was found in 17 studies [57, 58, 61, 62, 64, 67–69, 72–75, 77, 79, 80, 82, 83] (Figure 2A). For disability, high risk of bias was found in eight studies [64, 66, 73–75, 79, 82, 83] (Figure 2B). Overall certainty of evidence was assessed using GRADE (ESM) and is referred to within the results for each outcome. Visual inspection of funnel plots for outcomes with more than ten studies suggested that the effects were evenly distributed around the mean (ESM).

Table 3 summarises judgements of the certainty of neuropathic pain based on NeuPSIG grading criteria.[31]. There was substantial interrater agreement for the NeuPSIG grading between the two investigators (weighted Cohen’s kappa=0.807 (95% confidence interval (CI) 0.660 to 0.954). Overall, nine studies [60–63, 68, 69, 73, 78, 80] were judged as unlikely to include participants with neuropathic pain. Many of these purposefully excluded participants with symptoms of neuropathic pain such as shooting pains, paraesthesia, numbness and allodynia [68, 69]; positive neurological signs on bedside examination [80]; radiological evidence of nerve compression [61–63]; and electromyography changes [63]. Several studies attempted to identify neuropathic pain at recruitment, (i.e. through pain descriptors and bedside examination [78] or painDETECT questionnaire [60] but did not specify these as inclusion criteria, and subsequently less than 25% of included participants actually had signs of neuropathic pain at recruitment.

Seven studies were judged as unclear inclusion of participants with neuropathic pain, as they used broad pain related inclusion criteria with insufficient baseline information [65, 66, 71, 72, 76, 79, 82]. Four of these studies [66, 72, 76, 79] excluded participants with spine related leg pain, however this alone was not considered an exclusion of neuropathic pain as there is a high prevalence of neuropathic pain in axial LBP [85].

The certainty of neuropathic pain was judged to be possible in five studies [64, 70, 74, 75, 77] where neuropathic pain descriptors and screening tools were used either as inclusion, or in baseline reporting. Several studies also included bedside examination findings, but at recruitment either failed to specify these as inclusion criteria or report baseline characteristics, which limited their progression up the grading scale [70, 75].

The certainty of neuropathic pain was judged to be probable in four studies [59, 67, 81, 83]. All studies found changes suggestive of nerve compression on MRI in all participants, but either failed to assess bedside sensory examination [81] or failed to demonstrate sensory changes in more than 50% of the participants [59, 67, 81] and were therefore downgraded from definite to probable inclusion of participants with neuropathic pain [19, 31]. Finally, two studies [57, 58] were judged as including participants with definite neuropathic pain through the use of pain descriptors, bedside examination and diagnostic tests [57, 58].

## 4 Meta analysis

For the second aim, (is efficacy dependent on certainty of neuropathic pain) three [66, 74, 77] of the 27 studies were excluded from the meta-analysis. Two of these studies had limited reporting of data (baseline [66], or variance measures [74]). One study used a controlled withdrawal design, where results were not comparable with a randomised controlled design [77] (ESM). The meta-analysis therefore included 24 [57–65, 67–73, 75, 76, 78–83] studies with a total of 3264 participants recruited, and 2660 completing the studies. During subgrouping by certainty of neuropathic pain, the results from one study [60] was substituted with the results of a post hoc analysis, by the same author [84] who sub grouped their original study data according to neuropathic pain scores.

### 4.1 Pain outcomes

**Fig. 3:**
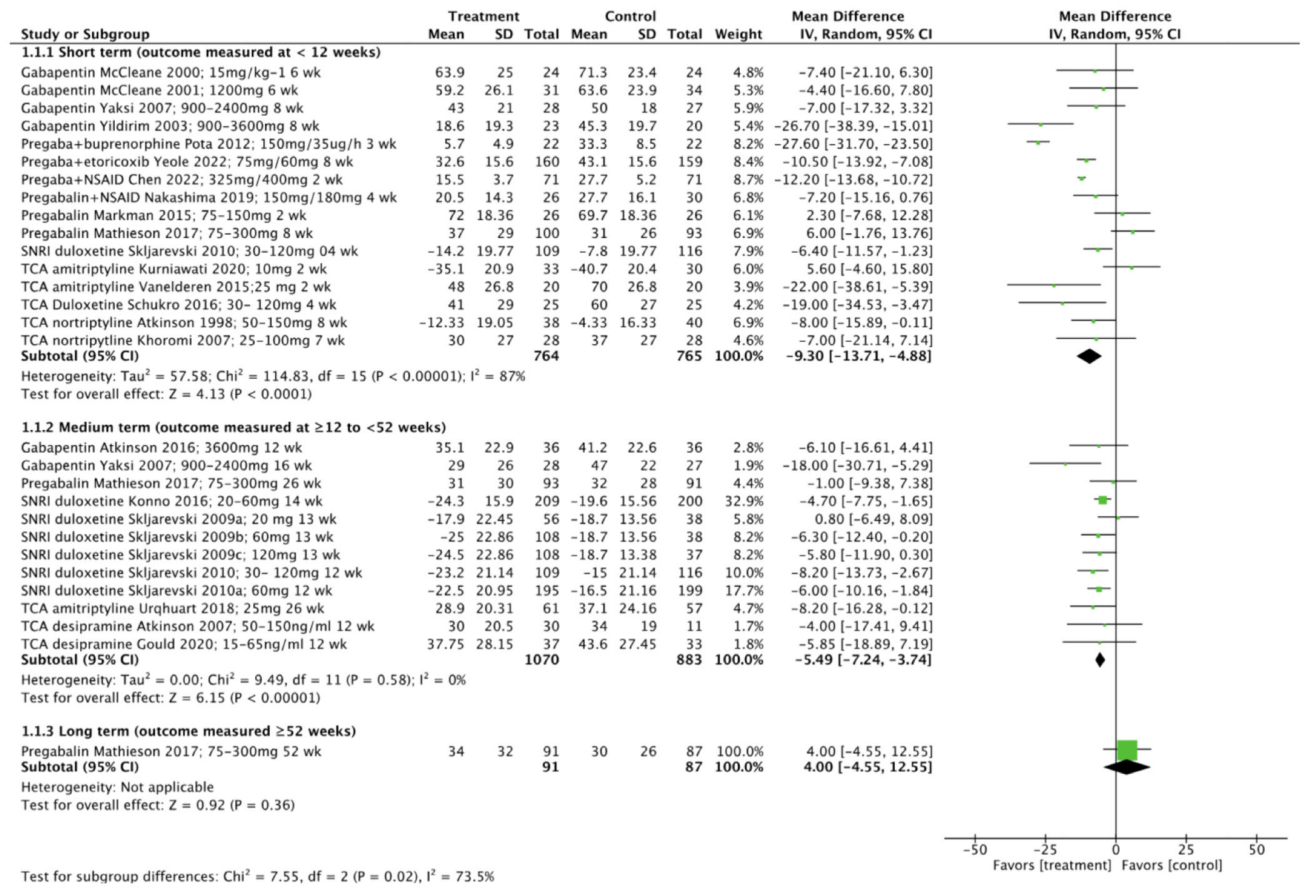
Pain outcomes overall, reported at short-term, medium-term, and long-term timepoints. (CI confidence interval, NSAID non-steroidal anti-inflammatory drug, SD standard deviation, SNRI serotonin-norepinephrine reuptake inhibitors, TCA tricyclic antidepressant, wk weeks)

For pain, overall meta-analysis (fig. 3) revealed very low certainty evidence of a small effect in favour of treatment compared to control at short term (MD-9.30, 95% CI -13.71 to -4.88, I^2^=87%) [57–59, 63–65, 67–72, 75, 78, 82, 83]and medium term timepoints (MD-5.49, 95% CI -7.24 to -3.74, I^2^=0%). [58, 60–63, 70, 73, 76, 79, 80] Meta-analysis at long term was not possible overall or in subgroup analysis as only one study [70] reported outcomes (MD 4.00 (95% CI -4.55 to 12.55).

**Fig. 4:**
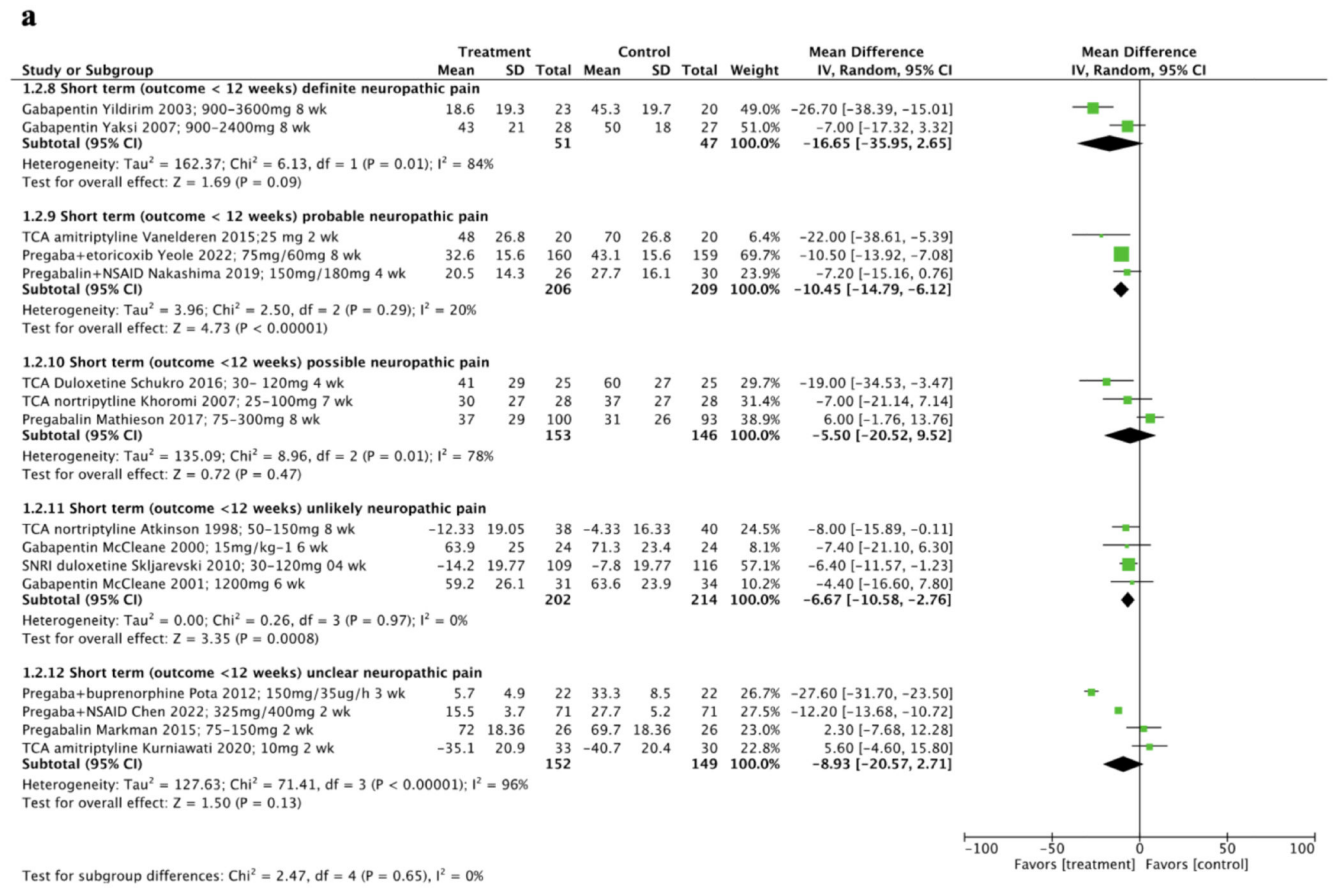

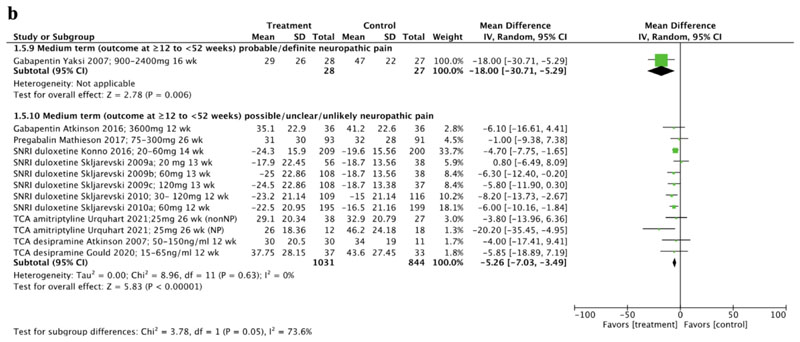
A Pain outcomes reported at short-term timepoints, sub grouped by certainty of neuropathic pain (definite/probable/possible/unclear/unlikely neuropathic pain). (CI confidence interval, NSAID non-steroidal anti-inflammatory drug, SD standard deviation, SNRI serotonin-norepinephrine reuptake inhibitors, TCA tricyclic antidepressant, wk weeks) Fig. 4B Pain outcomes reported at medium-term timepoints, subgroups clustered by certainty of neuropathic pain (definite/probable vs possible/unclear/unlikely neuro- pathic pain). (CI confidence interval, NSAID non-steroidal anti-inflammatory drug, SD standard deviation, SNRI serotonin-norepinephrine reuptake inhibitor, TCA tricyclic antidepressant, wk weeks)

A subgroup analysis of pain at short term (Fig. 4A), demonstrated larger (albeit still moderate) effects in favour of treatment compared to control in participants with definite neuropathic pain [57, 58] (MD-16.65, 95% CI -35.95 to 2.65, I^2^=84%), or probable neuropathic pain [59, 67, 83] (MD-10.45, 95% CI -14.79 to -6.12, I^2^=20%), the latter comparison reaching statistical significance. In comparison, only small effects were found in favour of treatment compared to control in studies including possible [64, 70, 75] (MD-5.50, 95% CI-20.52 to 9.52, I^2^=78%) unlikely [63, 68, 69, 78] (MD-6.67, 95% CI -10.58 to 2.76, I^2^=0%) or unclear [65, 71, 72, 82]neuropathic pain (MD-8.93, 95% CI-20.57 to 2.71, I^2^=96%). There were however no significant differences between subgroups (p=0.65) and certainty of evidence was very low for all outcomes.

For subgroup analysis of pain at medium term (Fig. 4B), there were less than two studies in two or more subgroups (i.e. in the definite and probable subgroup). Therefore, as part of a preplanned analysis, we combined the definite and probable subgroups and compared them to a combined possible, unlikely or unclear neuropathic pain subgroup. Despite this, the definite/probable subgroup still only contained one study, preventing robust subgroup comparisons. The one study including participants with definite neuropathic pain showed moderate effects on pain in favour of treatment compared to control [58] (MD -18.00, 95% CI - 30.71 to -5.29) while only small effects were found for combined data of studies including participants with possible/unclear/unlikely neuropathic pain [61–63, 70, 73, 76, 79, 80, 84] (MD-5.26, 95% CI -7.03 to -3.49, I^2^=0%). Significant subgroup differences were detected (p=0.05) but the uneven distribution of studies between subgroups and very low certainty of evidence limits interpretation [51]. No subgroup analyses of pain at long-term could be performed as only one study reported on this timepoint.

### 4.2 Disability outcomes

**Fig. 5:**
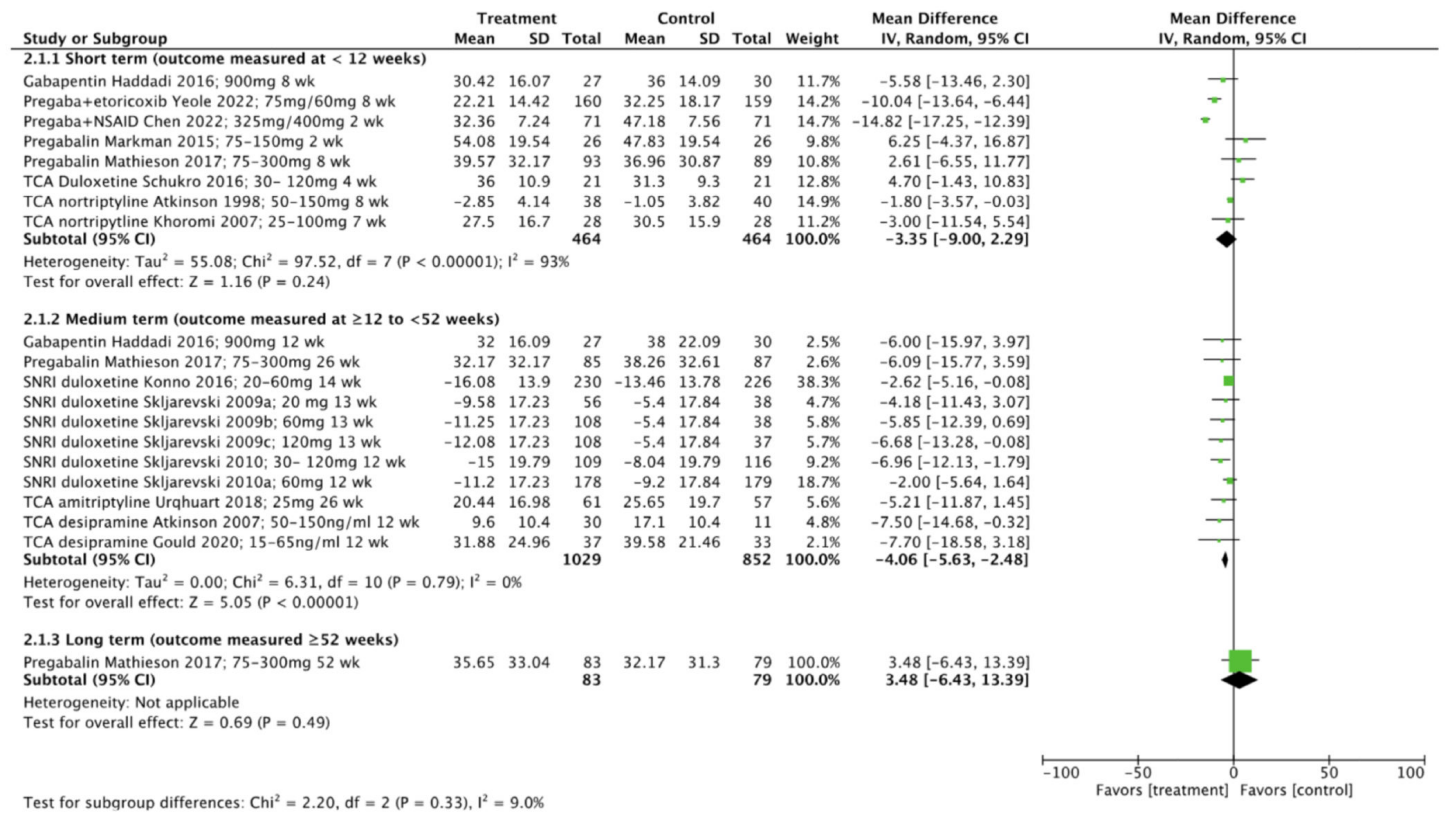
Disability outcomes overall, reported at short-term, medium-term, and long-term timepoints. (CI confidence interval, NSAID non-steroidal anti-inflammatory drug, SD standard deviation, SNRI serotonin-norepinephrine reuptake inhibitors, TCA tricyclic antidepressant, wk weeks)

For disability, as demonstrated in Figure 5, overall meta-analysis revealed low to very low certainty evidence of negligible effects in favour of compared to control at short term (MD -3.35 (95% CI -9.00 to 2.29), I^2^=93%) [64, 70, 71, 75, 78, 81–83] and medium term timepoints (MD-4.06 (95% CI -5.63 to -2.48), I^2^=0%) [60–63, 70, 73, 76, 79, 81]. Meta-analysis at long term was not possible overall or in subgroup analysis as only one study [70] reported outcomes (MD 3.48 (95% CI -6.43 to 13.39)).

**Fig. 6:**
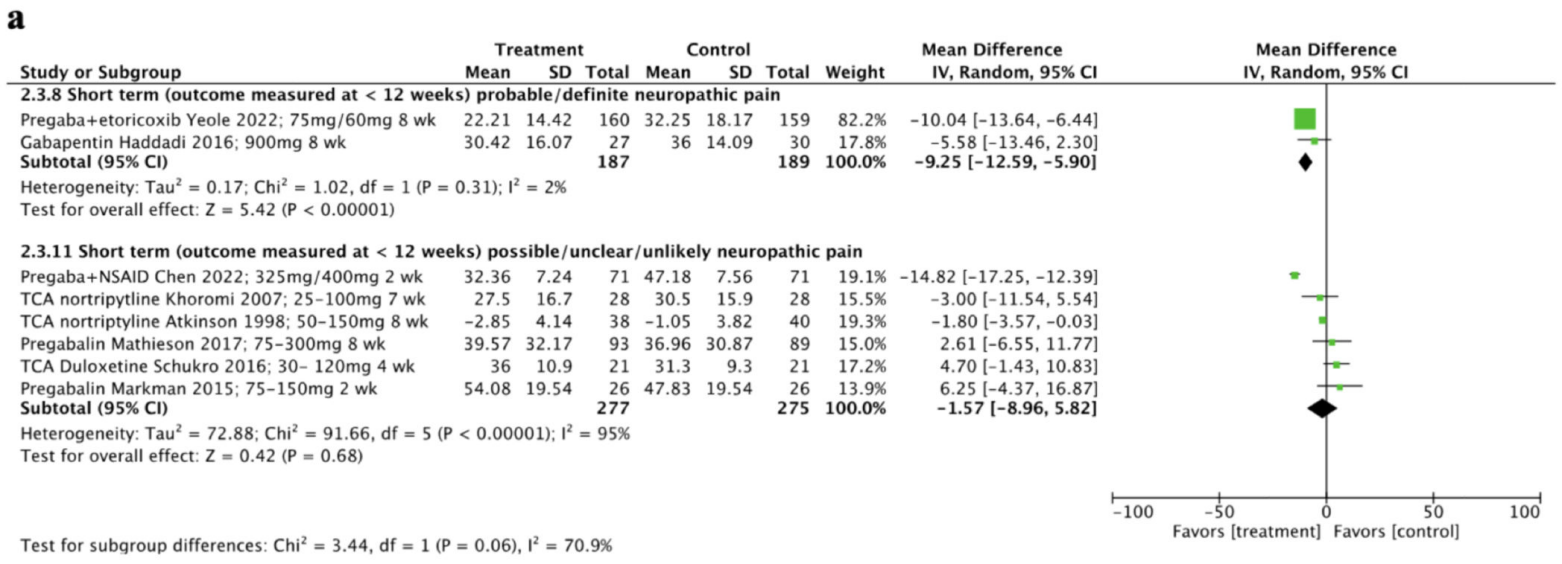

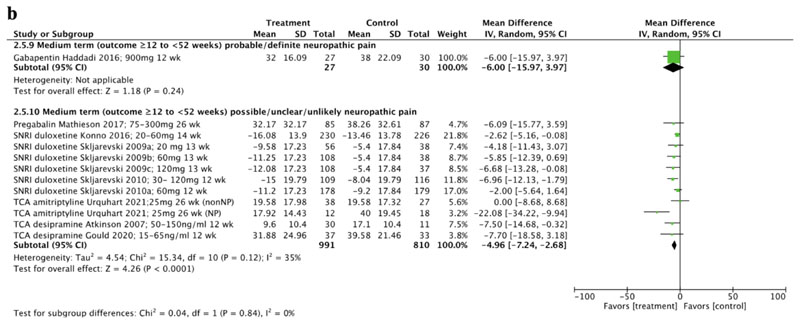
A Disability outcomes reported at short-term timepoints, subgroups clustered by certainty of neuropathic pain (definite/probable vs possible/unclear/unlikely neuropathic pain). (CI confidence interval, NSAID non-steroidal anti-inflammatory drug, SD standard deviation, SNRI serotonin-norepinephrine reuptake inhibitor, TCA tricyclic antidepressant, wk weeks) Fig 6B Disability outcomes reported at medium-term timepoints, subgroups clustered by certainty of neuropathic pain (definite/probable vs possible/unclear/unlikely neuropathic pain). (CI confidence interval, NSAID non-steroidal anti-inflammatory drug, SD standard deviation, SNRI serotonin-norepinephrine reuptake inhibitors, TCA tricyclic antidepressant, wk weeks)

For the subgroup analysis of disability at short term (Figure 6A), we again combined the data from definite and probable subgroups due to limited study numbers, and compared this with a combined possible, unlikely or unclear subgroup. Sub-grouping demonstrated larger (albeit still small) effects in favour of treatment compared to control, in studies including participants with definite/probable neuropathic pain [81, 83] (MD -9.25 (95% CI-12.59 to -5.90), I^2^=2%). In comparison, negligible effects were found in favour of treatment compared to control at short term in studies including participants with possible/ unclear/unlikely neuropathic pain [64, 70, 71, 75, 78, 82] (MD -1.57 (95% CI -8.96 to 5.82), I^2^=95%). There was a significant difference between subgroups (*p*=0.06), however this analysis is limited by the uneven distribution of studies between subgroups [51] and the very low certainty of evidence.

For the subgroup analysis of disability at medium term (Figure 6B), after combining subgroups as above, only one study [81] remained in the definite/probable subgroup. This one study showed no significant difference between treatment and control (MD -6.00 (95% CI -15.97 to -3.97). Combined data from studies including participants with possible/unclear/unlikely neuropathic pain [61–63, 70, 73, 76, 79, 84]showed negligible effects in favour of treatment compared to control (MD -4.96 (95% CI -7.24 to - 2.68), I^2^=35%). There were no significant differences between subgroups (*p*=0.84) and certainty of evidence was low.

### 4.3 Type of medication (post hoc analysis)

#### 4.3.1 TCA medication

A subgroup analysis of TCA effects on pain (after combining subgroups) revealed only one study in the definite/probable neuropathic pain group. This study included participants with probable neuropathic pain [59] and showed large effects on pain in favour of treatment (MD-22 (95% CI -38.61 to -5.39)). Combined data from studies including participants with possible/unlikely/unclear neuropathic pain [72, 75, 76, 78, 79, 84] showed no significant difference between treatment and control (MD -5.32 (95% CI -10.55 to -0.09) I^2^=31%) (ESM). Significant subgroup differences (*p*=0.06) were identified but interpretation again limited by uneven subgroups [51] and very low certainty of evidence. The subgroup analysis of TCA effects on disability (after combining subgroups) revealed no studies included participants with definite/probable neuropathic pain, preventing meta-analysis. Combined data from studies including participants with possible/unlikely/unclear neuropathic pain [75, 76, 78, 79, 84] showed small effects on disability (MD-5.55 (95% CI-10.39 to 0.71), I^2^=63%) (ESM) with low certainty of evidence.

#### 4.3.2 Anticonvulsants

The subgroup analysis of anticonvulsants effects on pain (after combining subgroups) revealed studies including participants with definite/probable neuropathic pain [57, 58, 67, 83] showed moderate effects in favour of treatment (MD -11.81 (95% CI-18.32 to - 5.31) I^2^= 64%). In contrast, combined data from studies including participants with possible/unlikely/unclear neuropathic pain [65, 68–71, 80, 82] showed no significant difference between treatment and control (MD-7.63 (95% CI -16.17 to -0.91), I^2^=93%) (ESM). There was no subgroup difference (p=0.44) and certainty of evidence was very low. The subgroup analysis of anticonvulsants effects on disability (after combining subgroups) revealed a similar trend of larger (albeit small) effects in studies including participants with definite/probable neuropathic pain [81, 83] (MD -9.25 (95% CI -12.59 to -5.90), I^2^=2%) compared to combined data from studies including participants with possible/unlikely/unclear neuropathic pain [70, 71, 82] (MD -2.60 (95% CI -17.78 to 12.57) I^2^=92%) (ESM) There was no subgroup difference (p=0.40) and certainty of evidence was low to very low.

#### 4.3.3 SNRI’s

Subgroup analysis of SNRI effects on pain and disability (after combining subgroups) revealed no studies included participants with definite/probable neuropathic pain, preventing meta-analysis. Combined data from studies including participants with possible/unlikely/unclear neuropathic pain [61–64, 73] showed small effects on pain (MD-5.57 (95% CI-7.75 to -3.39), I^2^=15%) (ESM) and negligible effects on disability (MD-3.16 (95% CI-5.64 to -0.67), I^2^=44%) (ESM) with low to moderate certainty of evidence.

### 4.4 Meta-regression (post hoc analysis)

Because of the small numbers of studies, a univariate analysis was possible only for pain at short term and medium term, disability at medium term, and anticonvulsant pain outcomes (see ESM). A meta-regression compared the outcomes of sub-grouped studies including participants with definite/probable neuropathic pain against the reference (studies including participants with possible/unclear/unlikely neuropathic pain). Results showed no effect of neuropathic pain certainty on pain outcomes at short term (coefficient -6.367 (95% CI−17.348 to 4.615) p=0.256). For pain at medium term, there was a trend for an effect of neuropathic pain group on pain outcomes (coefficient-12.75 (95% CI −25.59 to 0.08) p=0.051). For disability at medium term, meta-regression revealed no significant effect (coefficient -1.819 (−12.044 to 8.407) p=0.727). For the anticonvulsant studies, meta-regression also revealed no significant effect of neuropathic pain certainty on pain outcomes (coefficient -4.718 (−18.198 to 8.761), p=0.493).

## 5 Discussion

This systematic review and meta-analysis revealed that most studies using neuropathic pain medication for low back or spine related leg pain failed to adequately consider the presence of neuropathic pain. This is surprising, considering these medications are normally recommended to target neuropathic pain [15]. Strikingly, we found the majority of studies (59%) either specifically excluded participants with neuropathic pain or failed to provide any consideration for neuropathic pain symptoms within the inclusion criteria and baseline reporting. Overall meta-analysis revealed low to very low certainty evidence of only small or negligible effects of neuropathic pain medications on pain and disability at short and medium term compared to placebo or usual care. In contrast, subgrouping studies by the presence of neuropathic pain revealed larger yet still at best, moderate to small effects on pain and disability respectively at short and medium term in studies including participants with definite or probable neuropathic pain, while effects remained small or negligible for those studies including participants with unclear/unlikely/possible neuropathic pain. When subgroups according to type of medication were explored, very low certainty evidence suggested a trend that studies including participants with definite/probable neuropathic pain had superior pain outcomes to anticonvulsant or TCA medication than studies with participants with possible/unclear/unlikely neuropathic pain. However, the certainty of evidence remained low, subgroup differences were not always significant or limited by uneven subgroups and the effect sizes were generally small to moderate. Overall though, the trend towards larger outcomes for pain and disability in studies including participants with definite/probable neuropathic pain suggests that careful neuropathic pain phenotyping may be of value when prescribing neuropathic pain medications.

The idea that stratification by pain mechanism (e.g., neuropathic pain) improves drug efficacy has gained substantial traction in recent years, yet the evidence remains preliminary. Several post hoc analyses support a trend towards improved outcomes in participants with neuropathic pain when taking neuropathic pain medication for LBP and spine related leg pain. Urquhart et al [84] for example, found painDETECT scores of =>13 (possible neuropathic pain [86], plus VAS score of =>30 predicted response to amitriptyline. Similarly, in participants completing an 8 week course of nortriptyline, those with radicular pain had greater pain intensity reduction than those with non-radicular pain [78]. Sensory profiles related to neuropathic pain have also been demonstrated to predict response to medications, such as minocycline following lumbar discectomy [91] and tapentadol for chronic LBP [92]. Clusters of somatosensory profiles identified through quantitative sensory testing [93] predict response to treatment with Pregabalin in HIV neuropathy [94]. There is also evidence that targeting treatment towards pain mechanisms is more effective than targeting etiologies, and this has been demonstrated in several non-spine related neuropathic pain conditions [95–97]. In this review, several studies selected participants by etiology such as lumbar spinal stenosis or neurogenic claudication [58, 71, 74]. It is well established however, that these populations have heterogeneous pain mechanisms; for example, less than 16% of people with neurogenic claudication have neuropathic pain [98].

To our surprise, only 22% of studies included patients with probable or definite neuropathic pain, where first line neuropathic pain medications are recommended [15]. In some circumstances, different neuropathic pain medications can be prescribed for non-neuropathic pain conditions such as chronic primary pain. Antidepressants, for example, are recommended to treat chronic primary LBP [99] which is a diagnosis of exclusion, often based on the absence of nerve compression on tests such as MRI [100]. It is possible that some studies were aiming to target primary chronic pain rather than neuropathic pain. However, we felt this was unlikely given none of the studies identified primary chronic pain in their inclusion criteria, and only three studies specified the absence of nerve compression on MRI for inclusion [61–63].

The retrospective nature of judgements on the presence and certainty of neuropathic pain, by mapping reported inclusion criteria and baseline measures to the neuropathic pain grading criteria has some limitations [31]. There are no existing ‘gold standards’ to diagnose neuropathic pain. Many of the neuropathic pain screening tools used by studies in this review were not originally designed for participants with LBP or spine related leg pain [38, 101] have only moderate sensitivity and specificity when translated to English [102] and correlate poorly with clinicians’ judgement of the presence of neuropathic pain [103]. Furthermore, the requirement within the grading system to confirm definite neuropathic pain through diagnostic tests is questionable, as it is well recognized that MRI results do not always correlate with symptom presentation in participants with LBP or spine related leg pain [104]. It is therefore possible that other pain mechanisms influenced the overall pain outcomes across studies, especially as sciatica is often a mixed pain condition [105]. Reassuringly though, our reliability data suggest the grading system could be reliably applied by two different investigators.

### Limitations

There are limitations that prevent firm conclusions from this review, most notably the low to very low certainty of evidence found in the GRADE assessment across most outcomes. The lack of definition for spine related leg pain [106] is reflected in the broad inclusion criteria and unclear study reporting, which led to a high percentage of studies including participants with unclear neuropathic pain (26%). This potentially biased our results by reducing the power of our subgroup meta-analyses. Only two studies [60, 70] were identified as low risk of bias. Both included patients with possible neuropathic pain, therefore leaving only higher risk studies in the definite/probable neuropathic pain group. High levels of heterogeneity across studies were found, possibly influenced by variability in medication type, control group, doses, rescue medications, duration of treatment and outcomes used. Exploration of this heterogeneity through multivariable meta-regressions was prevented by the small number of studies.

### Clinical implications

International guideline recommendations for neuropathic pain in LBP and spine related leg pain are conflicting, although many advise against prescribing neuropathic pain medications [13, 14]. However, our results suggest very low certainty evidence that small but more meaningful effects can be found in participants with definite/probable neuropathic pain. This is important to explore further, particularly as much of the previous research in this area has failed to consider the presence of neuropathic pain. Treatment options, especially for spine related leg pain remain limited, with a paucity of evidence to support conservative treatment [107, 108] or surgery [109]. Finding the right medication, for the right person, prescribed at the right time may therefore be helpful in managing this complex, multifaceted condition. As a result of the small, or at best, moderate effects of medication, and the high likelihood of side effects, we would encourage clinicians to discuss the risks versus potential benefits of any medication, as part of a shared decision-making conversation [110]. Within that, it is important to acknowledge that the treatment effect represents an average response in study participants, which cannot be used to reliably predict individual patient response, but can serve as an important guide in discussions [110, 111].

## 6 Conclusion

The results of this systematic review and meta-analysis showed that the majority of existing studies using neuropathic pain medication for people with LBP or spine related leg pain do not adequately identify participants with neuropathic pain. Subgroup meta-analysis was limited by low numbers of studies including participants with probable/definite neuropathic pain. Low to very low certainty evidence revealed a trend towards larger (albeit moderate to small) effect sizes on pain and disability at short and medium term in participants with definite/probable neuropathic pain, compared to negligible to small effect sizes in those with possible/unclear/unlikely neuropathic pain.

These results raise questions about current recommendations that advise against prescribing neuropathic pain medication for people with by LBP or spine related leg pain with neuropathic pain. Guidelines based on the outcomes of studies with highly heterogenous patient populations may be premature and further research with careful neuropathic pain phenotyping is warranted. Future research should clearly identify the presence of neuropathic pain in participants, to conclusively determine whether they are indeed more likely to respond to this type of medication. Investigations to determine which clinical phenotypes are likely to benefit from neuropathic pain medication is key to allow clinicians and individuals to make personalised treatment decisions, through shared decision making [110].

## Supplementary Material

supplementary material

## Figures and Tables

**Table 1:** Application of the NeuPSIG grading system to determine the certainty of neuropathic pain

Unclear neuropathic pain	Where trials have not provided enough information on the pain characteristics or examination of participants to determine the certainty of neuropathic pain.
Unlikely neuropathic pain	Where baseline information has been provided but participants characteristics do not meet the possible, probably or definite criteria; OR where the study has actively excluded participants who present with features of neuropathic pain.
Possible neuropathic pain	1, A medical history with clinical presentation suggestive of a neural lesion or disease (e.g., surgery, trauma, neuropathic characteristics or behaviour of symptoms). Symptom descriptions suggestive of possible neuropathic pain included burning or hot sensations, electric shocks or shooting, pricking or pins and needles, pain evoked by light touching or cold, and non-painful sensations such as numbness and tingling [33, 34]. Positive neuropathic pain screening tools [35] (e.g., Self-Complete Leeds Assessment of Neuropathic Symptoms and Signs [S-LANSS], painDETECT[36], STEP[37], Douleur Neuropathique 4 [DN4] [38], and neuropathic pain Questionnaire (NPQ) [39] were also considered suggestive of possible neuropathic pain, but inclusion of these was not considered compulsory. AND 2, Pain in a neuroanatomically plausible distribution (dermatomal pain which reflect the innervation of specific nerve roots were considered suggestive of neuropathic pain, however pain extending beyond traditional dermatomes might also be considered neuroanatomically plausible given the potential for extraterritorial spread) [40].^a^
Probable neuropathic pain	Clinical bedside examination including sensory signs in the relevant neuroanatomical distribution, which typically include loss of function in small or large nerve fibres causing loss of light touch, vibration, pinprick, cold, or warm sensation [31]. Any quantitative sensory testing including loss of thermal and mechanical detection threshold [41].^a^
Definite neuropathic pain	Diagnostic tests including, but not limited to, computed tomography, magnetic resonance imaging, or other imaging techniques such as ultrasound to confirm the presence of a nerve lesion. Skin biopsy showing reduced intraepidermal nerve fibre density, neurophysiological tests such as nerve conduction velocity, heat and laser evoked potentials, nerve excitability tests, sympathetic skin responses, microneurography with evidence of aberrant nociceptor activity [42]. ^a, b^

a Trials reporting participant characteristics suggestive of neuropathic pain in some, but not all participants were judged to have met the grading criteria when ≥50% of participants fulfilled the criteria.

b Trials with participants reporting symptoms suggestive of neuropathic pain (possible criteria), and diagnostic tests confirm a nerve lesion (definite criteria) but with no objective sensory signs in ≥50% of participants, were judged as probable neuropathic pain [19].

**Table 2:** Study characteristics

Study Design Recruited; completed	Group 1 (G1;) Recruited; Drop out; Age; Gender; Duration; Severity	Group 2 (G2;) Recruited; Drop out; Age; Gender; Duration; Severity	Medication duration; dose (plus rescue meds allowed)	Primary (PO)/ secondary outcome (SO) where specified	Primary pain outcome (as reported in the study) and between group *p* value	Primary disability outcome (as reported in the study)
**SNRI;** 4 RCTs, [61–63, 73] 1 cross over trial [64]. 1540 participants recruited and 1182 completed. Mean age 55.3 (51.2-60). Female 56.5%. Mean symptoms duration 8.1 yrs. (1.5-12.3yrs.) Mean severity 6 (5.1-6.8) Mean treatment duration of 11.2 weeks, (range 4-14 [73] weeks). Daily dose ranging from 20mg to 120mg [62]						
Konno et al [73] Randomised double blind placebo-controlled trial Recruited *n=*458 Completed *n*=409	*n*=232 Drop out *n*=23 Age 60 (13.2) Female 50% Duration (yr.) 9.8 (10.1) Severity (BPI 0-10) 5.1 (1.1)	*n*=226 Drop out *n*=26 Age 57.8 (13.7) Female 54% Duration (yr.) 10.3 (10.6) Severity (BPI 0-10) 5.1 (1.0)	G1; **14 weeks duloxetine** Weeks; 1; 20 mg OD 2; 40mg 3-14; 60mg G2; **14 week placebo**	PO; BPI average pain score change at 14 weeks SO; BPI worse, least and pain right now. PGI-I, CGI-severity, RMDQ, LBP specific QOL, SF36, EQ5D, WPAI	Mean (SE) change Score, BPI average pain, 14 weeks; G1; -2.43 (0.11) G2; -1.96 (0.11) (*p*=0.0026)	Mean (SE) change score RMDQ (24), 14 weeks G1; -3.86 (0.22) G2;-3.23 (0.22) (*p*=0.0439)
Schukro 2016 [64] Randomised placebo controlled double blind crossover trial Recruited *n=* 41 Completed *n=* 21	*n*=41 Age 57.9 (13.4) Female 51% Duration (mo.) 18 (6-70 range) Severity VAS (0-10) 6.8 (1.5)	*n=* 41 (cross over trial) 1^st^ phase *n*=16/18 in G1;/G2; 2^nd^ phase *n*=15/11 in G1;/2	G1; **4 weeks duloxetine** Weeks; 1; 30-60mg 2-4; 120mg 2-week washout then cross over G2; **4 weeks placebo** (rescue meds; up to 3000mg metamizole or 600mg tramadol if required)	**PO; Week 4 mean** **VAS from twice** **daily self-reported VAS.** SO; painDETECT questionnaire SF36	Mean (SD) final value VAS, 4 weeks; G1; 4.1 (2.9), G2; 6.0 (2.7) (*p*=0.001)	Mean (SD) final value SF-36 Physical composite score, 4 week; G1; 36 (10.9) G2; 31.3 (9.3) (*p =* 0.007)
Skljarevski 2009 [62] Double blind parallel randomised placebo controlled trial Recruited *n*=404 Completed *n*=267	(a) *n*=59 Drop out *n*=16 Age 52.9 (12.8) Female 22% Duration (yr.) 12.5 (11.7) Severity (BPI 0-10) 6.4 (1.4) (b) *n*=116 Drop out *n*=36 Age 53.3 (14.7) Female 57.8% Duration (yr.) 10.5 (11.1) Severity (BPI 0-10) 6.2 (1.4) (c) *n*=112 Drop out *n*=50 Age 54.9 (14.8) Female 58% Duration (yr.) 13.9 (13) Severity (BPI 0-10) 6.1 (1.5)	*n*=117 Drop out *n*=35 Age 54 (13.5) Female 54.7% Duration (yr.) 10.3 (9.5) Severity (BPI 0-10) 6.2 (1.3)	G1; **13 weeks duloxetine** (a) 20mg, (b) 60mg (c) 120mg plus 2-week taper phase G2; **13 weeks placebo** (add on meds; NSAIDs and other rescue analgesia allowed for <3 consecutive days <20 days in total, if required)	**PO; Pain severity (weekly mean of 24 hr pain ratings) in 60mg duloxetine group at 13 weeks.** SO; PGI-I, RMDQ, 24-hour average pain, night and worst pain ratings on BPI-S/BPI-I, CGI-S ; Athen’s insomnia scale, SF-36, EQ5D, Beck depression inventory II; HAD.	Mean (SE) change score BPI average pain, 13 weeks; G1;a average pain -1.79 (0.30) G1;b average pain -2.50 (0.22) G1;c average pain -2.45 (0.22) G2; average pain -1.87 (0.22) (*p*>0.05)	Mean (SD) RMDQ-24 change score extracted from figure (SD borrowed from 2010a[61]) G1;a -2.3 (4.13) G1;b -2.7 (4.13) G1;c -2.9 (4.13) G2; -1.3 (4.28)
Skljarevski 2010 [63] Randomised double blind parallel group placebo-controlled trials Recruited *n*=236 Completed *n*=182	*n*=115 Drop out *n*=31 Age 51.8 (14.9) Female 61.7% Duration (yr.) 8.8 (8.8) Severity (BPI 0-10) 5.9(1.4)	*n*=121 Drop out *n*=23 Age 51.2 (13.5) Female 60.3% Duration (yr.) 9.5 (8.6) Severity (BPI 0-10) 6.0 (1.7)	G1; **13 weeks duloxetine** Weeks; 1; 30mg OD 2-6; 60 mg OD 7-13; non-responders 120 mg, responders 60mg OD G2; **13 week placebo** (add on meds; episodic use of short acting analgesic if required)	**PO; BPI 24-hour average pain rating reduction at 13 weeks.** **PGI-I, RMDQ,** SO; 24-hour average pain, night pain and worst pain scores, CGI-S Athens insomnia scale, SF-36, EQ5D, WPAI, Beck depression inventory, HADS-A	Mean (p value) change score BPI average pain 13 weeks; G1;-2.32 G2; -1.50 (*p*=0.004)	Mean (p value) change score RMDQ-24, 13 weeks; G1; -3.60; G2; -1.93 (*p =* 0.009)
Skljarevski 2010a [61] Randomised double blind placebo-controlled study Recruited *n*=401 Completed *n*=303	*n*=198 Drop out *n*=51 Age 54.9 (13.7) Female 59.6% Duration (yr.) 8.3 (8.2) Severity (BPI 0-10) 5.8 (1.4)	*n*=203 Drop out *n*=47 Age 53.4 (14.2) Female 63.1% Duration (yr.) 8.7 (9.0) Severity BPI (0-10) 5.8 (1.4)	G1; **12 weeks duloxetine** 60mg OD G2; **12 week placebo** (add on meds; episodic use of short acting analgesic if required)	**PO; Reduction in BPI 24-hour average pain rating at 12 weeks.** SO; PGI-I, RMDQ 24, BPI -severity, BPI-interference, 30% and 50% sustained response, CGI-severity, profile of mood (POMS brief form), WPAI, SF-36, EQ5D and AEs	Mean (SE) change score, BPI average pain, 12 weeks; G1; -2.25 (0.15) G2; -1.65 (0.15) (*p*=0.002)	Mean (SE) change score, RMDQ 24, 12 weeks; G1; -2.69 (0.31) G2; -2.22 (0.32) (*p*=0.255)
**Pregabalin**; 7 RCTs [65, 67, 70, 74, 77, 82, 83] one cross over trial [71]. 1143 participants were recruited and 1029 completed. Mean age 54.5 (43-71): 51% female: Mean symptom duration 3.4 yrs.(0.17-11.9yrs.): Mean severity 6.2 (3.2-8.3): Mean treatment duration 5 weeks (range 14 days [71, 82] – 8 weeks [70, 83]) and a daily dose starting from 45mg [82] to 600mg [70] (table 3).						
Study Design Recruited; completed	Group 1 (G1;) Recruited; Drop out; Age; Gender; Duration; Severity	Group 2 (G2;) Recruited; Drop out; Age; Gender; Duration; Severity	Medication **duration;** dose (plus rescue meds allowed)	**Primary (PO)**/ secondary outcome (SO) where specified	Primary pain outcome (as reported in study)	Primary disability outcome (as reported in study)
Baron 2010 [77] Randomised placebo controlled withdrawal trial Recruited *n*=217 Completed n-187 Double blind phase	*n=* 110 Drop out *n*=12 Age 52.5 (11.1) Female 49.1% Duration Not reported Severity; Pain score (0-10) 6.36 (1.51)	*n*=107 Drop out *n*=18 Age 52.6 (12.8) Female 55.1% Duration; Not reported Severity 6.39 (1.45)	G1; **5 week optimum dose pregabalin** (dose identified in single blind phase) G2; **5 weeks placebo** (add on meds; stable dose of analgesics excluding anti-epileptics and opioids if required)	**PO; Time to loss of therapeutic response** measured by; Increase in Daily Pain Rating Scale =>1 or use of rescue medication; plus weekly mean pain score at end of double blind phase which had returned to within 30% of weekly mean pain score at screening) *DSI, HADS, PGIC, MOS-S, PTTS,* *RMDQ, WPAI*	LOR G1; 27.8% G2; 28.0% Hazard Ratio = 0.874 [95% CI: 0.520, 1.470] Mean (p value) change score VAS 5 weeks; G1; -0.16 G2; 0.05 (*p =* 0.332)	No difference in RMDQ between groups during the double blind phase of the trial (*p*=>0.05)
Chen 2022 [82] Randomised controlled trial Recruited *n*=142 Completed *n*=142	(Celecoxib plus pregabalin group) *n*=71 Drop outs *n*=0 Age 45.7 (7.3) Female = 49% Duration 11.9 m. (1.58) Severity VAS 8.24 (1.42)	(Celecoxib group) *n*=71 Drop outs *n*=0 Age 47.6 (6.45) Female = 46% Duration 11.4 m. (1.61) Severity VAS 8.3 (1.37)	G1; **2 weeks Celcoxib plus pregabalin;** Celecoxib 200mg BD plus Pregabalin Day 1-10 15mg TDS Day 10-14 75mg TDS G2; **2 weeks of Celecoxib** 200mg BD	Primary outcome not specified but measured VAS at 2, 7 and 14 days, plus ODI at 7 and 14 days	Mean (SD) final value score; VAS at day 14; G1; 1.55 (0.37) G2; 2.77 (0.52) (*p*=0.012)	Mean (SD) final value score; ODI at day 14; G1;16.18 (3.62) G2; 23.59 (3.78) (*p*=0.005)
Kim 2016 [74] Prospective double blind, double dummy randomized controlled trial (3 arms) Recruited *n*=122 (into Rx and control group) Completed *n*=83	*n*=61 Drop out *n*=18 Age 62 (8.7) Female 62.3% Duration m. 5.02 (14.64) Severity VAS (0-10) 6.7 (1.7)	*n*=61 Drop out *n*=21 Age 62.9 (9) Female 70.5% Duration m. 7.04 (17.43) Severity VAS (0-10) 6.2 (1.7)	G1; **8 weeks limaprost plus pregabalin** Limaprost 5ug TDS, pregabalin 75 mg TDS G2; **Limaprost group** 5ug TDS	**Baseline-adjusted** **ODI score at 8 weeks.** VAS scores for leg pain, the European Quality of Life-5 dimensions (EQ-5D), and initial claudication distance (ICD).	Mean (95% CI) baseline adjusted VAS change score 4 weeks; G1; 5.0 (4.6–5.5) G2; 5.0 (4.5–5.4) 8 weeks G1; 4.2 (3.8-4.7) G2; 4.0 (3.5–4.5)	Mean baseline adjusted ODI change score 4 weeks; G1; 32.1 (95% CI 29.5-34.6) G2; 33.9 (95% CI 31.3-36.5) 8 weeks G1; 29.0 (26.3–31.7) G2; 29.9 (95% CI 27.3-32.6)
Markman 2015 [71] Double blind randomised active placebo controlled 2 period cross over trial *n*=29 randomised *n*=26 completed	Pregabalin first group *n*=14 Drop out *n*=2 Age 71.1 (7.9) Female 29% Duration 3-6 months 2 (15%) >12 months 11 (84%) Severity BPI (SF 0-10) 4.6 (1.4)	Diphendydramine first group *n*=15 Drop out *n*=1 Age 69 (8.7) Female 33% Duration 3-6 months 1 (7%) >12 months 14 (93) Severity BPI (SF 0-10) 4.6 (1.9)	G1; **14 days Pregabalin** Days; 1-4; 75mg BD 4-11; 150mg BD as tolerated 11-14 ;75mg BD G2; **14 days active placebo** (Diphenhydramine 6.25mg up to 12.5mg) (add on meds; other analgesics if required, excluding gabapentin)	**PO; Time to first reported pain of moderate intensity during treadmill walking at 14 days** SO; ODI Modified BPI-SF Swiss spinal stenosis questionnaire RMDQ	No sig difference in time to first moderate pain intensity (median difference) 1.08 (95% CI -2.25-0.08) Mean (SE) NRS (0- 10) final value at end of Treadmill test 2 weeks; pregabalin phase; 7.22 (0.36) Placebo phase 6.97 (0.36)	Mean (SE) final value RMDQ 24 2 weeks pregabalin phase 12.98 (0.92) Placebo phase 11.48 (0.92) (*p*= 0.01)
Mathieson 2017 [70] Randomised double blind placebo-controlled trial *n*=209 recruited *n*=178 completed	*n*=108 Excluded *n*=2 Drop out *n*=15 Age 52.4 (17.2) Female 62.3% Duration of spine related leg pain (days) 63.7 (75.9) Severity NRS (0-10) of leg pain 6.3 (1.8) Back pain 5.9 (2.8)	*n*=101 Drop out *n*=14 Age 55.2 (16) Female 48.5% Duration of spine related leg pain (days) 62.4 days (78.7) Severity NRS (0-10) of leg pain 6.1 (1.9) NRS Back pain 5.1 (3) NRS	G1; **8 weeks of pregabalin** Weeks; 1-2; 75mg BD 3-7; 300mg BD 8; taper G2; 8 **week of placebo** (Add on meds; other analgesic medication allowed if required, except neuropathic pain medication)	**PO; Average spine related leg pain intensity NRS at 8 weeks and 52 weeks.** SO; RMDQ, back pain intensity, global perceived effect, quality of life on short form health survey, painDETECT score	Mean (SD) NRS (0-10) final value leg pain at 8 weeks; G1; 3.7 (2.97) G2; 3.1 (2.6) 26 weeks; G1; 3.1 (3.0) G2; 3.2 (2.8) 52 weeks; G1; 3.4 (3.2) G2; 3.0 (2.6)	Mean (SD) RMDQ 23 final value at 8 weeks; G1; 9.1 (7.4) G2; 8.5 (7.1) 26 weeks G1; 7.4 (7.4) G2; 8.8 (7.5) 52 weeks G1; 8.2 (7.6) G2; 7.4 (7.2)
Nakashima 2019 [67] Prospective randomised controlled trial Recruited *N=* 60, Completed *n*=56	*n*=30 Drop out *n*=4 Age 48.1 (12.8) Duration NR but inclusion criteria was acute pain (2/7-2/52) Female 46.2% Severity VAS (0- 100) 74.3 (12.3)	*n*=30 Drop out *n*=0 Age 53.7 (15.5) Duration NR but inclusion criteria was acute pain (2/7-2/52) Female 43.3% Severity VAS (0- 100) 75.7 (15.7)	G1; **4 weeks of NSAID plus pregabalin** Weeks; 0-2 NSAID 180mg, OD 2-6 NSAID 180mg plus pregabalin 150mg OD G2; **6 weeks of NSAID** 180mg OD (Add on meds; Stable dose of SNRIs and SSRIs allowed if required)	**PO; Spine related leg pain at 2 and 4 weeks.** SO; Sleep disturbance and patient / clinician global impression of change at 2 and 4 weeks	Mean (SD) final value VAS (0-100) at 4 weeks; G1; 20.5 (14.3) G2; 27.7 (16.1) (*p*= 0.08)	Not assessed
Pota 2012 [65] Randomised single blind placebo controlled trial Recruited *n*=45. Completed *n*=44	G1; +G2; characteristics reported together; Males 59% Male age 55(8.6), Male duration mo. LBP 15.6 (8.8) Female 50% Female age 56 (8.2) Female duration mo. 14.9 (8.6) G1; at randomisation severity VAS (0- 100) 35.2 (10)	G2; at randomisation severity VAS (0- 100) 31.6 (9.4)	G1; **3 weeks transdermal buprenorphine (TB) plus pregabalin** Weeks; 0-3 TB 35ug/h 3-6 TB 35ug/h plus pregabalin 150mg BD. G2; **3 weeks TB plus placebo** Weeks; 0-3 TB 35ug/h 3-6 TB 35ug/h plus pregabalin 150mg BD. (Add on oral paracetamol 1000mg up to TDS allowed if required)	**PO; Weekly mean VAS, at 6/52** SO; Present pain intensity and PRI scales of SF-MPQ and sleep quality assessed	Mean SD final value VAS (0-10) Week 3; G1; 5.7 (4.9) G2; 17.6 (4.2) (*p*<0.05)	Not assessed
Yeole 2022 [83] Randomised multicentre open label controlled trial Recruited *n*=319 Completed *n*=313	*n=* 160 Drop outs =2 Age 43.13 (11.59) Female 61.3% Duration not reported but inclusion >3 months pain Severity NRS (0-10) 7.26 (1.11)	*n*=159 Drop outs 4 Age 45.26 (10.55) Female 59.1% Duration as per G1; Severity NRS 7.22 (1.02)	G1; **8 weeks pregabalin plus etoricoxib** Prolonged release pregabalin 75 mg/ etoricoxib 60 mg G2; **8 weeks** **etoricoxib** 60 mg (Add on meds; paracetamol 500 mg every 6–8 h (total daily dose not exceeding 2 g) if required)	**PO; Mean change in the NRS (verbal rating) at 8 weeks.** SO; NRS change at 4 weeks, RMDQ change at 4 & 8 weeks; PGI-I, CGI-I at 4 & 8 weeks, amount of rescue medication	Mean (SD) NRS final change score at 8 weeks; G1; - 4.00 ± 1.65 G2; - 2.92 ± 1.59 (*p*=<0.0001)	RMDQ mean (SD) change score at 8 weeks; G1; - 9.28 ± 4.48 G2; - 6.78 ± 4.34 (*p*=<0.0001)
**Gabapentin;** Five RCT [57, 58, 68, 80, 81], one cross over study [69]. 383 participants were recruited and 316 completed. Mean age 47.3 (38-57.6). Female 52%, Mean symptoms duration 9.4 yrs. (5.3-17.8yrs.) Mean severity 7.9 (5.3-17.8), Mean treatment duration of 9.3 weeks (range 6 weeks [68, 69] – 4 months [58]) and a daily dose ranging from 900mg [57, 58, 81] to 3600 mg [58, 80].						
Study Design Recruited; completed	Group 1 (G1;) Recruited; Drop out; Age; Gender; Duration; Severity	Group 2 (G2;) Recruited; Drop out; Age; Gender; Duration; Severity	Medication **duration;** dose (plus rescue meds allowed)	**Primary (PO)**/ secondary outcome (SO) where specified	Primary pain outcome (as reported in study)	Primary disability outcome (as reported in study)
Atkinson 2016 [80] Double blind Placebo randomised controlled trial Recruited *n=* 108 Completed *n*=72	*n*=55 Drop out *n*=19 Age 57.58 yrs. (8.84) Female 19% Duration yrs 17.16 (15.12) Severity NRS (0-10) 5.7	*n*=53 Drop out *n*=17 Age 54.62 yrs. (11.38) Female; 24% Duration yrs. 17.79 (12.80) Severity NRS (0-10) 5.8	G1; **12 weeks Gabapentin**; Weeks; 1; 300mg TDS 2; 600mg TDS 3; 800mg TDS 4-12; 1200 TDS. G2; **12 weeks inert placebo** Add on meds; NSAIDs allowed	**PO; DDS at 12** **weeks** SO; ODI Blinded CGIC NRS 0-10 Patient rating Better, same, worse	Mean (p value) NRS final value at 12 weeks G1; 3.51 (*p*=0.234) G2; 4.1 (*p*=0.234) No sig difference in DDS pain intensity (*p =* 0.423) or pain un-pleasantness (*p =* 0.523).	Mean (SD) not reported but no sig difference between groups in ODI (*p =* 0.804).
Haddadi 2016 [81] Randomised controlled trial Recruited *n*=60 Completed *n*=57 Groups not included in numbers; G3 Nasal calcitonin	*n*=30; Drop out *n*=3 Age 50.59 (6.83) Gender not reported Duration not reported Severity not reported	*n*=30; Drop out *n*=0 Age 51 (6.33) Gender not reported Duration not reported Severity not reported	G1; **8 weeks** **gabapentin** 300mg TDS G2; **8 weeks Inert placebo** Add on meds; All groups took NSAID at night	Primary outcome not specified but ODI at 8 and 12 weeks recorded plus patient satisfaction.	Not measured	Mean (SD) final value ODI 8 weeks G1; 30.42 (16.07) G2; 36 (14.09) 12 weeks G1; 32 (16.08) G2; 38 (22.09)
McCleane 2000 [69] Double blind placebo controlled cross over study Recruited *n*=30 Completed *n*=24	*n*=30 Drop out *n*=not reported Demographics for both groups; Age 42.4 (14.6) Duration 105.5 mo. (97.2) Females 54.2% Severity; not reported	*n*=30 Drop out *n*=not reported Mean pain VAS week 1 G2; group 7.52 (1.94)	G1; **6 weeks gabapentin** Weeks; 1; 300mg OD 2-6; increase dose by 300mg weekly up to total dose of 15mg/kg-1. 1 week washout before cross over. G2; **6 weeks placebo** Add on meds; Stable dose of NSAIDs and paracetamol /codeine if required.	Primary outcome not specified but recorded average daily pain and self-reported ability to flex spine from 1-6 weeks	Mean (SD) final value VAS (0-10) Week 6 G1; 6.39 (2.50)* G2; 7.13 (2.34) (baseline to end point*p<0.05)	Not measured
McCleane 2001 [68] Randomised double bind placebo controlled study Recruited *n*=80 Completed *n*=65	*n*=40 Drop out *n*=9 Age 41.3 (13.1) Duration mo. 63.1 (45.3) Female 48.4% Severity neuropathic painS (0-10) leg pain 6.37 (2.27)	*n*=40, Drop out *n*=6 Age 47.8 (11.7) Duration mo 74.5 (82) Female 61.8% Severity neuropathic painS (0-10) leg pain 6.57 (2.32)	G1; **6 weeks gabapentin** Weeks; 1; 300mg OD 2; 300mg BD 3; 300mg TDS 4; 300mg QDS 4-6; Max dose maintained G2; **6 weeks** **placebo**	Primary outcome not specified but recorded average NRS daily back pain, daily spine related leg pain and daily ability to flex spine reported by subjects at baseline and 8 weeks	Mean (SD) final value VAS (0-10) 8 weeks leg pain; G1; 5.92 (2.61) G2; 6.33 (2.39) (*p*=>0.05)	Not measured
Yaksi 2007 [58] Randomised controlled study Recruited *n*=55. Completed *n*=55	*n*=28 Drop out *n*=0 Age 50.7 (9.6) Duration NR Female 79% Severity VAS (0-10) 7.0 (1.5)	*n*=27 Drop out *n*=0 Age 50.9 (10.5) Duration NR Female 56% Severity VAS 6.7 (1.2)	G1; **4 months of gabapentin** 300mg TDS. Increasing by 300mg each week up to max 2400mg for 4 months plus usual care (as per G2;) G2; **4 months of usual care** (physiotherapy lumbosacral corset and NSAIDs)	Primary outcome not specified but recorded walking distance, motor and sensory deficit and back and spine related leg pain on movement at baseline and 4 months	Mean (SD) final value NRS (0-10) 2 months; G1; at 4.3 (2.1) G2; 5.0 (1.8) (*p*=0.119) 4 months G1; 2.9 (2.6) G2; 4.7 (2.2) (*p*=0.006)	Not measured
Yildirim 2003 [57] Randomized placebo-controlled study Recruited *n*=50. Completed *n*=43	*n*=25 Drop out *n*=2 Age 38 (7.4) Female 60% Duration mo. 69.3 (56) Severity pain at rest (0-3 scale) 1.6 (0.94)	*n*=25 Drop out *n*=5 (inefficacy) Female 68% Age 40.5 (10.5) Duration mo. 67.7 (63.4) Severity score for pain at rest (0-3 scale) 1.68 (0.67)	G1; **8 week** **gabapentin** from 300 mg up to a total of 1200 mg TDS. Dose increased every 3 days depending upon AE G2; **8 weeks of** **placebo** Add on meds; nil allowed	Primary outcome not specified but recorded; location of pain, severity of pain on 0-3 scale, limitation of spinal flexion, SLR, reflexes, sensory change and muscle strength at 8 weeks	8 weeks pain score (scale 0-3); G1; 0.56 (0.58) G2; 1.36+0.59 (*p*=<0.001)	Not tested
**TCA**; 553 participants were recruited and 412 completed. Two RCTs used desipramine [76, 79] with a serum dose targeted at 12-150ng/mL [79]; One nortriptyline RCT[78] and one nortriptyline cross over trial [75] with a dose ranging from 50-150mg [78]; Four amitriptyline RCTs [59, 60, 66, 72] with a dose ranging from 10 [72]-150 [66]-mg. Mean duration of 9.4 weeks (range 13 days [72] to 6 months [60]. Mean age 50.4 (45.8-57.9): Female 31.6%: Duration 9.8 yrs. (0.2-16yrs.): Mean severity 5.3 (3.9-7.4).						
Study Design Recruited; completed	Group 1 (G1;) Recruited; Drop out; Age; Gender; Duration; Severity	Group 2 (G2;) Recruited; Drop out; Age; Gender; Duration; Severity	Medication **duration;** dose (plus rescue meds allowed)	**Primary (PO)**/ secondary outcome (SO) where specified	Primary pain outcome (as reported in study)	Primary disability outcome (as reported in study)
Atkinson 1998 [78] Randomised double blind placebo controlled trial Recruited *n*=78 Completed *n*=57	*n*=38 Drop out *n*=10 Age 45.79 (10.59) Female 0% Duration yrs. 16.04 (11.25) Severity DDS (0-20) 12.3 (5.2)	*n*=40 Drop out *n=* 11 Age 47.13 (10.65) Female 0% Duration 13.58 yrs. (12.94) Severity DDS (0-20) 9.4 (3.5)	G1; **8-week** **nortriptyline** Starting at 25mg and increased every 3-4 days to within 50- 150mg OD G2; **8 weeks** **placebo** Add on meds; non- opioids e.g. NSAIDs if required	**PO; DDS at 8** **weeks** SO; Sickness impact profile, quality of wellbeing scale, Beck depression inventory, Spielberger state anxiety inventory, Hamilton anxiety depression rating scales and CGIC	Mean SD change score of (DDS 0-20) at 8 weeks; G1; -2.59 (4) G2; -0.91 (3.43) (*p =* 0.050)	Mean (SD) change score of SIP (sickness and disability impact profile score 0-100) at 8 weeks; G1; -2.85 ± 4.14 G2; -1.05 ± 3.82 (*p =* 0.055)
Atkinson 2007 [79] Double-blind, prospective, controlled- concentration randomized study Recruited *n*=78 Completed *n*=52 Groups not included in numbers; G3 Fluoxetine	*n*=52 (3 groups) Low dose *n*=17. Medium dose *n*=17. High dose *n*=18 Drop out *n*=22 Demographic provided for all groups combined; Age 46.4 (10.2) Female 39% Duration yrs. 10 Severity (DDS 0-20) 9.4 (4)	*n*=26 Drop out *n*=4	G1; **12 weeks** **desipramine** 4-week escalation phase, 8-week maintenance phase. Low medium or high concentration of desipramine (50, 110, 150 ng/mL) G2; **12 weeks placebo** (Benztropine mesylate) Add on meds; non-opioids i.e. NSAIDs	**PO; DDS at 12** **weeks**; SO; RMDQ and CGIC	Mean (SD) final value DDS score (0- 20) at 12 weeks; G1; 6.0 (4.1) G2; 6.8 (3.8) (*p =* 0.560)	Mean (SD) final value RMDQ 24 at 12 weeks; G1; 2.3 (2.5) G2; 4.1 (2.5) (*p =* 0.05)
Gould 2020 [76] Double-blind, 12- week, 4-arm, parallel groups controlled clinical trial In G1;/G2; group; Recruited *n*=71 Completed *n*=51 Groups not included in numbers; G3 CBT and placebo G4 Desipramine and CBT	*n*=38, Drop out *n*=11 Age 56.5 (11.7) Female 23% Duration not reported Severity (DDS 0-20) 9.97 (5.05)	*n*=33 Drop out *n*=9 Age 57.9 (10.9) Male 90.3% Duration not reported Severity (DDS 0-20) 11.96 (4.63)	G1; **12 weeks** **desipramine** (aiming for serum conc. of 12-65ng/ml) G2; **12 weeks** **placebo** (Benztropine mesylate, 0.125 mg) Add on meds; NSAIDs if required	**PO; DDS at 12** **weeks** SO; RMDQ	Mean (SD) final value DDS score (0- 20) at 12 weeks; G1; 7.55 (5.63) G2; 8.74 (5.55) (*p*=0.80)	Mean (SD) final value RMDQ 24 at 12 weeks; G1; 7.65 (5.99) G2; 9.50 (5.15) (*p*=0.94)
Khoromi 2007 [75] Single centre four- period, crossover, randomized trial comparing four treatments Recruited *n*=61 Completed *n*=28 (Cross overs not included in analysis G3 morphine G4 morphine and nortriptyline)	*n*=28 completed all 4 trial arms All participants Age median 53 (range 19-65) F/M 25/30 Duration median yrs 5 (0.3-37) Severity VAS (0-10) 4.9 (2.43)	*n*=28 completed all 4 trial arms	G1; **7 weeks** **nortriptyline** starting at 25mg and increasing weekly as tolerated to mean dose of 84 +/− 24.44 (SD) mg/day G2; **7 weeks** **placebo** **(**benztropine (0.25- 1mg)	**PO; Leg pain VAS** **at 7 weeks** SO; Global pain relief, ODI, Beck depression inventory, SF36	Mean (SD) final value score VAS (0- 10) at 7 weeks; G1; 3.0 (2.7) G2; 3.7 (2.7) (*p*>0.05)	Mean (SD) final value score of ODI (0-100) at 7 weeks; G1; 27.5 (16.7) G2; 30.5 (15.9) (*p*>0.05)
Kurniawati 2020 [72] Double blind randomised controlled trial Recruited *n*=63 Completed *n*=63	*n*=33 Drop out *n*=0 Age 42 Median (Range 20-49) Female 79% Severity VAS (0-10) median 5 (range 4.2- 8.5) Duration Not reported	*n*=30 Drop out *n*=0 Age 37.5 median (20-49) Severity VAS (0-10) 5.2 (4.18.2) Female 70% Duration Not reported	G1; **13 days of** **amitriptyline** 10mg plus acetaminophen (3×500mg) G2;=**13 days of placebo** plus acetaminophen	**PO; VAS at day 13**		Not measured
					Mean (SD) change score of VAS (0-10) at 13 days; G1; -3.51 (2.01) G2; 4.08 (2.05) (*p*=0.28)	
Pheasant 1983 [66] Randomised blind crossover study Recruited *n*=16 Completed *n*=9	*n*=16 (6 Rx first) All participants Age average 47.2 (range 22-68) Female 67% Duration 9.9 yrs. (1- 37) Severity not reported	*n*=16 (10 placebo first)	G1; **6 weeks of** **amitriptyline** 50mg up to TDS, G2; **6 weeks of** **placebo** atropine 0.2mg (similar side effects but not an analgesic) Add on meds; paracetamol or aspirin with or without codeine as required	**PO; Functional** **evaluation rating** **and activity** **questionnaire, plus** **record of additional** **medication used**	Not measured	Not measured
Urquhart 2018 [60] Double blind randomised clinical trial (2 arm parallel group superiority design) Recruited *n*=146 Completed *n*=118	*n*=72 Drop out 11 Age 53.5 (14.2) Female 39% Duration yr. 13.3 (12.6) Severity (100mm VAS) 39.8 (20.5)	*n*=74 Drop out 17 Age 56 (13.2) Female 38% Duration yr. 15.2 (13.2) Severity (0-100) 43.2 (21)	G1; **6 months of** **amitriptyline** 25mg OD G2; **6 months of** **placebo** 1mg benztropine mesylate OD	**PO; VAS and DDS** **at 6 months.** SO; RMDQ and SF health and labour Questionnaire	Mean (SE) final value VAS (0-100) at 3-month; G1; 32.4 (2.1) G2; 30 (2.7) (*p*=0.91) 6 month; G1; 28.9 (2.6) G2; 37.1 (3.2) (*p*=0.09)	Mean (SE) RMDQ (0-23) final value at; 3-month; G1; 4.5 (0.5) G2; 6.5 (0.5) (*p*=0.001) 6 month; G1; 4.7 (0.5) G2; 5.9 (0.6) (*p*=0.18)
Urquhart post hoc analysis 2021 [84] As above – post hoc analysis	G1;a neuropathic	G2;a neuropathic	As above As above	**As above**	Mean (SE) final	Mean (SE) final
	pai*n*=16 G1;b non- neuropathic pain *n*=51	pain =20 G2;b non- neuropathic pain *n*=50			value VAS (0-100)	value RMDQ (0-24)
					at	at 3 months;
					3-month;	G1;a 6.4 (1.1)
					G1;a 39.7 (6.4)	G1;b 4.1 (0.6)
					G1;b 26.9 (3.4)	G2;a 8.5 (1.1)
					G2;a 34.4 (4.6) G2;b 31.7 (3.4) 6 months; G1;a 26.0 (5.3) G1;b 29.1 (3.3) G2;a 46.2 (5.7) G2;b 32.9 (4.0) (Between group measures adjusted for baseline)	G2;b 5.9 (0.7)
						6 month;
						G1;a 4.3 (1.0)
						G1;b 4.7 (0.7) G2;a 9.6 (1.1) G2;b 4.7 (0.8) (Between group measures adjusted for baseline) .
Vanelderen 2015 [59] Randomized, double-blind, placebo-controlled clinical trial Recruited *n*=40 Completed *n*=34 Excluded from analysis; G3 =minocycline group	*n*=20 Drop out *n*=3 Age 50 (3 SEM) Female 35% Duration mo. 3.2 (0.6SEM) Severity NRS (0-10) 6.9 (0.4 SEM)	*n*=20 Drop outs *n*=3 Age 51 (3) M/F 9 (45%) 11 (55%) Duration 2.8 months (0.4) Severity NRS 7.4 (0.3)	G1; 1**4 days** **amitriptyline** 25mg G2; **14 days placebo** Add on meds; Paracetamol or NSAIDs drugs if stable dose 1 week before enrolment. Plus 50 mg tramadol with a maximum of three intakes daily as required	**PO; Leg pain NRS** **0-10 on days 7 and** **14;** SO; DN4 score and rescue medication used	Data extracted from figure using plot digitizer, mean (SE) final value NRS (0- 10) G1; 4.8 (0.6) G2; 7 (0.6)	Not measured
Results reported as mean and SD unless stated otherwise				NPRS = numeric pain rating scale		

BD = twice daily dose of medication BPI-I = Brief pain inventory interference BPI-S = Brief pain inventory severity BPI-SF = Brief pain inventory, short form CGI- S = Clinical global impression of severity CGI-I =clinicians global impression of improvement CGIC = Clinician global impression of change DDS = Descriptor differential scale (verbal descriptors of pain intensity correspond to pain magnitude) DN4= Douleur Neuropathique (neuropathic pain screening tool) DSI – daily sleep interference EQ5D = EuroQol 5D questionnaire G1; = Group one (treatment group) G2; = Group two (control group) HADS = hospital anxiety and depression scale HADS-A = hospital anxiety and depression scale – anxiety scale ITT = intention to treat analysis MOS- S = Medical outcome study – sleep scale NRS Numeric rating scale of pain OD = once daily dose of medication ODI Oswestry disability index painDETECT = neuropathic pain screening tool PGI-I patient global impression of improvement PGIC = Patient global impression of change PRI = Pain rating index PTSS =pain treatment satisfaction scale QDS = four times daily dose of medication QOL = Quality of life RMDQ = Roland Morris Disability questionnaire SF-MPQ = short form McGill Pain Questionnaire SF36 = Short form 36 questionnaire SLR = straight leg raise TDS = three times daily dose of medication VAS = visual analogue scale WPAI = Work productivity and activity impairment questionnaire

**Table 3:** Judgement of certainty of neuropathic pain based on NeuPSIG grading criteria[31]

Study ID	Exclusions of neuropathic pain	Descriptors or screening tools of neuropathic pain	Bedside neurological examination or Quantitative sensory testing (QST)	Confirmatory diagnostic tests including imaging or neurophysiology	Judgement of neuropathic pain (Unclear, Unlikely, Possible, Probable or Definite)
**SNRI**					
Konno 2016 [73]	Exclusion criteria; participants with signs and symptoms of radiculopathy were excluded. Only participants with LBP were included	None	None	None	Unlikely
Schukro 2016 [64]	None	Inclusion criteria; Radicular pain described as burning, tingling pain extending below the knee and traveling along the anatomic distribution of a lumbar nerve root and painDETECT questionnaire score of >12 required for inclusion	None	None	Possible; due to minimum score on painDETECT [86] and pain location
Skljarevski 2009 [62]	Exclusion criteria; participants with clinical or radiographic evidence of radicular compression or spinal stenosis. Excluded participants with referral of pain to distal extremity (Quebec task force classification grade III)	None	None	None	Unlikely
Skljarevski 2010 [63]	Exclusion criteria; participants with radicular signs and symptoms were excluded. Excluded participants with referral of pain to distal extremity (Quebec task force classification grade III). Participants with clinical or radiographic / EMG signs of radicular compression excluded	None	None	None	Unlikely
Skljarevski 2010a [61]	Exclusion criteria; participants with clinical or radiographic evidence of radicular compression or spinal stenosis. Excluded participants with referral of pain to distal extremity (Quebec task force classification grade III)	None	None	None	Unlikely
**Pregabalin**					
Baron 2010 [77]	None	Inclusion criteria; pain radiating to calf or foot consistent with L5 or S1 nerve root. Pain in leg had to be greater than pain in back. Participants reported to have lumbosacral radiculopathy but no details on how this was diagnosed	Inclusion criteria required co-localised area of sensory change or muscle weakness in the area of pain for all participants but baseline measures of sensory loss versus weakness were not reported	None reported	Possible; Muscle weakness is not included within the NeuPSIG grading criteria of probable neuropathic pain [19]. As it is unclear what proportion of participants had sensory loss compared to muscle weakness, this trial could only be graded as possible neuropathic pain.
Chen 2022 [82]	None	Inclusion criteria; Reported all participants diagnosed with lumbar disc herniation, but no detail provided of how this was diagnosed	None	None	Unclear
Kim 2016 [74]	None	Inclusion criteria; Lumbar spinal stenosis diagnosed if one or more symptoms present: walking intolerance because of neurogenic claudication (defined as pain, numbness, or tingling in the legs occurring within 15 minutes of treadmill walking); a VAS score of more than 3 for pain, numbness, or tingling sensation in the buttocks and lower extremities;	Inclusion criteria mentions motor weakness and bladder /bowel dysfunction but no information on if or how this was assessed and no mention of bedside sensory testing	None	Possible; inclusion criteria included symptoms suggestive of neuropathic pain
Markman 2015 [71]	None	Inclusion criteria; Neurogenic claudication symptoms (buttock or leg pain), but no detail provided on location of pain in subjects at baseline. Pain required to be mild at rest (less than or equal to 3) and to increase on treadmill walking within 15 mins (equal or more than 4)	None	Radiographic evidence of lumbar spinal stenosis required for inclusion. Unclear whether this was MRI or x-ray Lumbar spine x-ray is not sensitive to nerve involvement	Unclear; Despite claudication symptoms and radiographic evidence of spinal stenosis required for inclusion, neuropathic pain features recommended by the NeuPSIG grading criteria were not explored.[19]
Mathieson 2017 [70]	None	Inclusion criteria; Back and leg pain below the knee. painDETECT questionnaire was used to identify neuropathic pain, however this was not specified as an inclusion criteria and 45% of Participants included had =<12 on painDETECT score suggesting this subgroup was unlikely to have neuropathic pain. At baseline, 88% of G1, and 81% of G2 had dermatomal pain	Inclusion criteria; One of the following; dermatomal leg pain, myotome weakness, sensory deficit, diminished reflex. Baseline data showed; 40% of G1 and 34% of G2 had neurologic deficit (although no further detail provided) 31% of G1 and 29% of G2 had motor deficit 63% of both groups had positive SLR 44% of both groups had sensory loss	None	Possible; recorded painDETECT scores at baseline, and bedside examination including sensory changes, motor deficit and SLR, however none of these were specified as inclusion criteria and at baseline only 44% of participants had sensory loss on examination, meaning we could not upgrade this study from possible to probable neuropathic pain.
Yeole 2022 [83]	None	CLBP (symptoms duration at least 3 months) and at least one of the following five features on the side corresponding to leg pain were considered eligible for participation in the study: (a) sharp and shooting pain below the knee; (b) pain evoked by straight leg raising to 60° or less; (c) decreased or absent ankle reflex; (d) weakness of muscles below the knee; (e) sensory loss in L5/S1 distribution, with a pain score of at least 4 on the numeric rating scale (NRS), 70% of patients in each treatment group had sharp shooting pain below the knee	42% of each group reported sensory loss in L5/S1 distribution 44% of each group had absent ankle reflex 43%-45% had muscle weakness below the knee 51%-52% of patients felt pain with straight leg raise to 60° or less.	Lumbosacral spine MRI scans showed spine abnormalities in all the patients. 64%-67% had disc herniation 19% of each group had spinal stenosis on MRI	Probable; inclusion criteria included symptoms suggestive of neuropathic pain. MRI confirmed disc herniation in >50% of participants. However <50% of participants had sensory changes on examination.
**Gabapentin**					
Atkinson 2016 [80]	Participants with back or leg pain included, but no clear assessment of radiculopathy or radicular pain reported. Subjects with neurological signs and presumptive compression of a spinal nerve root were excluded	None	Physical examination is mentioned but no details of how it was performed or the results of the exam	None	Unlikely
Haddadi 2016 [81]	None	Inclusion criteria: Back and leg pain with paraesthesia and neurogenic claudication symptoms on walking. At baseline all patients had either bilateral or unilateral leg pain, 70% of G1 and 66% of G2 had paraesthesia.	Absent reflexes specified within the inclusion criteria but no sensory examination was performed.	MRI canal stenosis specified as inclusion criteria (diameter of less than 13mm)	Probable; inclusion criteria included symptoms suggestive of neuropathic pain and canal stenosis confirmed on MRI, however there was limited bedside examination.
McCleane 2000 [69]	Only recruited participants with back pain. Participants with symptoms of neuropathic pain were excluded (shooting pain/paraesthesia/numbness/allodyn ia)	None	None	Lumbar spine x-ray was performed but this is not sensitive to nerve involvement	Unlikely
McCleane 2001 [68]	Subjects with back and leg pain were recruited but no detail provided on the location of pain. Participants with symptoms of neuropathic pain were excluded (shooting pain/ paraesthesia/ numbness/ allodynia)	None ’	None	None	Unlikely
Nakashima 2019 [67]	Participants with muscle power 3/5 or less were excluded	Inclusion criteria; Japanese neuropathic pain screening questionnaire[87] used and only Participants with a score of 6 or more[88] were recruited. Acute leg pain from 2 days to 2 weeks.	Inclusion criteria; SLR positive Patients with various degrees of sensory disturbance were included. However no details provided on how many patients had sensory disturbance	Lateral disc herniation seen on MRI an inclusion criteria	Probable; inclusion criteria included symptoms suggestive of neuropathic pain, and disc herniation seen on MRI, but limited detail provided on sensory changes on bedside examination.
Pota 2012 [65]	None	Reports of 85% of participants having a neuropathic pain component but no explanation of how this was assessed. The majority of participants had LBP (23/44), some had gluteal pain (9/44), or lower extremity pain (7/44)	None	None	Unclear; Limited reporting of participant symptoms
Yaksi 2007 [58]	Exclusions of relevance include subjects with signs of advanced disc herniation on MRI, although no further details provided on how this was judged	Inclusion criteria; Back and leg pain and signs of neurogenic spinal stenosis such as numbness / feebleness and cramps in legs on walking / standing. Although the actual numbers of patients reporting numbness was not detailed, bedside tests and MRI were performed in all participants.	61% of Rx/ 70% of control group had sensory deficit in either L4/5 or S1 dermatomes. 17% of Rx /15% of control group had motor deficit (mostly in dorsiflexion ranging from grade 2-4/5 on oxford scale)	All patient had evidence of degenerative lumbar spinal stenosis on CT or MRI	Definite; inclusion criteria included symptoms suggestive of neuropathic pain, >50% of participants had sensory loss on examination and all patients had signs of stenosis on MRI or CT
Yildirim 2003 [57]	None	Inclusion criteria was lumbosacral radiculopathy. At baseline assessment 84% of subjects reported to have unilateral radiculopathy, and 16% bilateral. The overall mean pain distribution was to heel or ankle in both Rx and control groups. No mention of neuropathic pain descriptors such as burning or shooting pain was used.	At baseline mean testing in both treatment and control group; Sensory changes averaged 1.78 (0.42) in Rx group and 1.78 (0.58) in control, where 1 is mild change and 2 is moderate sensory loss. Given the small standard deviations[89] the majority of participants appear to have had some sensory loss. SLR, reflexes and muscle strength were also examined	Spinal MRI showed that all Participants had L4-5 and/or L5-S1 bulging and/or protrusion without significant spinal stenosis.	Definite; inclusion criteria included symptoms suggestive of neuropathic pain, >50% of participants had sensory loss on examination and all patients had signs of disc bulge on MRI
**TCAs**					
Atkinson 1998 [78]	None	Inclusion criteria; patients with back pain with and without	Numbers of patients with pain with radiation and neurological signs	No diagnostic tests reported but they do differentiate numbers of	Unlikely; given small percentage of
		radicular pain. (defined as pain, burning, or tingling discomfort extending below the knee, and traveling within the anatomic distribution of a nerve root). At baseline 16% of Rx group and 23% of control group diagnosed with radicular pain. At end point, only 6% of completers had radicular pain. At baseline 13% of Rx and 15% of control group diagnosed with painful radiculopathy. It is not clear whether the radicular pain and radiculopathy cohort were the same people	(diminished knee or ankle reflexes, or decreased vibratory or pain sensation or motor strength) was 13%	patients with pain and ’root compression’ (9%) without documenting how this was assessed	participants with positive findings on subjective assessment or bedside examination.
Atkinson 2007 [79]	Excluded spine related leg pain as a primary problem	28% leg pain not past the knee 21% reported back pain with radiation below the knee (Quebec Class III).	General physical examination done including standardized back exam using Waddell impairment index and Quebec activity related spinal disorders but no results presented	If available they observed for evidence of at least mild degenerative changes of the lumbar spine using either plain radiograph (i.e. >25% reduction of expected disc space height) or magnetic resonance imaging (i.e. disc herniation, bulging annulus, or vertebral body signal intensity changes) but no results reported	Unclear; Presented findings of disc herniations or bulges on MRI, but did not specify this as an inclusion criterion and did not report the outcome of this observation at baseline.
Gould 2020 [76]	Excluded patients with spine related leg pain – described as ‘true sciatica’	LBP patients with pain intensity of =>4/10	None	None	Unclear; Limited reporting of participant symptoms
Khoromi 2007 [75]	None	Inclusion criteria; 1; Evidence of pain in one or both buttocks or legs (for at least 5 days a week) for 3 months or greater 2; Average leg pain of at least 4/10 for the past month on a numerical scale of 0 to 10	In addition to pain location they also required at least one of the following features on the side corresponding to leg pain; a) Sharp and shooting pain below the knee; OR b) Pain evoked by SLR to 60 degrees or less OR c) Decreased or absent ankle reflexes OR d) Weakness of muscles below the knee OR e) Sensory loss in L5/S1 distribution; OR (see next column)	f) Electromyography evidence for L4, L5, or S1 root denervation OR g) Imaging (MRI, CT/myelogram) evidence of nerve root compression in the lower lumbar region; BUT; no details given on baseline pain characteristics, bedside examination or diagnostics	Possible; Despite the list of neuropathic pain inclusion criteria (pain descriptors, sensory loss and diagnostic changes on EMG or MRI) only one of these needed to be present for inclusion. Baseline characteristics of each of these symptoms were not reported therefore, the trial could only be graded as possible.
Kurniawati 2020 [72]	LBP patients only	None	None	None	Unclear; Limited reporting of participant symptoms
Pheasant 1983 [66]	Chronic LBP patients recruited. No leg pain	None	None	None	Unclear; Limited reporting of participant symptoms
Urquhart 2018 [60]	None	Patients had LBP only - no leg pain. The presence of neuropathic pain was assessed using the painDETECT questionnaire (ranging from 0 to 38), with scores of 19 or greater reflecting a high likelihood of a neuropathic component. Only 13% of Rx group and 11% of comparator group had scores higher than 19.	None	None	Unlikely; given small percentage of participants with positive painDETECT
Urquhart post hoc analysis	None	Subgroup of patients assessed as more likely to likely to have neuropathic pain including (1)	None	None	Possible; given painDETECT scores higher than ≥13[86]
(subgroup with neuropathi c pai) 2021 [84]		moderate to severe pain, defined as a VAS score ≥30; and (2) a neuropathic component, based on a painDETECT score of ≥13.			
Vanelderen 2015 [59]	None	Inclusion criteria; neuropathic* lumbar radicular pain radiating into the leg below the knee caused by disc herniation, spinal canal stenosis, or failed back surgery syndrome. *neuropathic pain determined by a validated Dutch translation of the DN4[90] questionnaire including one score for each symptoms of (burning pain sensation, cold painful sensation, electric shocks, paraesthesia, “pins and needles” sensation, numbness, itching pain sensation, hypoesthesia to touch, hypoesthesia to pinprick, and mechanical allodynia, (neuropathic pain if score of =>4)	Although DN4 included a brief physical examination, no detail provided on numbers with sensory changes on examination so unable to assess if >50% of cohort had sensory change.	Patients were included only if the level of the pathology on CT or MRI correlated with the dermatome in which they indicated their leg pain and if the leg pain was predominant over the back pain (11-point numeric rating scale [NRS] score for leg pain > NRS score for back pain).	Probable; inclusion criteria included symptoms suggestive of neuropathic pain, and all patients had signs of disc pathology on MRI or CT. However no detail provided on numbers of participants with sensory changes on DN4 examination

CLB*P* = chronic LBP G1 - group 1 (treatment group) NRS = numeric rating scale TCA = tricyclic antidepressant CT = computerised tomography G2 = group 2 (control group) SLR = straight leg raise SNRI = serotonin-norepinephrine reuptake inhibitior DN4 = douleur neuropathique 4 LB*P* = LBP QST= Quantitative sensory testing EMG = Electromyography MRI = Magnetic resonance imaging VAS = visual analogue scale

## Data Availability

All data generated or analysed during this study are included in this published article or available from the authors on request.

## Key points

- Fifty-nine percent of studies using first-line neuropathic pain medication (including tricyclic antidepressants, serotonin-norepinephrine reuptake inhibitors, or anticonvulsants) for people with low back pain or spine-related leg pain either excluded people with neuropathic pain or did not assess the presence of neuropathic pain in their participants.
- Only seven percent of studies included people with definite neuropathic pain and 15% included people with probable neuropathic pain, according to retrospective grading of the certainty of neuropathic pain using the neuropathic pain Special Interest Group [NeuPSIG] neuropathic pain grading criteria.
- Subgrouping studies based on their inclusion of people with neuropathic pain revealed that medication showed larger reductions in pain and disability at short term when people with definite or probable neuropathic pain were included in the study, compared with studies including people with possible, unclear, or unlikely neuropathic pain.
- A similar trend was seen for medium-term pain and disability outcomes; however, subgroup results were limited by the low numbers of studies including participants with definite or probable neuropathic pain.
- Interpretation of all results was limited by the low to very low certainty of evidence using the Grading of Recommendations Assessment Development and Evaluation [GRADE] criteria and the small numbers of studies including people with neuropathic pain.
