# Supplementary material for: A Meta-analysis Exploring the Efficacy of Neuropathic Pain Medication for Low Back Pain or Spine-Related Leg Pain: Is Efficacy Dependent on the Presence of Neuropathic Pain
Source: Drugs. Author manuscript; Available in PMC 2024 Nov 10. (PMC7616789; doi:10.1007/s40265-024-02085-6)
Supplement: supplementary material [file EMS199723-supplement-supplementary_material.pdf]

Online Resources

Title;

A meta-analysis exploring the efficacy of neuropathic pain medication for low back pain or spine related leg pain; Is efficacy dependent on the presence of neuropathic pain ?

Journal name; Drugs

Author names;
Corresponding author; Jennifer Ward,
Anthony Grinstead,
Amy Kemp,
Prof Paula Kersten,
Prof Annina Schmid,
Dr Colette Ridehalgh,

**Online Resource tables;**	Page number
1; PRISMA checklist	p.2-3
2; The search strategy	p.4-7
3: Exclusion Criteria	p.8-15
4; GRADE judgement criteria	p.16-17
5: GRADE judgements for outcomes	p.18-26
**Online resource figures;**	
6.1; Funnel plots for all pain outcomes	p. 27
6.2; Funnel plots for all disability outcomes	p. 28
7.1; TCA pain outcomes (at primary end point), subgroups clustered by certainty of neuropathic pain	p. 29
7.2; TCA disability outcomes (at primary end point), subgroups clustered by certainty of neuropathic pain	p. 30
8.1; Anticonvulsant pain outcomes (at primary end point), subgroups clustered by certainty of neuropathic pain	p.31
8.2; Anticonvulsant disability outcomes (at primary end point), subgroups clustered by certainty of neuropathic pain	p.32
9.1; SNRI pain outcomes (at primary end point), subgroups clustered by certainty of neuropathic pain	p.33
9.2; SNRI disability outcomes (at primary end point), subgroups clustered by certainty of neuropathic pain	p.34
10.1; Meta regression of effects of neuropathic pain on outcomes<br>10.2; Sensitivity analysis of meta-regression (removing usual care trials)	p.35

Online resource 1, PRISMA checklist

Section and Topic	Item #	Checklist item	Location where item is reported
**TITLE**			
Title	1	Identify the report as a systematic review.	Title page
**ABSTRACT**			
Abstract	2	See the PRISMA 2020 for Abstracts checklist.	Abstract
**INTRODUCTION**			
Rationale	3	Describe the rationale for the review in the context of existing knowledge.	Section 1
Objectives	4	Provide an explicit statement of the objective(s) or question(s) the review addresses.	Section 1
**METHODS**			
Eligibility criteria	5	Specify the inclusion and exclusion criteria for the review and how studies were grouped for the syntheses.	Section 2.1
Information sources	6	Specify all databases, registers, websites, organisations, reference lists and other sources searched or consulted to identify studies. Specify the date when each source was last searched or consulted.	Section 2
Search strategy	7	Present the full search strategies for all databases, registers and websites, including any filters and limits used.	Online resource 2
Selection process	8	Specify the methods used to decide whether a study met the inclusion criteria of the review, including how many reviewers screened each record and each report retrieved, whether they worked independently, and if applicable, details of automation tools used in the process.	Online resource 3
Data collection process	9	Specify the methods used to collect data from reports, including how many reviewers collected data from each report, whether they worked independently, any processes for obtaining or confirming data from study investigators, and if applicable, details of automation tools used in the process.	Section 2.1
Data items	10a	List and define all outcomes for which data were sought. Specify whether all results that were compatible with each outcome domain in each study were sought (e.g. for all measures, time points, analyses), and if not, the methods used to decide which results to collect.	Table 1, section 2.2
	10b	List and define all other variables for which data were sought (e.g. participant and intervention characteristics, funding sources). Describe any assumptions made about any missing or unclear information.	Table 1, section 2.2
Study risk of bias assessment	11	Specify the methods used to assess risk of bias in the included studies, including details of the tool(s) used, how many reviewers assessed each study and whether they worked independently, and if applicable, details of automation tools used in the process.	Section 2.4<br>Online resource 4
Effect measures	12	Specify for each outcome the effect measure(s) (e.g. risk ratio, mean difference) used in the synthesis or presentation of results.	Section 2.2
Synthesis methods	13a	Describe the processes used to decide which studies were eligible for each synthesis (e.g. tabulating the study intervention characteristics and comparing against the planned groups for each synthesis (item #5)).	Section 2.2
	13b	Describe any methods required to prepare the data for presentation or synthesis, such as handling of missing summary statistics, or data conversions.	Section 2.3
	13c	Describe any methods used to tabulate or visually display results of individual studies and syntheses.	Section 2.3
	13d	Describe any methods used to synthesize results and provide a rationale for the choice(s). If meta-analysis was performed, describe the model(s), method(s) to identify the presence and extent of statistical heterogeneity, and software package(s) used.	Section 2.3
	13e	Describe any methods used to explore possible causes of heterogeneity among study results (e.g. subgroup analysis, meta-regression).	Section 2.3.1
	13f	Describe any sensitivity analyses conducted to assess robustness of the synthesized results.	Section 2.3.1
Reporting bias assessment	14	Describe any methods used to assess risk of bias due to missing results in a synthesis (arising from reporting biases).	Section 2.3

Section and Topic	Item #	Checklist item	Location where item is reported
Certainty assessment	15	Describe any methods used to assess certainty (or confidence) in the body of evidence for an outcome.	Section 2.4<br>Online resource 4
**RESULTS**			
Study selection	16a	Describe the results of the search and selection process, from the number of records identified in the search to the number of studies included in the review, ideally using a flow diagram.	Section 3<br>Figure 1
	16b	Cite studies that might appear to meet the inclusion criteria, but which were excluded, and explain why they were excluded.	Online resource 3
Study characteristics	17	Cite each included study and present its characteristics.	Table 2<br>Table 3
Risk of bias in studies	18	Present assessments of risk of bias for each included study.	Figure 2A, Figure 2B
Results of individual studies	19	For all outcomes, present, for each study: (a) summary statistics for each group (where appropriate) and (b) an effect estimate and its precision (e.g. confidence/credible interval), ideally using structured tables or plots.	Table 2<br>Table 3
Results of syntheses	20a	For each synthesis, briefly summarise the characteristics and risk of bias among contributing studies.	Section 3.2, Figure 2A<br>Figure 2B
	20b	Present results of all statistical syntheses conducted. If meta-analysis was done, present for each the summary estimate and its precision (e.g. confidence/credible interval) and measures of statistical heterogeneity. If comparing groups, describe the direction of the effect.	Section 4, Figure 3<br>Figure 5
	20c	Present results of all investigations of possible causes of heterogeneity among study results.	Figure 4A, Figure 4B<br>Figure 6A. Figure 6B<br>Online resource 6.1-6.2
	20d	Present results of all sensitivity analyses conducted to assess the robustness of the synthesized results.	Figure 4A, Figure 4B<br>Figure 6A. Figure 6B<br>Online resource 6.1-6.2
Reporting biases	21	Present assessments of risk of bias due to missing results (arising from reporting biases) for each synthesis assessed.	Figure 2A, Figure 2B
Certainty of evidence	22	Present assessments of certainty (or confidence) in the body of evidence for each outcome assessed.	Section 3.2, Section 4<br>Figure 4A, Figure 4B<br>Figure 6A. Figure 6B<br>Supplemental figures 1.1-4.2
**DISCUSSION**			
Discussion	23a	Provide a general interpretation of the results in the context of other evidence.	Section 5
	23b	Discuss any limitations of the evidence included in the review.	Section 5
	23c	Discuss any limitations of the review processes used.	Section 5
	23d	Discuss implications of the results for practice, policy, and future research.	Section 5
**OTHER INFORMATION**			
Registration and protocol	24a	Provide registration information for the review, including register name and registration number, or state that the review was not registered.	Section 2
	24b	Indicate where the review protocol can be accessed, or state that a protocol was not prepared.	Section 2
	24c	Describe and explain any amendments to information provided at registration or in the protocol.	Section 2
Support	25	Describe sources of financial or non-financial support for the review, and the role of the funders or sponsors in the review.	Section 7
Competing interests	26	Declare any competing interests of review authors.	Section 7
Availability of data, code and other materials	27	Report which of the following are publicly available and where they can be found: template data collection forms; data extracted from included studies; data used for all analyses; analytic code; any other materials used.	Available from author

From: Page MJ, McKenzie JE, Bossuyt PM, Boutron I, Hoffmann TC, Mulrow CD, et al. The PRISMA 2020 statement: an updated guideline for reporting systematic reviews. BMJ 2021;372:n71. doi: 10.1136/bmj.n71

Online resource 2; The search strategy

2.1 ; Medline search strategy

1.	low back pain/
2.	sciatica/
3.	radiculopathy/
4.	((lumbar or lumbosacral or lumbo-sacral or back) adj5 (pain* or ache* or aching)).ti,ab.
5.	(backache* or lumbago or sciatica).ti,ab.
6.	(radiculopathy or radiculitis or radicular pain*).ti,ab.
7.	(nerve root* adj5 (pain* or avulsion or compress* or disorder* or pinch* or 3ochran* or imping* or irritat* or entrap* or trap*)).ti,ab.
8.	or/6-7
9.	(back* or lumbosacral or lumbo-sacral or lumbar).ti,ab.
10.	8 and 9
11.	or/1-5,10
12.	editorial/
13.	letter/
14.	news/
15.	exp historical article/
16.	anecdotes as topic/
17.	comment/
18.	case report/
19.	(letter or comment*).ti.
20.	or/12-19
21.	randomized controlled trial/ or random*.ti,ab.
22.	20 not 21
23.	animals/ not humans/
24.	exp animals, laboratory/
25.	exp animal experimentation/
26.	exp models, animal/
27.	exp rodentia/
28.	(rat or rats or mouse or mice).ti.
29.	or/22-28
30.	11 not 29
31.	limit 30 to 3ochran language
32.	exp Anticonvulsants/ or exp gamma-Aminobutyric Acid/ or gabapentin/ or gabapentin.mp.
33.	gaba agents.mp. or exp GABA Agents/
34.	gabapentinoids.mp.
35.	pregabalin.mp. or exp Pregabalin/
36.	lyrica.mp.
37.	3ochrane3.mp.
38.	exp Antidepressive Agents, Tricyclic/
39.	exp Antidepressive Agents/
40.	(antidepress* or anti-depress*).mp.
41.	serotonin norepinephrine reuptake inhibitor*.mp.
42.	snri.mp.
43.	exp amitriptyline/ or (amitriptyline or 3ochra or vanatrip).mp. or exp nortriptyline/ or nortriptyline.mp. or exp desipramine/ or desipramine.mp
44.	Duloxetine.mp. or exp Duloxetine Hydrochloride/ or exp venlafaxine/ or venlafaxine.mp.
45.	or/32-44
46.	randomized controlled trial.pt.
47.	controlled clinical trial.pt.
48.	randomi#ed.ab.
49.	placebo.ab.
50.	drug therapy.fs.
51.	randomly.ab.
52.	trial.ab.
53.	groups.ab.
54.	or/46-53

55.	meta-analysis/
56.	meta-analysis as topic/
57.	(meta analy* or metanaly* or metaanaly*).ti,ab.
58.	((systematic* or evidence*) adj3 (review* or overview*)).ti,ab.
59.	(reference list* or bibliograph* or hand search* or manual search* or relevant journals).ab.
60.	(search strategy or search criteria or systematic search or study selection or data extraction).ab.
61.	(search* adj4 literature).ab.
62.	(medline or pubmed or 4ochrane or embase or psychlit or psyclit or psychinfo or psycinfo or CINAHL or science citation index or bids or cancerlit).ab.
63.	4ochrane.ab.
64.	((multiple treatment* or indirect or mixed) adj2 comparison*).ti,ab.
65.	or/55-64
66.	54 or 65
67.	31 and 45 and 66
68.	limit 67 to "all adult (19 plus years)"

2.2 ; Embase search strategy

1.	*low back pain/
2.	*sciatica/
3.	*radiculopathy/
4.	*radicular pain/
5.	*radiculitis/
6.	exp *"nerve root injury"/
7.	(backache* or lumbago or sciatica).ti,ab.
8.	((lumbar or lumbosacral or lumbo-sacral or back) adj5 (pain* or ache* or aching)).ti,ab.
9.	(radiculopathy or radiculitis or radicular pain*).ti,ab.
10.	(nerve root* adj5 (pain* or avulsion or compress* or disorder* or pinch* or inflam* or imping* or irritat* or entrap* or trap*)).ti,ab.
11.	or/9-10
12.	(back* or lumbosacral or lumbo-sacral or lumbar).ti,ab.
13.	11 and 12
14.	or/1-8,13
15.	letter.pt. or letter/
16.	note.pt.
17.	editorial.pt.
18.	case report/ or case study/
19.	(letter or comment*).ti.
20.	or/15-19
21.	randomized controlled trial/ or random*.ti,ab.
22.	20 not 21
23.	animal/ not human/
24.	nonhuman/
25.	exp animal experiment/
26.	exp experimental animal/
27.	animal model/
28.	exp rodent/
29.	(rat or rats or mouse or mice).ti.
30.	or/22-29
31.	14 not 30
32.	limit 31 to english language
33.	exp anticonvulsants/ or exp anticonvulsant agent/
34.	exp gabapentin/ or (gabapentin or neurontin).mp.
35.	exp pregabalin/ or pregabalin.mp. or lyrica.mp.
36.	Antidepressive Agents, Tricyclic/
37.	exp antidepressive agents/
38.	(antidepress* or anti-depress*).mp.
39.	serotonin norepinephrine reuptake inhibitor*.mp.
40.	snri.mp.

41.	exp amitriptyline/ or (amitriptyline or elavil or vanatrip).mp. or exp nortriptyline/ or nortriptyline.mp. or exp desipramine/ or desipramine.mp.
42.	Duloxetine.mp. or exp Duloxetine Hydrochloride/ or exp venlafaxine/ or venlafaxine.mp.
43.	or/33-42
44.	random*.ti,ab.
45.	factorial*.ti,ab.
46.	(crossover* or cross over*).ti,ab.
47.	((doubl* or singl*) adj blind*).ti,ab.
48.	(assign* or allocat* or volunteer* or placebo*).ti,ab.
49.	crossover procedure/
50.	double blind procedure/
51.	single blind procedure/
52.	randomized controlled trial/
53.	or/44-52
54.	systematic review/
55.	meta-analysis/
56.	(meta analy* or metanaly* or metaanaly*).ti,ab.
57.	((systematic or evidence) adj3 (review* or overview*)).ti,ab.
58.	(reference list* or bibliograph* or hand search* or manual search* or relevant journals).ab.
59.	(search strategy or search criteria or systematic search or study selection or data extraction).ab.
60.	(search* adj4 literature).ab.
61.	(medline or pubmed or cochrane or embase or psychlit or psyclit or psychinfo or psycinfo or CINAHL or science citation index or bids or cancerlit).ab.
62.	((pool* or combined) adj2 (data or trials or studies or results)).ab.
63.	cochrane.ab.
64.	or/54-63
65.	53 or 64
66.	32 and 43 and 65
67.	limit 66 to adult <18 to 64 years>
68.	limit 66 to aged <65+ years>
69.	67 or 68

2.3 ; APA psyc info search strategy

S9 AND S10	**Expanders** - Apply equivalent subjects<br>**Search modes** - Boolean/Phrase
S10	(tricyclic antidepressant/ or amitriptyline/ or nortriptyline/ or desipramine/ (amitriptyline or nortriptyline or despramiine or elavil).ti,ab. or snri/ or duloxetine/ or duloxetine.ti,ab. or venlafaxine/ or venlafaxine.ti,ab. or gabapentoid/ or gabapentin/ or (gabapentin or neurontin).ti,ab. or pregabalin/ or (pregabalin or lyrica).ti,ab.)
S9	S1 OR S2 OR S3 OR S8
S8	S6 AND S7
S7	back* or lumbosacral or lumbo-sacral or lumbar.ti,ab.
S6	S4 OR S5
S5	nerve root* NEAR/5 pain* or avulsion or compress* or disorder* or pinch* or inflam* or imping* or irritat* or entrap* or trap*.ti,ab.
S4	radiculopathy or radiculitis or radicular pain*.ti,ab.
S3	backache* or lumbago or sciatica.ti,ab.
S2	lumbar or lumbosacral or lumbo-sacral or back near/5 pain* or ache* or aching.ti,ab.
S1	back pain/

2.4 ; CINAHL (EBSCO) search strategy

S14 AND S19	**Limiters** - Peer Reviewed; Age Groups: All Adult<br>**Search modes** - Boolean/Phrase
S20	S14 AND S19
S19	S15 OR S16 OR S17 OR S18
S18	pregabalin or lyrica
S17	gabapentoid or gabapentin or neurontin
S16	snri or duloxetine or venlafaxine
S15	amitriptyline or nortriptyline or desipramine or elavil
S14	S11 not S12 (limited to English)

S13	S11 not S12
S12	PT anecdote or PT audiovisual or PT bibliography or PT biography or PT book or PT book review or PT brief item or PT cartoon or PT commentary or PT computer program or PT editorial or PT games or PT glossary or PT historical material or PT interview or PT letter or PT listservs or PT masters thesis or PT obituary or PT pamphlet or PT pamphlet chapter or PT pictorial or PT poetry or PT proceedings or PT "questions and answers" or PT response or PT software or PT teaching materials or PT website
S11	S1 or S2 or S3 or S4 or S5 or S10
S10	S8 and S9
S9	back* or lumbosacral or lumbo-sacral or lumbar
S8	S6 or S7
S7	nerve root* n5 (pain* or avulsion or compress* or disorder* or pinch* or inflam* or imping* or irritat* or entrap* or trap*)
S6	radiculopathy or radiculitis or radicular pain*
S5	backache* or lumbago or sciatica
S4	(lumbar or lumbosacral or lumbo-sacral or back) N5 (pain* or ache* or aching)
S3	(MH "radiculopathy")
S2	(MH "sciatica")
S1	(MH "low back pain")

2.5 ; Cochrane search strategy

#1	[mh "low back pain"]
#2	[mh sciatica]
#3	[mh radiculopathy]
#4	((lumbar or lumbosacral or lumbo-sacral or back) near/5 (pain* or ache* or aching)):ti,ab,kw
#5	(backache* or lumbago or sciatica):ti,ab,kw
#6	(radiculopathy or radiculitis or radicular pain*):ti,ab,kw
#7	(nerve root* near/5 (pain* or avulsion or compress* or disorder* or pinch* or inflam* or imping* or irritat* or entrap* or trap*)):ti,ab,kw
#8	{or #6-#7}
#9	(back* or lumbosacral or lumbo-sacral or lumbar):ti,ab,kw
#10	#8 and #9
#11	{or #1-#5, #10}
#12	[mh "antidepressive agents"]
#13	(antidepress* or anti-depress*):ti,ab,kw
#14	serotonin norepinephrine reuptake inhibitor*:ti,ab,kw
#15	(snri):ti,ab,kw
#16	[mh "tricyclic antidepressive agents"]
#17	(tricyclic antidepress* or anti-depress*):ti,ab,kw
#18	(amitriptyline or elavil or vanatrip or nortriptyline or desipramine or duloxetine or Duloxetine Hydrochloride or venlafaxine):ti,ab,kw
#19	[mh anticonvulsants]
#20	(gabapentin or neurontin or pregabalin or lyrica):ti,ab,kw
#21	{or #12-#20}
#22	#11 and #21

2.6 ; Clinical trials.gov and iCTRP search strategy

Low back pain AND (antidepressants or tricyclic or amitriptyline or elavil or nortriptyline or despiramine or duloxetine or venlafaxine or serotonin norepinephrine reuptake inhibitor or gabapentin or neurotin or lyrica or pregabalin)

Sciatica AND (antidepressants or tricyclic or amitriptyline or elavil or nortriptyline or despiramine or duloxetine or venlafaxine or serotonin norepinephrine reuptake inhibitor or gabapentin or neurotin or lyrica or pregabalin)

Online resource 3: Exclusion Criteria

Comparative effectiveness studies

1. Ward NG. Tricyclic antidepressants for chronic low-back pain. Mechanisms of action and predictors of response. Spine. 1986;11(7):661-5.
2. Sumracki NM, Hutchinson MR, Gentgall M, Briggs N, Williams DB, Rolan P. The effects of pregabalin and the glial attenuator minocycline on the response to intradermal capsaicin in patients with unilateral sciatica. PLoS ONE. 2012;7(6):e38525. doi: https://dx.doi.org/10.1371/journal.pone.0038525.
3. Stein D, Peri T, Edelstein E, Elizur A, Floman Y. The efficacy of amitriptyline and acetaminophen in the management of acute low back pain. Psychosomatics. 1996;37(1):63-70.
4. Singh NK, Jain H, Upadhyay M, Singh A. "To evaluate Efficacy and Safety for Management of Neuropathic Pain with Gabapentin, Pregabalin and Amitriptyline". NeuroQuantology. 2022;20(9):2891-6. doi: https://dx.doi.org/10.14704/nq.2022.20.9.NQ44336.
5. Singh A, Aggarwal S, Behal T. To study the comparison of functional outcomes between pregabalin versus gabapentin in Cases of Low Back Ache with radiculopathy. Journal of cardiovascular disease research. 2021;12(6):613-6. doi: 10.31838/jcdr.2021.12.06.82.
6. Schreiber S, Vinokur S, Shavelzon V, Pick CG, Zahavi E, Shir Y. A randomized trial of fluoxetine versus amitriptyline in musculo-skeletal pain. Isr J Psychiatry Relat Sci. 2001;38(2):88-94.
7. Sakai Y, Ito K, Hida T, Ito S, Harada A. Pharmacological management of chronic low back pain in older patients: a randomized controlled trial of the effect of pregabalin and opioid administration. Eur Spine J. 2015;24(6):1309-17. doi: https://dx.doi.org/10.1007/s00586-015-3812-6.
8. Romano CL, Romano D, Bonora C, Mineo G. Pregabalin, celecoxib, and their combination for treatment of chronic low-back pain. J. 2009;10(4):185-91. doi: https://dx.doi.org/10.1007/s10195-009-0077-z.
9. Robertson KL. Effect of gabapentin vs pregabalin on pain intensity in adults with chronic sciatica: a randomized clinical trial (JAMA Neurology (2018) DOI: 10.1001/jamaneurol.2018.3077). JAMA Neurol. 2019;76(1):117.
10. Park JH, Yeom JS, Park SM, Ryu MW, Kim HJ. Comparative study on the efficacy of pregabalin versus limaprost in patients with lumbar spinal stenosis: a prospective, randomized controlled trial. World Neurosurg. 2024. doi: 10.1016/j.wneu.2024.04.033.
11. Park JH, Yeom JS, Park SM, Ryu MW, Kim HJ. World Neurosurgery. 2024.
12. Otto JC, Forstenpointner J, Sachau J, Hullemann P, Hukauf M, Keller T, et al. A Novel Algorithm to Identify Predictors of Treatment Response: Tapentadol Monotherapy or Tapentadol/Pregabalin Combination Therapy in Chronic Low Back Pain? Frontiers in Neurology. 2019;10 (no pagination). doi: http://dx.doi.org/10.3389/fneur.2019.00979.
13. NCT03364075. Genetic Variants Associated With Low Back Pain and Their Response to Treatment With Duloxetine or Propranolol. 2016.
14. Mazza M, Mazza O, Pazzaglia C, Padua L, Mazza S. Escitalopram 20 mg versus duloxetine 60 mg for the treatment of chronic low back pain. Expert Opin Pharmacother. 2010;11(7):1049-52. doi: https://dx.doi.org/10.1517/14656561003730413.
15. Ko S. The effectiveness of oral corticosteroids for management of lumbar radiating pain: randomized, controlled trial study. Cios clinics in orthopedic surgery 8 (3) (pp 262-267), 2016 Date of publication: september 2016. 2016. doi: 10.4055/cios.2016.8.3.262.
16. Kct. Clinical study of a deeply-inserted acupotomy applied to Hyeopcheok points compared to a usual western-medicine care for lumbosacral radiculopathy : effectiveness, safety, cost-effectiveness(A three-arm randomized controlled, parallel study, pilot study, assessor-blind). https://trialsearchwhoint/Trial2aspx?TrialID=KCT0008945. 2023.

17. Kantito S, Tantisiriwat N, Piravej K. Comparison of the effectiveness between generic and original form of gabapentin for pain relief in suspected neuropathic component of low back pain. J Med Assoc Thai. 2014;97(7):767-75.
18. Kalita J, Kohat AK, Misra UK, Bhoi SK. An open labeled randomized controlled trial of pregabalin versus amitriptyline in chronic low backache. J Neurol Sci. 2014;342(1-2):127-32. doi: https://dx.doi.org/10.1016/j.jns.2014.05.002.
19. jRCT J. Randomised controlled study of the efficacy of duloxetine in patients with chronic low back pain suspected of central sensitisation. https://trialsearchwhoint/Trial2aspx?TrialID=JPRN-jRCT1041210147. 2022.
20. JPRN-jRCT1041210147. Randomised controlled study of the efficacy of duloxetine in patients with chronic low back pain suspected of central sensitisation. 2022.
21. Hwang CJ, Lee JH, Kim JH, Min SH, Park KW, Seo HY, et al. Gabapentin versus Transdermal Fentanyl Matrix for the Alleviation of Chronic Neuropathic Pain of Radicular Origin: A Randomized Blind Multicentered Parallel-Group Noninferiority Trial. Pain Res Manag. 2019;2019:4905013. doi: https://dx.doi.org/10.1155/2019/4905013.
22. Huzaif S, Hussain SM, Moosa SM, Khan IN, Kausar A, Zubair Q. Comparative Study of Efficacy and Safety of Gabapentin and Amitriptyline in Treatment of Neuropathic Pain Associated with Chronic Lumbar Radiculopathy. An Open Label, Prospective Randomized Clinical Study. European Journal of Molecular and Clinical Medicine. 2022;9(3):5024-31.
23. Gammoh O, Al-Smadi A, Shawagfeh MQ, Abadi T, Kasawneh J, Malkawi S, et al. The Clinical Difference between Gabapentin and Pregabalin: data from a Pilot Comparative Trial. Reviews on recent clinical trials. 2021;16(3):279-87. doi: 10.2174/1574887116666210201110507.
24. Farajirad S, Behdani F, Hebrani P, Farajirad M. Comparison between the effects of amitriptyline and bupropione on the quality of life and the reduction in the severity of pain in patients with chronic low-back pain. Neurosurgery Quarterly. 2013;23(4):227-9. doi: http://dx.doi.org/10.1097/WNQ.0b013e3182817d55.
25. Euctr ES. Phase 2 trial, conducted in more than one site, in which no one knows the treatment assigned to the patient to evaluate the effect against pain and the safety of the study drug, DFL24412, respect a control drug (Ketoprofen Lysine Salt), in patient with chronic low back pain. https://trialsearchwhoint/Trial2aspx?TrialID=EUCTR2021-001629-38-ES. 2021.
26. Ebadi SS, Mirbolook A, Kazemian G, Manafi-Rasi A, Mousavi M, Ettehad H. Conservative treatment of low back pain in lumbar disc herniation: Comparison of three therapeutic regimens. Eur Spine J. 2020;29:2885. doi: https://dx.doi.org/10.1007/s00586-020-06630-1.
27. CTRI/2022/05/042387. Comparing effect of two medications pregabalin and duloxetine for tingling and numbness in patients with long lasting low back pain. 2022.
28. Cohen SP, Hanling S, Bicket MC, White RL, Veizi E, Kurihara C, et al. Epidural steroid injections compared with gabapentin for lumbosacral radicular pain: multicenter randomized double blind comparative efficacy study. Bmj. 2015;350:h1748. doi: https://dx.doi.org/10.1136/bmj.h1748.
29. Baron R, Martin-Mola E, Muller M, Dubois C, Falke D, Steigerwald I. Effectiveness and Safety of Tapentadol Prolonged Release (PR) Versus a Combination of Tapentadol PR and Pregabalin for the Management of Severe, Chronic Low Back Pain With a Neuropathic Component: A Randomized, Double-blind, Phase 3b Study. Pain pract. 2015;15(5):455-70. doi: https://dx.doi.org/10.1111/papr.12200.
30. Alharbi A. Comparative study to evaluate efficacy and safety for management of neuropathic pain with gabapentin, pregabalin, and amitriptyline. Pakistan Journal of Medical and Health Sciences. 2021;15(9):2995-8. doi: http://dx.doi.org/10.53350/pjmhs211592995.

Clinical trials

1. Ware M, Centre MUHCRIotMUH. Genetic Variants Associated With Low Back Pain and Their Response to Treatment With Duloxetine or Propranolol. https://ClinicalTrials.gov/show/NCT03364075; 2017.
2. Vienna MUo. Comparison of the Efficacy of Duloxetine With Placebo in Patients With Chronic Low Back Pain With a Radicular Component. https://ClinicalTrials.gov/show/NCT01166048; 2010.
3. Umin. The efficacy of duloxetine for neuropathic pain a comparison of pregabalin. https://trialsearchwhoint/Trial2aspx?TrialID=JPRN-UMIN000028475. 2017.
4. TCTR20190303001. A comparison of analgesic efficacy between Amitriptyline and Mianserin in chronic low back pain patients: A randomized double-blind controlled trial (comparative effectiveness). 2019.
5. Tctr Pnyr. Efficacy and safety of warm acupuncture compared to gabapentin in the management of pain in patients with sciatica in Bhutan: a randomized non-inferiority trial. https://trialsearchwhoint/Trial2aspx?TrialID=TCTR20220211005. 2022.
6. Tctr Pnyr. Effectiveness of pregabalin for lumbar stenosis: a randomized, controlled trial. Pending not yet recruiting 27/04/23. https://trialsearchwhoint/Trial2aspx?TrialID=TCTR20190314001. 2019.
7. Tctr. A comparison of analgesic efficacy between Amitriptyline and Mianserin in chronic low back pain patients: a randomized double-blind controlled trial. https://trialsearchwhoint/Trial2aspx?TrialID=TCTR20190303001. 2018.
8. Snapinn SM. Evaluating the efficacy of a combination therapy. Statistics in medicine. 1987;6(6):657-65. doi: 10.1002/sim.4780060603.
9. Shaughnessy AF. Gabapentin equals epidural steroid for radicular pain. Am Fam Physician. 2015;92(9):814-20.
10. O'Connor AB. Study finds that the combination gabapentin plus nortriptyline reduces neuropathic pain more than either drug alone. Evidence-based medicine. 2010;15(2):45-6. doi: 10.1136/ebm1038.
11. NCT05851976. Duloxetine for LBP. 2023.
12. NCT04870957. The Back Pain Consortium Research Program Study (BAPAC emailed for data 27/04/23). 2021.
13. NCT01914666. An Open Label Extension Study of Duloxetine (LY248686) in Participants With Chronic Low Back Pain. 2013.
14. NCT01855919. A Study of Duloxetine (LY248686) in Participants With Chronic Low Back Pain. 2013.
15. NCT01166048. Comparison of the Efficacy of Duloxetine With Placebo in Patients With Chronic Low Back Pain With a Radicular Component. 2010.
16. NCT00767806. A Study for Patient With Chronic Low Back Pain. 2008.
17. NCT00424593. Duloxetine Versus Placebo in Chronic Low Back Pain. 2007.
18. NCT00408876. Duloxetine Versus Placebo in Chronic Low Back Pain. 2006.
19. NCT00388414. Imaging Study of Chronic Low Back Pain in Patients Taking Pain Medication. 2006.
20. NCT00108550. Chronic Low Back Pain Research Project. 2005.
21. NCT00018200. Effect of Antidepressants on Back Pain. 2001.
22. Nct ct. Management of Pain Associated With Failed Back Surgery Syndrome. https://clinicaltrialsgov/show/NCT05324761. 2022.
23. Nct. Tizanidine vs. Zolpidem in Primary Insomnia: a Randomized Trial. https://clinicaltrialsgov/ct2/show/NCT06303076. 2024.
24. Nct. Comparison of Unilateral and Bilateral Transforaminal Epidural Steroid Injection. https://clinicaltrialsgov/ct2/show/NCT06240793. 2024.
25. Nct. Focal Microvibration and Chronic Lumbosacral Radicular Pain. https://clinicaltrialsgov/ct2/show/NCT06301061. 2024.
26. Nct. Radiofrequency Ablation for the Treatment of Post-knee Arthroplasty Chronic Pain. https://clinicaltrialsgov/ct2/show/NCT05920382. 2023.
27. Nct. The Lumbar Interbody Fusion vs. Multidisciplinary Rehabilitation (LIFEHAB) Trial. https://clinicaltrialsgov/ct2/show/NCT06169488. 2023.

28. Nct. Effect of Catheter Type on Efficacy of Percutaneous Caudal Adhesolysis. https://clinicaltrialsgov/ct2/show/NCT06051149. 2023.
29. Nct. Short-term Effectiveness of Gabapentin Versus Placebo in Acute Lumbosacral Radiculalgia by Herniation Disc (GRADE) (emailed 07/10/22). https://clinicaltrialsgov/show/NCT04865042. 2021.
30. Nct. Clinical Trial of Pregabalin and COX2 in Spinal Stenosis. https://clinicaltrialsgov/show/NCT03584074. 2018.
31. Nct. Effect of Combined Morphine and Duloxetine on Chronic Pain (No results data avaiable, put into no outcome data group). https://clinicaltrialsgov/show/NCT03249558. 2017.
32. Nct. Genetic Variants Associated With Low Back Pain and Their Response to Treatment With Duloxetine or Propranolol. https://clinicaltrialsgov/show/NCT03364075. 2017.
33. Nct. Efficacy and Safety Study of Celecoxib and Pregabalin Compared With Celecoxib Monotherapy, in Patients With Chronic Low Back Pain Having a Neuropathic Component. https://clinicaltrialsgov/show/NCT01838044. 2013.
34. Nct. A Study of Duloxetine (LY248686) in Participants With Chronic Low Back Pain. https://clinicaltrialsgov/show/NCT01855919. 2013.
35. Nct. Duloxetine in Osteoarthritis (OA) Pain. https://clinicaltrialsgov/show/NCT01558700. 2012.
36. Nct. Comparison of Increasing Doses of Tapentadol Versus a Combination of Tapentadol and Pregabalin. https://clinicaltrialsgov/show/NCT01352741. 2011.
37. Nct. Steroids Versus Gabapentin. https://clinicaltrialsgov/show/NCT01495923. 2011.
38. Nct. Linking Altered Central Pain Processing and Genetic Polymorphism to Drug Efficacy in Chronic Low Back Pain (Predictio). https://clinicaltrialsgov/show/NCT01179828. 2010.
39. Nct. Comparison of the Efficacy of Duloxetine With Placebo in Patients With Chronic Low Back Pain With a Radicular Component. https://clinicaltrialsgov/show/NCT01166048. 2010.
40. Nct. Efficacy of Pregabalin in Patients With Radicular Pain. https://clinicaltrialsgov/show/NCT00908375. 2009.
41. Nct. Efficacy of Antidepressants in Chronic Back Pain. https://clinicaltrialsgov/show/NCT00964886. 2009.
42. Nct. A Study for Patient With Chronic Low Back Pain. https://clinicaltrialsgov/show/NCT00767806. 2008.
43. Nct. Duloxetine Versus Placebo in Chronic Low Back Pain. https://clinicaltrialsgov/show/NCT00424593. 2007.
44. Nct. Imaging Study of Chronic Low Back Pain in Patients Taking Pain Medication. https://clinicaltrialsgov/show/NCT00388414. 2006.
45. Nct. Duloxetine Versus Placebo in Chronic Low Back Pain. https://clinicaltrialsgov/show/NCT00408876. 2006.
46. Nct. Chronic Low Back Pain Research Project. https://clinicaltrialsgov/show/NCT00108550. 2005.
47. Nct. Trial of Efficacy and Safety of Pregabalin in Subjects With Neuropathic Pain Associated With Lumbo-Sacral Radiculopathy. https://clinicaltrialsgov/show/NCT00159705. 2005.
48. Nct. Effect of Antidepressants on Back Pain. https://clinicaltrialsgov/show/NCT00018200. 2001.
49. Nct. Pain Treatment for Sciatica. https://clinicaltrialsgov/show/NCT00009672. 2001.
50. Lilly E, Company, Shionogi. An Open Label Extension Study of Duloxetine (LY248686) in Participants With Chronic Low Back Pain. https://ClinicalTrials.gov/show/NCT01914666; 2013.
51. Lilly E, Company, Shionogi. A Study of Duloxetine (LY248686) in Participants With Chronic Low Back Pain. https://ClinicalTrials.gov/show/NCT01855919; 2013.
52. Lilly E, Company. A Study for Patient With Chronic Low Back Pain. https://ClinicalTrials.gov/show/NCT00767806; 2008.
53. Lilly E, Company. Duloxetine Versus Placebo in Chronic Low Back Pain. https://ClinicalTrials.gov/show/NCT00424593; 2007.
54. Krebs EE, Jensen AC, Nugent S, DeRonne B, Rutks I, Leverty D, et al. Design, recruitment outcomes, and sample characteristics of the Strategies for Prescribing Analgesics Comparative

Effectiveness (SPACE) trial. Contemporary Clinical Trials. 2017;62:130-9. doi: http://dx.doi.org/10.1016/j.cct.2017.09.003.
55. jRCTs J. Studies on pain control in musculoskeletal chronic pain diseases (comparative effectiveness). https://trialsearchwhoint/Trial2aspx?TrialID=JPRN-jRCTs031190172. 2020.
56. JPRN-UMIN000039713. The efficacy of a combination of exercise and duloxetine on physical disability in patients with non-specific chronic low back pain (single arm non randmoised). 2020.
57. JPRN-JapicCTI-132312. An open label extension study of phase 3 clinical trial of duloxetine in patients with chronic low back pain. 2013.
58. JPRN-JapicCTI-132129. A phase 3 clinical trial of duloxetine in patients with chronic low back pain. 2013.
59. Jprn U. The efficacy of duloxetine for neuropathic pain a comparison of pregabalin. https://trialsearchwhoint/Trial2aspx?TrialID=JPRN-UMIN000028475. 2017.
60. Irct20200620047852N. "Agomelatin in chronic low back pain" (both groups recieve PG therefore not appropriate intervention. https://trialsearchwhoint/Trial2aspx?TrialID=IRCT20200620047852N1. 2020.
61. Irct20191210045685N po. Effectiveness of duloxetine in chronic low back pain (post op so not appropriate). https://trialsearchwhoint/Trial2aspx?TrialID=IRCT20191210045685N1. 2020.
62. IRCT201303096480N4. Efficacy of oral gabapentin on pain intensity in patients with acute low back pain patients. 2013.
63. Inc. PsUhmwMtfV, Pfizer. PROs in Chronic Low Back Pain Patients With Accompanying Lower Limb Pain (Neuropathic Component) Treated With Pregabalin. https://ClinicalTrials.gov/show/NCT02273908; 2014.
64. Inc. PsUhmwMtfV, Pfizer. Efficacy and Safety Study of Celecoxib and Pregabalin Compared With Celecoxib Monotherapy, in Patients With Chronic Low Back Pain Having a Neuropathic Component. https://ClinicalTrials.gov/show/NCT01838044; 2013.
65. Hospital MG. Effect of Combined Morphine and Duloxetine on Chronic Pain. https://ClinicalTrials.gov/show/NCT03249558; 2018.
66. Hassett AL, Williams DA, Harris RE, Harte SE, Kaplan CM, Schrepf A, et al. An Interventional Response Phenotyping Study in Chronic Low Back Pain: Protocol for a Mechanistic Randomized Controlled Trial. The Back Pain Consortium Research Program Study (BAPAC emailed for data 27/04/23). Pain Med. 2023;27. doi: https://dx.doi.org/10.1093/pm/pnad005.
67. Hassett AL, Williams DA, Harris RE, Harte SE, Kaplan CM, Schrepf A, et al. Pain Medicine (United States). 2023;24(1 S):S126-S38.
68. Hassett AL, Williams DA, Harris RE, Harte SE, Kaplan CM, Schrepf A, et al. An Interventional Response Phenotyping Study in Chronic Low Back Pain: protocol for a Mechanistic Randomized Controlled Trial. The Back Pain Consortium Research Program Study (BAPAC emailed for data 27/04/23). Pain medicine (Malden, Mass). 2023. doi: 10.1093/pm/pnad005.
69. EUCTR2010-022955-43-FI. OPEN LABEL EUROPEAN STUDY TO SUPPORT THE EARLY IDENTIFICATION OF PATIENTS WITH CHRONIC NEUROPATHIC LOW BACK PAIN IN PRIMARY CARE AND TO ASSESS THE EFFECTIVENESS AND TOLERABILITY OF PREGABALIN IN THIS POPULATION
. 2011.
70. EUCTR2008-002248-40-NL. Effect of Duloxetine 60 mg Once Daily versus Placebo in Patients with Chronic Low Back Pain - HMGC. 2008.
71. Euctr NL. Effect of Duloxetine 60 mg to 120 mg Once Daily in Patients with Chronic Low Back Pain - HMEN. https://trialsearchwhoint/Trial2aspx?TrialID=EUCTR2006-003484-31-NL. 2006.
72. Euctr ES. Efecto de Duloxetina 60 mg, Administrado una Vez al Día, Frente a Placebo, en Pacientes con Lumbalgia Crónica Effect of Duloxetine 60 mg Once Daily versus Placebo in Patients with Chronic Low Back Pain - HMGC. https://trialsearchwhoint/Trial2aspx?TrialID=EUCTR2008-002248-40-ES. 2008.
73. Euctr DE. Evaluation of the effectiveness, safety, and tolerability of tapentadol PR versus a combination of tapentadol PR and pregabalin in subjects with severe chronic low back pain with a

neuropathic pain component. https://trialsearchwhoint/Trial2aspx?TrialID=EUCTR2010-019998-14-DE. 2010.
74. Euctr DE. A Randomised Placebo-Controlled Trial of the Efficacy and Safety of Pregabalin in the Treatment of Subjects with Neuropathic Pain Associated with Lumbo-sacral Radiculopathy. https://trialsearchwhoint/Trial2aspx?TrialID=EUCTR2004-002948-10-DE. 2005.
75. Euctr AT. Randomized Double-blind Study Comparing the Efficacy of Duloxetine with Placebo in Patients with Chronic Low Back Pain. https://trialsearchwhoint/Trial2aspx?TrialID=EUCTR2009-012713-22-AT. 2010.
76. Eli L, Company. Protocol F1J-MC-HMEN: effect of Duloxetine 60 mg to 120 mg Once Daily in Patients With Chronic Low Back Pain. Clinicaltrialsgov. 2007.
77. Eli L, Company. Protocol F1J-MC-HMEO: duloxetine Versus Placebo in the Treatment of Chronic Low Back Pain. Clinicaltrialsgov. 2006.
78. Deyo RA. Efficacy and safety of duloxetine in patients with chronic low back pain. Spine. 2010;35(13) :(pp E586), 2010. Date of Publication: 01 Jun 2010).
79. Dental NIo, Research C, Center NIoHC. Pain Treatment for Sciatica. https://ClinicalTrials.gov/show/NCT00009672; 2001.
80. CTRI/2012/07/002833. A clinical trial to study the effects of two drugs, amitriptyline and pregabalin in patients with chronic low back pain. 2012.
81. Ctri. Comparing effect of two medications pregabalin and duloxetine for tingling and numbness in patients with long lasting low back pain (comparative effectivness). https://trialsearchwhoint/Trial2aspx?TrialID=CTRI/2022/05/042387. 2022.
82. Ctri. A clinical trial to study the effects of two drugs, ibuprofen and pregabalin in patients with acute back pain with leg pain (comparative effectivness). https://trialsearchwhoint/Trial2aspx?TrialID=CTRI/2020/08/026968. 2020.
83. Ctri. A clinical trial to study the efficacy and safety of combination drugs of Pregabalin Prolonged Release and Etoricoxib in comparison to single therapy of Etoricoxib in patients having chronic low back pain in India. https://trialsearchwhoint/Trial2aspx?TrialID=CTRI/2018/10/015886. 2018.
84. Ctri. Comparison of 3 drugs in treating low back pain with lower limb pain. https://trialsearchwhoint/Trial2aspx?TrialID=CTRI/2017/06/008812. 2017.
85. Clark JD, Bair MJ, Belitskaya-Levy I, Fitzsimmons C, Zehm LM, Dougherty PE, et al. Sequential and comparative evaluation of pain treatment effectiveness response (SCEPTER), a pragmatic trial for conservative chronic low back pain treatment. Contemporary Clinical Trials. 2023;125(no pagination).
86. Affairs UDoV, Research VOo, Development. Chronic Low Back Pain Research Project. https://ClinicalTrials.gov/show/NCT00108550; 2004.
87. Affairs UDoV, Research VOo, Development. Effect of Antidepressants on Back Pain. https://ClinicalTrials.gov/show/NCT00018200; 1999.
88. ACTRN12613000559718. Pregabalin versus Gabapentin in the Treatment of Sciatica. 2013.
89. ACTRN12612000131853. Is amitriptyline effective in the management of chronic low back pain? 2012.

Conference abstracts

1. Urquhart DM, Wluka AE, Sim MR, van Tulder M, Forbes A, Gibson SJ, et al. Is low-dose amitriptyline effective in the management of chronic low back pain? Study protocol for a randomised controlled trial. Trials. 2016;17(1):514.
2. Urquhart D, Wluka A, Van Tulder M, Heritier S, Forbes A, Fong C, et al. Efficacy of low-dose amitriptyline for chronic low back pain: A double-blind, randomised controlled trial. Osteoarthritis and Cartilage. 2018;26(Supplement 1):S416.

3. Tolle TR, Baron R, Freynhagen R, Leon T, Murphy TK, Phillips TR. The efficacy and safety of pregabalin in the treatment of neuropathic pain associated with lumbo-sacral radiculopathy. European jounral of neurology. 2008;15(Suppl 3):171, Abstract no: P1540.
4. Steigerwald I, Kern KU, Buunen M, Baron R, Falke D. Effectiveness of tapentadol prolonged release (PR) versus a combination of tapentadol PR and pregabalin for managing severe, chronic low back pain with a neuropathic component. Regional anesthesia and pain medicine. 2013;38(1).
5. Skljarevski V, Zhang S, Desaiah D, Palacios S, Miazgowski T, Patrickm K. Effect of duloxetine 60 mg once daily versus placebo in patients with chronic low back pain: A 12-week, randomized, double-blind trial. Pain Med. 2010;11(2):322. doi: http://dx.doi.org/10.1111/j1526-4637.2009.00781.x.
6. Skljarevski V, Zhang S, Desaiah D, Palacios S, Miazgowski T, Patrick K. Efficacy and safety of duloxetine 60 mg once-daily in patients with chronic low back pain. J Pain. 2010;1):S38. doi: http://dx.doi.org/10.1016/j.jpain.2010.01.158.
7. Skljarevski V, Desaiah D, Liu-Seifert H, et al. Efficacy of duloxetine in chronic low back pain. Eur J Neurol. 2008;15(Suppl 3):320, Abstract no: P2447.
8. Skljarevski V, Bair MJ, Ossanna MJ, Frakes E, Zhang S, Alaka K. OMERACT responder analysis of patients treated with duloxetine for chronic low back pain. Arthritis and Rheumatism. 2010;10):175. doi: http://dx.doi.org/10.1002/art.27944.
9. Schukro RP, Oehmke M, Reitinger C, Geroldinger A, Heinze G, Pramhas S. Efficacy of duloxetine versus placebo in patients with chronic low back pain and a neuropathic component. European journal of anaesthesiology. 2014;31(var.pagings):215.
10. Robertson K, Marshman LAG, Hennessy M, Harriss L, Plummer D. Pregabalin versus gabapentin in the treatment of sciatica: Study protocol for a randomised, double-blind, cross-over trial (PAGPROS). Trials. 2018;19(1) (no pagination). doi: http://dx.doi.org/10.1186/s13063-017-2400-y.
11. Mathieson S, Maher CG, McLachlan AJ, Latimer J, Koes BW, Hancock MJ, et al. PRECISE - pregabalin in addition to usual care for sciatica: study protocol for a randomised controlled trial. Trials. 2013;14:213. doi: 10.1186/1745-6215-14-213.
12. Mathieson S, Billot L, Maher CG, McLachlan AJ, Latimer J, Koes BW, et al. PRECISE - pregabalin in addition to usual care: Statistical analysis plan. Trials. 2016;17(1) (no pagination). doi: http://dx.doi.org/10.1186/s13063-016-1174-y.
13. Johnson K, Chatterjee N, Noor N, Crowell A, McCue R, Mackey S. Effects of duloxetine and placebo in patients with chronic low back pain. No results data available- put into no outcome data group. J Pain. 2011;Conference: 30th Annual Scientific Meeting of the American Pain Society Austin, TX United States. Conference Start: 20110519 Conference End: 20110521. Conference Publication:(var.pagings). 12 :(4 SUPPL. 1) (pp P49), 2011. Date of Publication: April 2011).
14. GmbH G. Comparison of Increasing Doses of Tapentadol Versus a Combination of Tapentadol and Pregabalin. https://ClinicalTrials.gov/show/NCT01352741; 2011.
15. Gelijkens V, Van Zundert J, De Vooght P, Vander Laenen M, Heylen R, Vanelderen P. The effectiveness of amitriptyline in the treatment of subacute lumbar radicular pain. European journal of anaesthesiology. 2014;31:232.
16. Baron R, Kern KU, Buunen M, Steigerwald I, Falke D. Impact of tapentadol prolonged release (PR) versus a combination of tapentadol PR and pregabalin on the neuropathic component of severe, chronic low back pain. Regional anesthesia and pain medicine. 2013;38(1).
17. Al-hihi E, Badgett RG. 2017 - PRECISE In moderate-to-severe sciatica, pregabalin did not reduce leg pain intensity or improve quality of life. ACP journal club. 2017;167(2):3-. doi: 10.7326/ACPJC-2017-167-2-004.

Pains not separated (i.e, neck and back pain reported together)

1. Malik KM, Mnelson A, Javram M, Lee Robak S, Tbenzon H. Efficacy of Pregabalin in the Treatment of Radicular Pain: results of a Controlled Trial. Anesthesiology & pain medicine. 2015;5(4):e28110.

2. Chen L, Deng H, Houle T, Zhang Y, Ahmed S, Zhang W, et al. Comparison between acupuncture therapy and gabapentin for chronic pain: a pilot study. Dont seperate back and neck pain patients. Acupunct Med. 2021;39(6):619-28. doi: https://dx.doi.org/10.1177/09645284211026683.
**Post hoc analysis not relevant to research questions**<br><br>1. Wililamson OD, Schroer M, Ruff DD, Ahl J, Margherita A, Sagman D, et al. Onset of Response with Duloxetine Treatment in Patients with Osteoarthritis Knee Pain and Chronic Low Back Pain: a Post Hoc Analysis of Placebo-Controlled Trials. Clin Ther. 2014;36(4):544-51. doi: 10.1016/j.clinthera.2014.02.009.<br>2. Tsuji T. Response to duloxetine in chronic low back pain: exploratory post hoc analysis of a Japanese Phase III randomized study. Journal of pain research. 2017;10:2157-68.<br>3. Moore RA, Cai N, Skljarevski V, Tölle TR. Duloxetine use in chronic painful conditions--individual patient data responder analysis. European journal of pain (London, England). 2014;18(1):67-75. doi: 10.1002/j.1532-2149.2013.00341.x.<br>4. Itoh N, Uchio Y, Tsuji T, Ishida M, Ochiai T, Konno S. Efficacy of duloxetine in patients with knee osteoarthritis or chronic low back pain with early pain reduction: An exploratory post-hoc analysis of Japanese phase 3, 1-year extension studies. J Orthop Sci. 2022;27(3):717-24. doi: https://dx.doi.org/10.1016/j.jos.2021.02.016.<br>5. Itoh N, Uchio Y, Tsuji T, Ishida M, Ochiai T, Konno S. Efficacy of duloxetine in patients with knee osteoarthritis or chronic low back pain with early pain reduction: An exploratory post-hoc analysis of Japanese phase 3, 1-year extension studies. Journal of Orthopaedic Science. 2021. doi: http://dx.doi.org/10.1016/j.jos.2021.02.016.<br>6. Enomoto H, Fujikoshi S, Funai J, Sasaki N, Ossipov MH, Tsuji T, et al. Assessment of direct analgesic effect of duloxetine for chronic low back pain: post hoc path analysis of double-blind, placebo-controlled studies. Journal of pain research. 2017;10(pp 1357-1368).<br>7. Alev L, Fujikoshi S, Yoshikawa A, Enomoto H, Ishida M, Tsuji T, et al. Duloxetine 60 mg for chronic low back pain: Post hoc responder analysis of double-blind, placebo-controlled trials. Journal of Pain Research. 2017;10:1723-31. doi: http://dx.doi.org/10.2147/JPR.S138297.

Studies excluded at meta-analysis stage	Reason
Kim HJ, Kim JH, Park YS, et al. Comparative study of the efficacy of limaprost and pregabalin as single agents and in combination for the treatment of lumbar spinal stenosis: a prospective, double-blind, randomized[60]	Did not report variance measures and raw data could not be obtained from authors
Pheasant H, Bursk A, Goldfarb J, et al. Amitriptyline and chronic low-back pain.<br>A randomized double-blind<br>crossover study. *Spine* 1983;8(5):552-7.[52]	Did not report baseline measures of outcomes
Baron R, Freynhagen R, Tölle TR, et al. The efficacy and safety of pregabalin in the treatment of neuropathic pain associated with chronic lumbosacral radiculopathy. *Pain* 2010;150(3):420-27. doi: 10.1016/j.pain.2010.04.013 [63]	Used a placebo controlled withdrawal design, where all participants completed an initial single blind phase of pregabalin for 28 days, before randomisation. Post randomisation, the placebo group also remained on pregabalin taper for 7 days, preventing comparisons.

Online resource 4; GRADE judgement criteria

The certainty of evidence was initially set to high and downgraded by one level for each of the following domains: limitation of study design, inconsistency of results, imprecision, and publication bias. We did not upgrade the certainty of evidence for any reason, such as large effect size.
The certainty of evidence was categorized as follows[1]
• **High:** Further research is very unlikely to change the confidence in the estimate of effect.
• **Moderate:** Further research is likely to have an important impact in the confidence in the estimate of effect.
• **Low:** Further research is very likely to have an important impact on our confidence in the estimate of effect and is likely to change the estimate.
• **Very Low:** Any estimate of effect is very uncertain

The evidence was graded on the domains in the following manner:

**Study design**	The certainty of the evidence was downgraded: by ONE level: if >25% of participants were from a crossover trial that did not report the first phase results separately to reduce potential carryover effect.
**Risk of bias**	The outcomes from the Cochrane risk of bias 2 tool, which includes judgements about bias related to; the randomisation process; deviations from intended interventions; missing outcome data; measurement of the outcome; selection of the reported results, were judiciously considered when deciding the overall certainty of evidence.[2]<br>The certainty of the evidence was downgraded:<br>• By ONE level, if >25% of participants were from studies with high risk of bias.<br>• By ONE level, if >50% of participants were from studies with some concerns for risk of bias<br>• By TWO levels, if >50% of participants were from studies with high risk of bias
**Inconsistency**	Inconsistency measures the magnitude of intervention effect differences across studies and is evaluated based on the similarity of point estimate across studies, the extent of the overall of confidence intervals and the subsequent statistical tests of heterogeneity (I^2).<br>The certainty of the evidence was downgraded:<br>• By ONE level: if the statistical heterogeneity was moderate e.g. I^2 =>50%<br>• By TWO levels: if the statistical heterogeneity was large e.g. I^2 =>75%
**Imprecision**	The updated guidelines[3] for rating imprecision of systematic reviews, suggests results are imprecise when studies have wide confidence intervals around the estimate of the effect, and when this confidence interval crosses either the minimally clinically important difference (MDIC) or the null hypothesis threshold. In these cases, we downgrade the evidence resulting in uncertainty of the results. The MCID for pain was considered 10 points on a 100-point scale. The MCID for disability was considered 19 points, which is midway between the MCID for the two commonly used disability scores (Roland Morris disability questionnaire MCID of 21 points on a 100 point scale and Oswestry Disability questionnaire MCID of 17 points of a 100 point scale)[4] Rating down for imprecision based on optimal information size was not required as no studies had a large effect.[3]<br>The certainty of the evidence was downgraded:<br>• by ONE level, if the confidence interval was larger than 5 points and crossed the null hypothesis line or the minimally clinical important difference of 10 points on a 100 point scale for pain[5] and 19/100 points for disability[4]<br>• by TWO levels if, the confidence interval was larger than 5 points and crossed both the MCID and the null hypothesis suggesting two difference inferences of effect
**Indirectness**	Indirection[6] refers to a discrepancy between the population, intervention, comparator, or outcome for the included studies. criteria.<br>The certainty of evidence was downgraded:<br>• by ONE level, if there was indirectness in only one area of population, intervention, comparator or outcomes.<br>• by TWO levels, if there was indirectness in two or more areas of population, intervention, comparator or outcomes.
**Publication bias**	Publication bias is a systematic underestimate or an overestimate of the underlying beneficial or harmful effect due to the selective publication of studies.[7] The certainty of evidence was

	downgraded by ONE level if 10 or more studies were available meaning a funnel plot could be constructed and the funnel plot suggested publication bias.<br><br>Final outcomes were scored as important where the overall mean difference was larger than the minimal important difference.

References

1. Guyatt GH, Oxman AD, Vist GE, et al. GRADE: an emerging consensus on rating quality of evidence and strength of recommendations. *Bmj* 2008;336(7650):924-26.
2. Guyatt GH, Oxman AD, Vist G, et al. GRADE guidelines: 4. Rating the quality of evidence—study limitations (risk of bias). *Journal of clinical epidemiology* 2011;64(4):407-15.
3. Zeng L, Brignardello-Petersen R, Hultcrantz M, et al. GRADE Guidance 34: update on rating imprecision using a minimally contextualized approach. *Journal of clinical epidemiology* 2022;150:216-24. doi: 10.1016/j.jclinepi.2022.07.014
4. Maughan EF, Lewis JS. Outcome measures in chronic low back pain. *Eur Spine J* 2010;19:1484-94.
5. Ferreira ML, Herbert RD, Ferreira PH, et al. The smallest worthwhile effect of nonsteroidal anti-inflammatory drugs and physiotherapy for chronic low back pain: a benefit–harm trade-off study. *Journal of clinical epidemiology* 2013;66(12):1397-404.
6. Guyatt GH, Oxman AD, Kunz R, et al. GRADE guidelines: 8. Rating the quality of evidence—indirectness. *Journal of clinical epidemiology* 2011;64(12):1303-10.
7. Guyatt GH, Oxman AD, Montori V, et al. GRADE guidelines: 5. Rating the quality of evidence—publication bias. *Journal of clinical epidemiology* 2011;64(12):1277-82.

Online resource 5: GRADE judgements for outcomes

5.1 GRADE outcomes for pain outcomes overall, and subgrouped by certainty of NP in participants

Certainty assessment							№ of patients		Effect		Certainty	Importance
№ of studies	Study design	Risk of bias	Inconsistency	Indirectness	Imprecision	Other considerations	Neuropathic pain medication	placebo or usual care	Relative (95% CI)	Absolute (95% CI)		
**Short term -all outcomes**												
16	randomised trials	very serious[a]	very serious[b]	serious[c]	serious[d]	none	764	765	-	**9.3 lower** (13.71 lower to 4.88 lower)	⊕◯◯◯ Very low	NOT IMPORTANT
**Short term - definite NP**												
2	randomised trials	very serious[a]	very serious[e]	serious[c]	very serious[f]	none	51	47	-	MD **16.65 lower** (35.95 lower to 2.65 higher)	⊕◯◯◯ Very low	IMPORTANT
**Short term -probable NP**												
3	randomised trials	very serious[a]	not serious	serious[c]	serious[d]	none	206	209	-	MD **10.45 lower** (14.79 lower to 6.12 lower)	⊕◯◯◯ Very low	IMPORTANT
**Short term - possible NP**												

Certainty assessment							№ of patients		Effect		Certainty	Importance
№ of studies	Study design	Risk of bias	Inconsistency	Indirectness	Imprecision	Other considerations	Neuropathic pain medication	placebo or usual care	Relative (95% CI)	Absolute (95% CI)		
3	randomised trials	serious[g]	very serious[e]	serious[c]	very serious[f]	none	153	146	-	MD **5.5 lower** (20.52 lower to 9.52 higher)	⊕◯◯◯ Very low	NOT IMPORTANT
**Short term - unlikely NP**												
4	randomised trials	serious[g]	not serious	serious[c]	serious[d]	none	202	214	-	MD **6.67 lower** (10.58 lower to 2.76 lower)	⊕◯◯◯ Very low	NOT IMPORTANT
**Short term - unclear NP**												
4	randomised trials	very serious[a]	very serious[e]	serious[c]	very serious[f]	none	152	149	-	MD **8.93 lower** (20.57 lower to 2.71 higher)	⊕◯◯◯ Very low	NOT IMPORTANT
**Medium term - all outcomes**												
10	randomised trials	very serious[a]	not serious	serious[c]	not serious	none	1070	883	-	**5.49 lower** (7.24 lower to 3.74 lower)	⊕◯◯◯ Very low	NOT IMPORTANT
**Medium term certainty grouped - - possible, unclear or unlikely NP**												

Certainty assessment							№ of patients		Effect		Certainty	Importance
№ of studies	Study design	Risk of bias	Inconsistency	Indirectness	Imprecision	Other considerations	Neuropathic pain medication	placebo or usual care	Relative (95% CI)	Absolute (95% CI)		
9	randomised trials	very serious[a]	not serious	serious[c]	not serious	none	1031	844	-	MD **5.26 lower** (7.03 lower to 3.49 lower)	⨁◯◯◯ Very low	NOT IMPORTANT

CI: confidence interval; **MD:** mean difference

Explanations
a. => 50% of participants come from trials with high risk of bias
b. Heterogeneity I^2=>50%
c. Similar age, sex, duration, severity across studies, however studies explored several different classes of medication including TCAs, Anticonvulsants and SNRI.
d. Confidence interval crosses the minimal clinically important difference threshold of 10 points on a 100 point scale
e. Heterogeneity: I^2=> 75%
f. Confidence interval crosses both the null and the minimal clinically important difference of 10 points
g. =>25% of participants came from studies with high risk of bias

5.2 GRADE outcomes for disability outcomes overall, and subgrouped by certainty of NP in participants

Certainty assessment							№ of patients		Effect		Certainty	Importance
№ of studies	Study design	Risk of bias	Inconsistency	Indirectness	Imprecision	Other considerations	Neuropathic pain medication	placebo or usual care	Relative (95% CI)	Absolute (95% CI)		
**Short term- Definite / Probable NP**												
2	randomised trials	very serious[a]	not serious[b]	serious[c]	not serious	none	187	189	-	MD **9.25 lower** (12.59 lower to 5.9 lower)	⊕◯◯◯ Very low	NOT IMPORTANT
**Short term- possible, unclear or unlikely NP**												
6	randomised trials	serious[d]	very serious[e]	serious[c]	serious[f]	none	277	275	-	MD **1.57 lower** (8.93 lower to 5.82 higher)	⊕◯◯◯ Very low	NOT IMPORTANT
**Short term disability - all outcomes**												
8	randomised trials	very serious[a]	very serious[b]	serious[c]	serious[f]	none	464	464	-	**3.35 lower** (9 lower to 2.29 higher)	⊕◯◯◯ Very low	NOT IMPORTANT
**Medium term disability - all outcomes**												
9	randomised trials	serious[g]	not serious	serious[c]	not serious	none	1029	852	-	**4.06 lower** (5.63 lower to 2.48 lower)	⊕⊕◯◯ Low	NOT IMPORTANT

Medium term disability - possible, unlikely or unclear neuropathic pain

Certainty assessment							№ of patients		Effect		Certainty	Importance
№ of studies	Study design	Risk of bias	Inconsistency	Indirectness	Imprecision	Other considerations	Neuropathic pain medication	placebo or usual care	Relative (95% CI)	Absolute (95% CI)		
8	randomised trials	serious[g]	not serious	serious[c]	not serious	none	991	810	-	**4.96 lower** (7.24 lower to 2.68 lower)	⊕⊕◯◯ Low	NOT IMPORTANT

CI: confidence interval; **MD:** mean difference

Explanations

a. => 50% of participants came from studies with high risk of bias
b. Heterogeneity I^2< 50%
c. Similar age, sex, duration, severity across studies, however studies explored several different classes of medication including TCAs, Anticonvulsants and SNRI.
d. =>25% of participants came from studies with high risk of bias
e. Heterogeneity I^2=> 75%
f. The 95% confidence interval crosses the null g. => 50% of participants came from studies with some concerns for risk of bias

5.3 GRADE outcomes for TCA outcomes at primary end point, subgrouped by certainty of NP in participants

Certainty assessment							№ of patients		Effect		Certainty	Importance
№ of studies	Study design	Risk of bias	Inconsistency	Indirectness	Imprecision	Other considerations	TCA	placebo or usual care	Relative (95% CI)	Absolute (95% CI)		
**TCA pain outcomes - possible/ unlikely or unclear neuropathic pain**												
6	randomised trials	very serious[a]	not serious	not serious	serious[b]	none	216	187	-	MD **5.32 lower** (10.55 lower to 0.09 lower)	⊕◯◯◯ Very low	NOT IMPORTANT
**TCA disability outcomes - possible/ unlikely or unclear Neuropathic pain**												
5	randomised trials	serious[c]	serious[d]	not serious	not serious	none	183	157	-	MD **5.55 lower** (10.39 lower to 0.71 lower)	⊕⊕◯◯ Low	NOT IMPORTANT

CI: confidence interval; **MD:** mean difference

Explanations

a. => 50% of participants from studies with high risk of bias
b. 95% confidence interval crosses the minimal clinically important difference of 10 points
c. => 25% of participants from studies with high risk of bias
d. Heterogeneity I^2=>50%

5.4 GRADE outcomes for anticonvulsant outcomes at primary end point, subgrouped by certainty of NP in participants

Certainty assessment							№ of patients		Effect			
**№ of studies**	**Study design**	**Risk of bias**	**Inconsistency**	**Indirectness**	**Imprecision**	**Other considerations**	**Anticonvulsants**	**placebo or usual care**	**Relative (95% CI)**	**Absolute (95% CI)**	**Certainty**	**Importance**
**Anticonvulsant pain outcomes - possible/ unlikely or unclear neuropathic pain**												
7	randomised trials	very serious[a]	very serious[b]	not serious	very serious[c]	none	310	306	-	MD **7.63 lower** (16.17 lower to 0.91 lower)	⨁◯◯◯ Very low	IMPORTANT
**Anticonvulsant pain outcomes - probable / definite pain**												
4	randomised trials	very serious[a]	serious[d]	not serious	serious[e]	none	237	236	-	MD **11.81 lower** (118.32 lower to 5.31 lower)	⨁◯◯◯ Very low	NOT IMPORTANT
**Anticonvulsant disability outcomes - possible/ unlikely or unclear neuropathic pain**												
3	randomised trials	serious[f]	very serious[b]	not serious	serious[g]	none	190	186	-	**2.6 lower** (17.78 lower to 12.57 higher)	⨁◯◯◯ Very low	NOT IMPORTANT
**Anticonvulsant disability outcomes - probable/definite neuropathic pain**												
2	randomised trials	very serious[a]	not serious	not serious	not serious	none	187	189	-	MD **9.25 lower** (12.59 lower to 5.9 lower)	⨁⨁◯◯ Low	NOT IMPORTANT

CI: confidence interval; **MD:** mean difference

Explanations

a. =>50% of participants came from trials with high risk of bias
b. Heterogeneity: I^2=> 75%
c. 95% confidence interval crosses the null and also the minimal clinically important difference of 10 points
d. Heterogeneity: I^2=> 50%
e. 95% confidence interval crosses the minimal clinically important difference of 10 points
f. => 25% of participants came from trials with high risk of bias
g. 95% confidence interval crosses the null

5.5 GRADE outcomes for SNRI outcomes at primary end point, subgrouped by certainty of NP in participants

Certainty assessment							№ of patients		Effect		Certainty	Importance
№ of studies	Study design	Risk of bias	Inconsistency	Indirectness	Imprecision	Other considerations	SNRI	placebo or usual care	Relative (95% CI)	Absolute (95% CI)		
**SNRI pain outcomes - Possible / unlikely or unclear neuropathic pain**												
5	randomised trials	very serious[a]	not serious	not serious	not serious	none	810	653	-	MD **5.57 lower** (7.75 lower to 3.39 lower)	⨁⨁◯◯ Low	NOT IMPORTANT
**SNRI disability outcomes - Possible/ unlikely or unclear Neuropathic pain**												
5	randomised trials	serious[b]	not serious	not serious	not serious	none	810	655	-	MD **3.16 lower** (5.64 lower to 0.67 lower)	⨁⨁⨁◯ Moderate	NOT IMPORTANT

CI: confidence interval; **MD:** mean difference

Explanations

a. => 50% of participants from trials with high risk of bias
b. =>25% of participants from trials with high risk of bias

Online resource 6

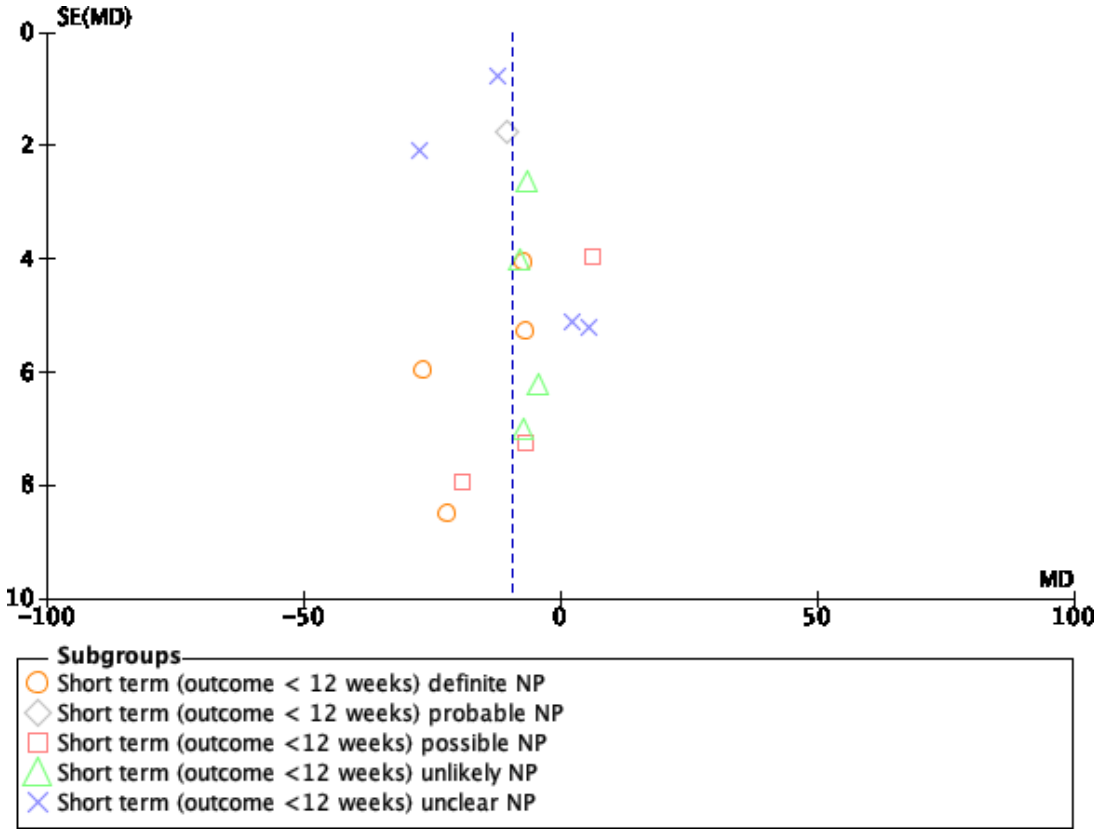

Online resource 6.1; Funnel plots for all pain outcomes

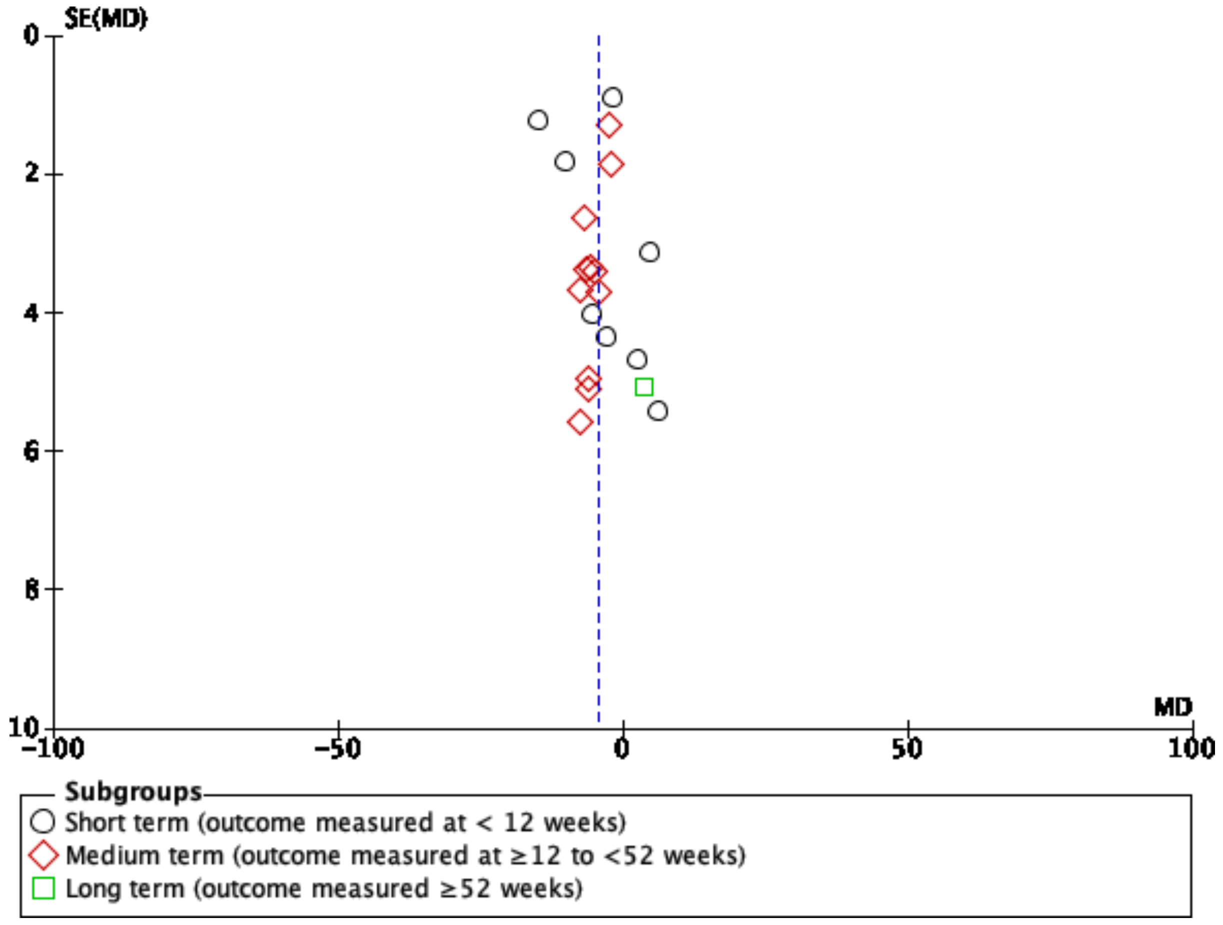

Online resource 6.2; Funnel plots for all disability outcome

Study or Subgroup	Treatment Mean	Treatment SD	Treatment Total	Control Mean	Control SD	Control Total	Weight	Mean Difference IV, Random, 95% CI
**3.1.1 Probable/definite neuropathic pain**								
TCA amitriptyline Vanelderen 2015;25 mg 2 wk	48	26.8	20	70	26.8	20	100.0%	-22.00 [-38.61, -5.39]
**Subtotal (95% CI)**			**20**			**20**	**100.0%**	**-22.00 [-38.61, -5.39]**
Heterogeneity: Not applicable								
Test for overall effect: Z = 2.60 (P = 0.009)								
**3.1.2 Possible/unlikely/unclear neuropathic pain**								
TCA amitriptyline Kurniawati 2020; 10mg 2 wk	-35.1	20.9	33	-40.7	20.4	30	16.9%	5.60 [-4.60, 15.80]
TCA amitriptyline Urquhart 2021;25mg 26 wk (nonNP)	29.1	20.34	38	32.9	20.79	27	16.9%	-3.80 [-13.96, 6.36]
TCA amitriptyline Urquhart 2021; 25mg 26 wk (NP)	26	18.36	12	46.2	24.18	18	9.4%	-20.20 [-35.45, -4.95]
TCA desipramine Atkinson 2007; 50-150ng/ml 12 wk	30	20.5	30	34	19	11	11.5%	-4.00 [-17.41, 9.41]
TCA desipramine Gould 2020; 15-65ng/ml 12 wk	37.75	28.15	37	43.6	27.45	33	12.0%	-5.85 [-18.89, 7.19]
TCA nortriptyline Atkinson 1998; 50-150mg 8 wk	-12.33	19.05	38	-4.33	16.33	40	22.7%	-8.00 [-15.89, -0.11]
TCA nortripytline Khoromi 2007; 25-100mg 7 wk	30	27	28	37	27	28	10.6%	-7.00 [-21.14, 7.14]
**Subtotal (95% CI)**			**216**			**187**	**100.0%**	**-5.32 [-10.55, -0.09]**
Heterogeneity: $Tau^2 = 15.07$; $Chi^2 = 8.68$, df = 6 (P = 0.19); $I^2 = 31\%$								
Test for overall effect: Z = 1.99 (P = 0.05)								

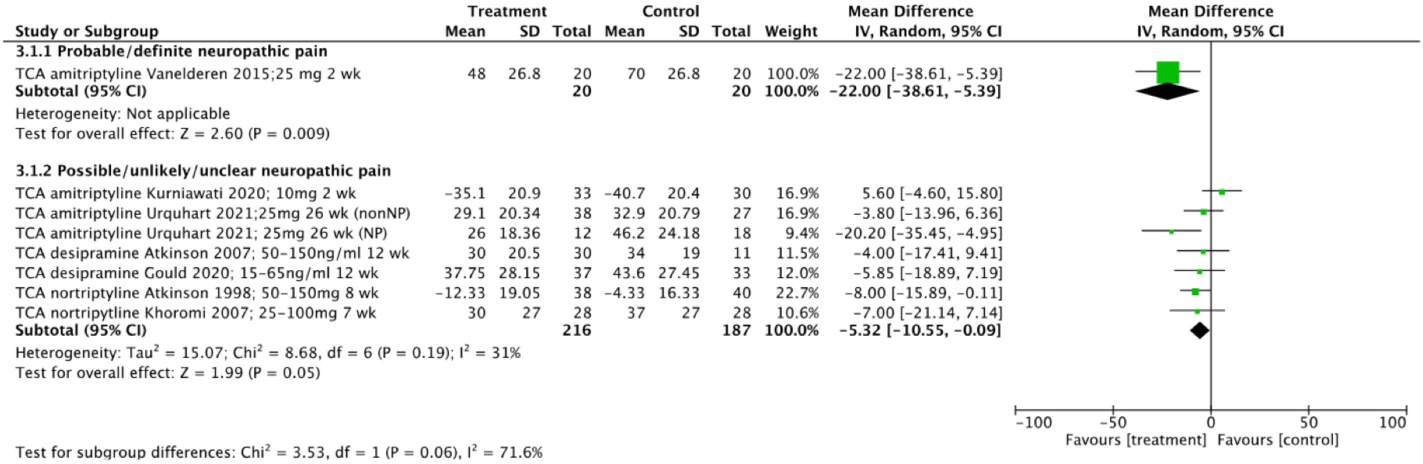

Test for subgroup differences: $Chi^2 = 3.53$, df = 1 (P = 0.06), $I^2 = 71.6\%$

Online resource 7.1; TCA pain outcomes (at primary end point), subgroups clustered by certainty of neuropathic pain (definite/probable versus possible/unclear/unlikely neuropathic pain)

Study or Subgroup	Treatment Mean	Treatment SD	Treatment Total	Control Mean	Control SD	Control Total	Weight	Mean Difference IV, Random, 95% CI
**3.2.1 Probable/definite neuropathic pain**								
**Subtotal (95% CI)**			**0**			**0**		**Not estimable**
Heterogeneity: Not applicable								
Test for overall effect: Not applicable								
**3.2.2 Possible/unlikely/unclear neuropathic pain**								
TCA amitriptyline Urquhart 2021;25mg 26 wk (nonNP)	19.58	17.98	38	19.58	17.32	27	15.3%	0.00 [−8.68, 8.68]
TCA amitriptyline Urquhart 2021; 25mg 26 wk (NP)	17.92	14.43	12	40	19.45	18	10.4%	−22.08 [−34.22, −9.94]
TCA desipramine Atkinson 2007; 50–150ng/ml 12 wk	9.6	10.4	30	17.1	10.4	11	18.1%	−7.50 [−14.68, −0.32]
TCA desipramine Gould 2020; 15–65ng/ml 12 wk	31.88	24.96	37	39.58	21.46	33	11.9%	−7.70 [−18.58, 3.18]
TCA nortriptyline Atkinson 1998; 50–150mg 8 wk	−2.85	4.14	38	−1.05	3.82	40	28.9%	−1.80 [−3.57, −0.03]
TCA nortripytline Khoromi 2007; 25–100mg 7 wk	27.5	16.7	28	30.5	15.9	28	15.5%	−3.00 [−11.54, 5.54]
**Subtotal (95% CI)**			**183**			**157**	**100.0%**	**−5.55 [−10.39, −0.71]**
Heterogeneity: $Tau^2 = 20.31$; $Chi^2 = 13.66$, df = 5 (P = 0.02); $I^2 = 63\%$								
Test for overall effect: Z = 2.25 (P = 0.02)								

Test for subgroup differences: Not applicable

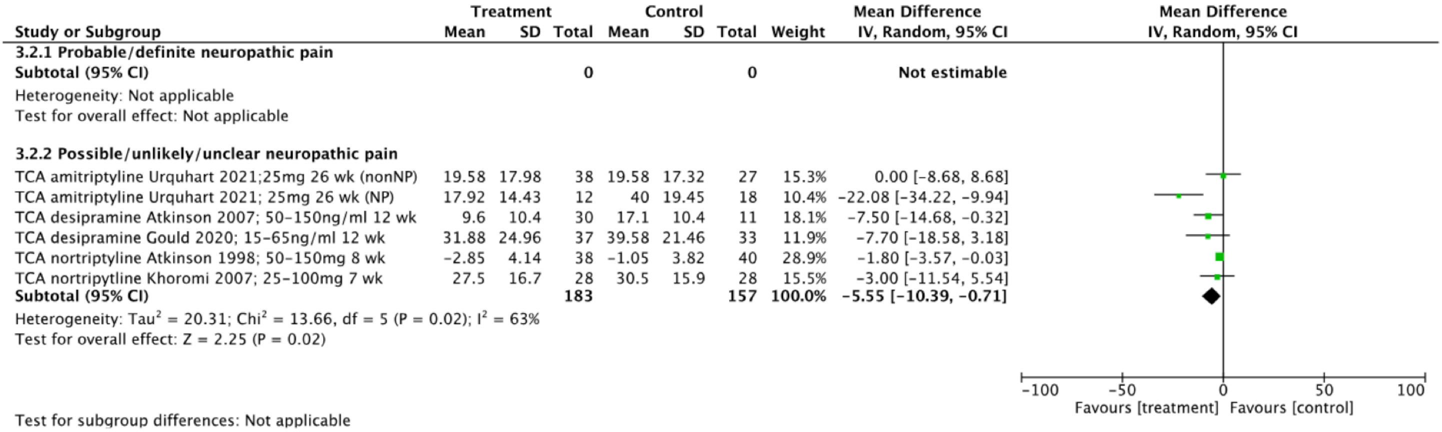

Online resource 7.2; TCA disability outcomes (at primary end point), subgroups clustered by certainty of neuropathic pain (definite/probable versus possible/unclear/unlikely neuropathic pain)

Study or Subgroup	Treatment Mean	Treatment SD	Treatment Total	Control Mean	Control SD	Control Total	Weight	Mean Difference IV, Random, 95% CI
**4.5.1 Probable/definite neuropathic pain**								
Gabapentin Yaksi 2007; 900-2400mg 8 wk	43	21	28	50	18	27	20.2%	-7.00 [-17.32, 3.32]
Gabapentin Yildirim 2003; 900-3600mg 8 wk	18.6	19.3	23	45.3	19.7	20	17.6%	-26.70 [-38.39, -15.01]
Pregaba+etoricoxib Yeole 2022; 75mg/60mg 8 wk	32.6	15.6	160	43.1	15.6	159	36.8%	-10.50 [-13.92, -7.08]
Pregaba+NSAID Nakashima 2019; 150mg/180mg 4 wk	20.5	14.3	26	27.7	16.1	30	25.4%	-7.20 [-15.16, 0.76]
**Subtotal (95% CI)**			**237**			**236**	**100.0%**	**-11.81 [-18.32, -5.31]**
Heterogeneity: $Tau^2 = 26.87$; $Chi^2 = 8.44$, df = 3 ($P = 0.04$); $I^2 = 64\%$								
Test for overall effect: $Z = 3.56$ ($P = 0.0004$)								
**4.5.2 Possible/unlikely/unclear neuropathic pain**								
Gabapentin Atkinson 2016; 3600mg 12 wk	35.1	22.9	36	41.2	22.6	36	13.5%	-6.10 [-16.61, 4.41]
Gabapentin McCleane 2000; 15mg/kg-1 6 wk	63.9	25	24	71.3	23.4	24	11.8%	-7.40 [-21.10, 6.30]
Gabapentin McCleane 2001; 1200mg 6 wk	59.2	26.1	31	63.6	23.9	34	12.6%	-4.40 [-16.60, 7.80]
Pregaba+buprenorphine Pota 2012; 150mg/35ug/h 3 wk	5.7	4.9	22	33.3	8.5	22	16.4%	-27.60 [-31.70, -23.50]
Pregaba+NSAID Chen 2022; 325mg/400mg 2 wk	15.5	3.7	71	27.7	5.2	71	16.9%	-12.20 [-13.68, -10.72]
Pregabalin Markman 2015; 75-150mg 2 wk	72	18.36	26	69.7	18.36	26	13.8%	2.30 [-7.68, 12.28]
Pregabalin Mathieson 2017; 75-300mg 8 wk	37	29	100	31	26	93	14.9%	6.00 [-1.76, 13.76]
**Subtotal (95% CI)**			**310**			**306**	**100.0%**	**-7.63 [-16.17, 0.91]**
Heterogeneity: $Tau^2 = 111.54$; $Chi^2 = 86.00$, df = 6 ($P < 0.00001$); $I^2 = 93\%$								
Test for overall effect: $Z = 1.75$ ($P = 0.08$)								

Test for subgroup differences: $Chi^2 = 0.58$, df = 1 ($P = 0.44$), $I^2 = 0\%$

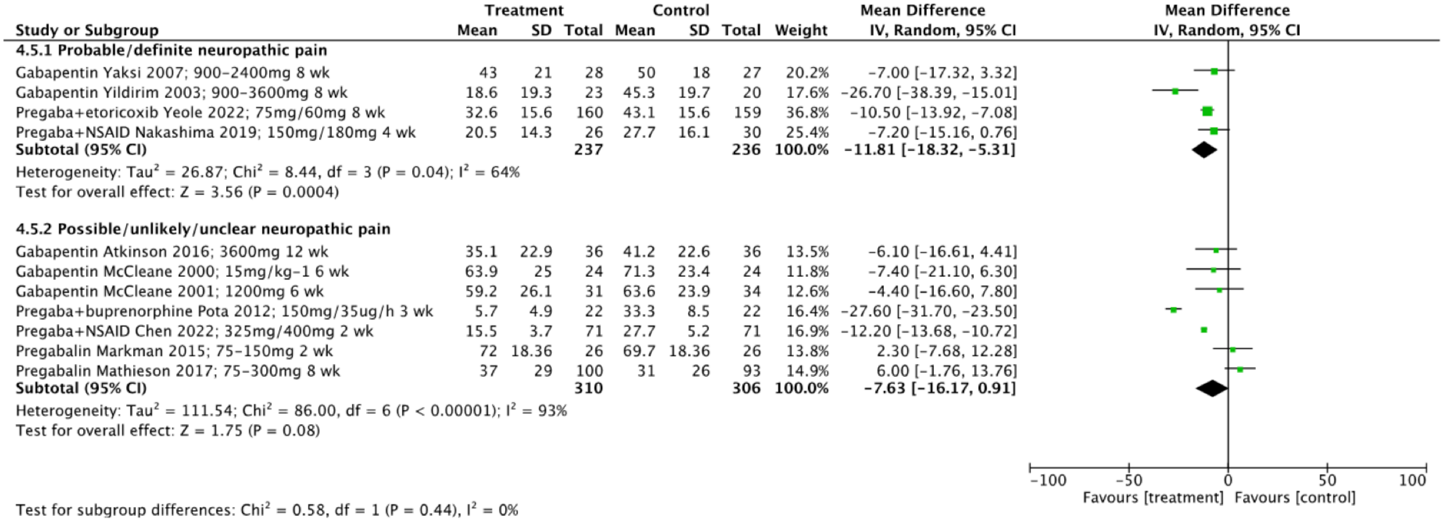

Online resource 8.1; Anticonvulsant pain outcomes (at primary end point), subgroups clustered by certainty of neuropathic pain (definite/probable versus possible/unclear/unlikely neuropathic pain

Study or Subgroup	Treatment Mean	Treatment SD	Treatment Total	Control Mean	Control SD	Control Total	Weight	Mean Difference IV, Random, 95% CI
**4.2.1 Probable/definite neuropathic pain**								
Gabapentin Haddadi 2016; 900mg 8 wk	30.42	16.07	27	36	14.09	30	17.8%	−5.58 [−13.46, 2.30]
Pregaba+etoricoxib Yeole 2022; 75mg/60mg 8 wk	22.21	14.42	160	32.25	18.17	159	82.2%	−10.04 [−13.64, −6.44]
**Subtotal (95% CI)**			**187**			**189**	**100.0%**	**−9.25 [−12.59, −5.90]**
Heterogeneity: $Tau^2 = 0.17$; $Chi^2 = 1.02$, $df = 1$ ($P = 0.31$); $I^2 = 2\%$								
Test for overall effect: $Z = 5.42$ ($P < 0.00001$)								
**4.2.2 Possible/unlikely/unclear neuropathic pain**								
Pregaba+NSAID Chen 2022; 325mg/400mg 2 wk	32.36	7.24	71	47.18	7.56	71	36.4%	−14.82 [−17.25, −12.39]
Pregabalin Markman 2015; 75–150mg 2 wk	54.08	19.54	26	47.83	19.54	26	31.2%	6.25 [−4.37, 16.87]
Pregabalin Mathieson 2017; 75–300mg 8 wk	39.57	32.17	93	36.96	30.87	89	32.4%	2.61 [−6.55, 11.77]
**Subtotal (95% CI)**			**190**			**186**	**100.0%**	**−2.60 [−17.78, 12.57]**
Heterogeneity: $Tau^2 = 163.00$; $Chi^2 = 25.87$, $df = 2$ ($P < 0.00001$); $I^2 = 92\%$								
Test for overall effect: $Z = 0.34$ ($P = 0.74$)								

Mean Difference IV, Random, 95% CI

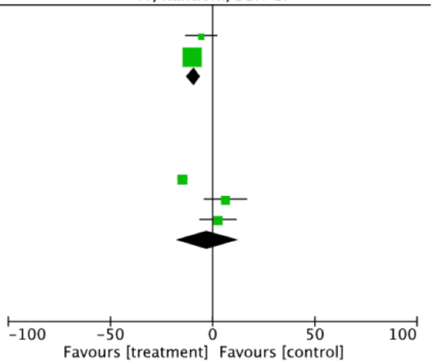

Test for subgroup differences: $Chi^2 = 0.70$, $df = 1$ ($P = 0.40$), $I^2 = 0\%$

Online resource 8.2; Anticonvulsant disability outcomes (at primary end point), subgroups clustered by certainty of neuropathic pain (definite/probable versus possible/unclear/unlikely neuropathic pain

Study or Subgroup	Treatment Mean	Treatment SD	Treatment Total	Control Mean	Control SD	Control Total	Weight	Mean Difference IV, Random, 95% CI
**5.5.1 Probable/definite neuropathic pain**								
**Subtotal (95% CI)**			**0**			**0**		**Not estimable**
Heterogeneity: Not applicable								
Test for overall effect: Not applicable								
**5.5.2 Possible/unlikely/unclear neuropathic pain**								
SNRI duloxetine Konno 2016; 20-60mg 14 wk	-24.3	15.9	209	-19.6	15.56	200	32.9%	-4.70 [-7.75, -1.65]
SNRI duloxetine Skljarevski 2009a; 20 mg 13 wk	-17.9	22.45	56	-18.7	13.56	38	8.2%	0.80 [-6.49, 8.09]
SNRI duloxetine Skljarevski 2009b; 60mg 13 wk	-25	22.86	108	-18.7	13.56	38	11.2%	-6.30 [-12.40, -0.20]
SNRI duloxetine Skljarevski 2009c; 120mg 13 wk	-24.5	22.86	108	-18.7	13.38	37	11.2%	-5.80 [-11.90, 0.30]
SNRI duloxetine Skljarevski 2010; 30- 120mg 12 wk	-23.2	21.14	109	-15	21.14	116	13.3%	-8.20 [-13.73, -2.67]
SNRI duloxetine Skljarevski 2010a; 60mg 12 wk	-22.5	20.95	195	-16.5	21.16	199	21.2%	-6.00 [-10.16, -1.84]
TCA duloxetine Schukro 2016; 30- 120mg 4 wk	41	29	25	60	27	25	1.9%	-19.00 [-34.53, -3.47]
**Subtotal (95% CI)**			**810**			**653**	**100.0%**	**-5.57 [-7.75, -3.39]**
Heterogeneity: Tau^2 = 1.34; Chi^2 = 7.08, df = 6 (P = 0.31); I^2 = 15%								
Test for overall effect: Z = 5.01 (P < 0.00001)								
Test for subgroup differences: Not applicable								

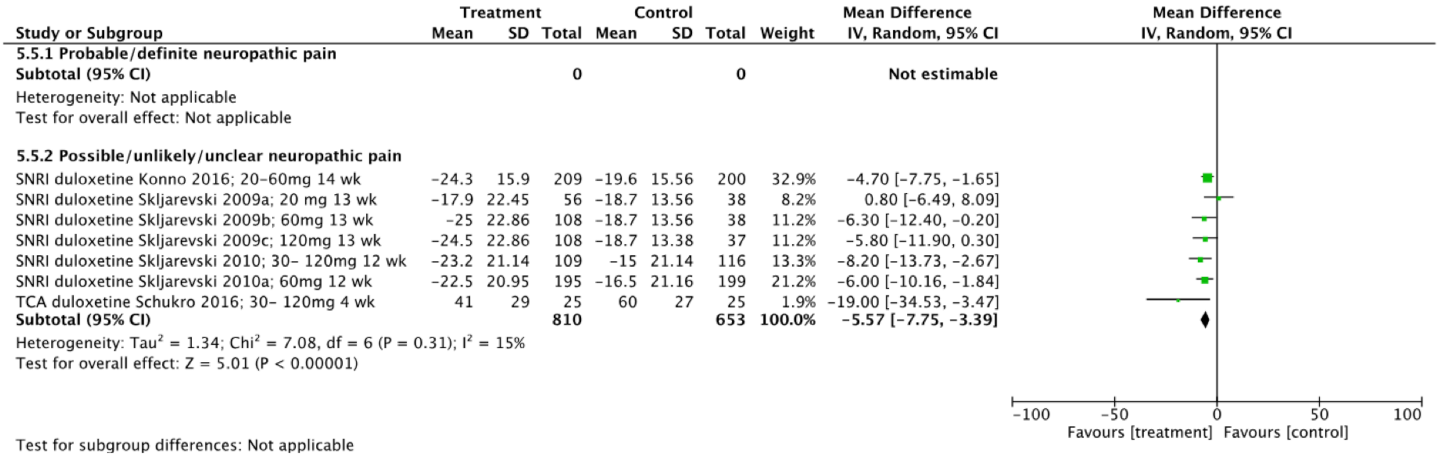

Online resource 9.1; SNRI pain outcomes (at primary end point), subgroups clustered by certainty of neuropathic pain (definite/probable versus possible/unclear/unlikely neuropathic pain)

Study or Subgroup	Treatment Mean	Treatment SD	Treatment Total	Control Mean	Control SD	Control Total	Weight	Mean Difference IV, Random, 95% CI
**5.2.1 Probable/definite neuropathic pain**								
**Subtotal (95% CI)**			**0**			**0**		**Not estimable**
Heterogeneity: Not applicable								
Test for overall effect: Not applicable								
**5.2.2 Possible/unlikely/unclear neuropathic pain**								
SNRI duloxetine Konno 2016; 20–60mg 14 wk	−16.08	13.9	230	−13.46	13.78	226	25.7%	−2.62 [−5.16, −0.08]
SNRI duloxetine Skljarevski 2009a; 20 mg 13 wk	−9.58	17.23	56	−5.4	17.84	38	8.8%	−4.18 [−11.43, 3.07]
SNRI duloxetine Skljarevski 2009b; 60mg 13 wk	−11.25	17.23	108	−5.4	17.84	38	10.2%	−5.85 [−12.39, 0.69]
SNRI duloxetine Skljarevski 2009c; 120mg 13 wk	−12.08	17.23	108	−5.4	17.84	37	10.1%	−6.68 [−13.28, −0.08]
SNRI duloxetine Skljarevski 2010; 30– 120mg 12 wk	−15	19.79	109	−8.04	19.79	116	13.9%	−6.96 [−12.13, −1.79]
SNRI duloxetine Skljarevski 2010a; 60mg 12 wk	−11.2	17.23	178	−9.2	17.84	179	20.0%	−2.00 [−5.64, 1.64]
TCA duloxetine Schukro 2016; 30– 120mg 4 wk	36	10.9	21	31.3	9.3	21	11.2%	4.70 [−1.43, 10.83]
**Subtotal (95% CI)**			**810**			**655**	**100.0%**	**−3.16 [−5.64, −0.67]**
Heterogeneity: $Tau^2 = 4.59$; $Chi^2 = 10.71$, df = 6 (P = 0.10); $I^2 = 44\%$								
Test for overall effect: Z = 2.49 (P = 0.01)								

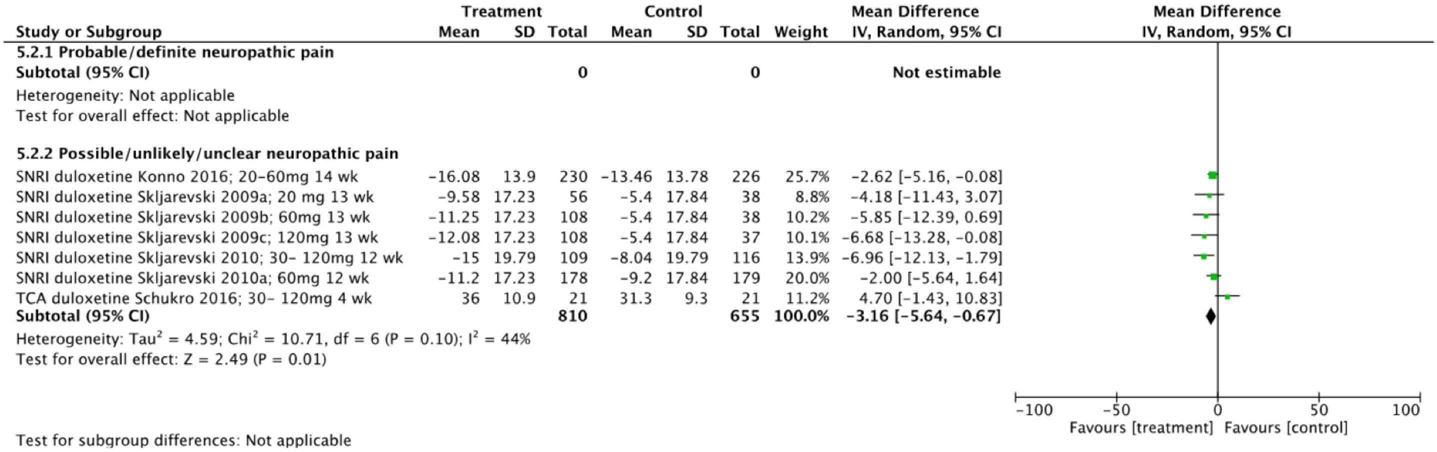

Test for subgroup differences: Not applicable

Online resource 9.2; SNRI disability outcomes (at primary end point), subgroups clustered by certainty of neuropathic pain (definite/probable versus possible/unclear/unlikely neuropathic pain

Online resource 10; Meta-regression of neuropathic pain factors influencing outcomes

Category	Variable	Pain short term		Pain med term		Disability med term		Anti-Convulsant	
Factors related to;		Estimate (95% CI)	P-value	Estimate (95% CI)	P-value	Estimate (95% CI)	P-value	Estimate (95% CI)	P-value
Neur Pain (clustered)	Possible/unclear/ Unlikely	reference	reference	reference	reference	reference	reference	reference	reference
	Definite/probable	-6.367 (−17.348 to 4.615)	0.256	-12.753 (−25.588 to 0.081)	0.051*	-1.819 (−12.044 to 8.407)	0.727	-4.718 (−18.198 to 8.761)	0.493
	Omnibus tests Q value (p)	1.291	0.256	3.793	0.051*	0.121	0.727	0.471	0.493
